# Correction: Pre-colonial Amerindian legacies in forest composition of southern Brazil

**DOI:** 10.1371/journal.pone.0269056

**Published:** 2022-05-23

**Authors:** Aline Pereira Cruz, Eduardo Luis Hettwer Giehl, Carolina Levis, Juliana Salles Machado, Lucas Bueno, Nivaldo Peroni

The second author’s name is spelled incorrectly. The correct name is: Eduardo Luis Hettwer Giehl.
