# Supplementary material for: Pre-colonial Amerindian legacies in forest composition of southern Brazil
Source: PLoS One. 2020 Jul 23;15(7):e0235819. doi: 10.1371/journal.pone.0235819 (PMC7377383; doi:10.1371/journal.pone.0235819)
Supplement: S1 Code — This file contains all the codes wrote in R. (DOCX) [file pone.0235819.s001.docx]

R Scripts - Pre-colonial Amerindian legacies in forest composition of southern Brazil

Aline Pereira Cruz, Eduardo Luis Hettwer Giehl, Carolina Levis, Juliana Salles Machado, Lucas Bueno, Nivaldo Peroni

February 26, 2020

## S4 - Redundancy Analysis (RDA)

library(vegan)

## Loading required package: permute

## Loading required package: lattice

## This is vegan 2.5-3

library(RColorBrewer)
library(plyr)


sp <- read.table("S4_sps.csv", header=TRUE, sep=",", dec=".")
sp<-sp[,-1]
#sp<-as.data.frame(sp)
#row.names(sp)=as.character(sp[,1])
#sp<-as.matrix(sp)
co_occ<-read.table ("S4_cult.csv",header=TRUE, sep=",", dec=".")
co_occ<-co_occ[,-1]
#co_occ<-as.data.frame(co_occ)
#row.names(co_occ)=as.character(co_occ[,1])
#co_occ<-as.matrix(co_occ)
#removing rares
sp<-sp[,colSums(sp)>=30]
dim(sp[,colSums(decostand(sp, method="pa"))>=5])

## [1] 417 327

#Hellinger transformation of species matrix
sp_hel<-decostand(sp, method= 'hellinger')

ord_sp<-rda(sp_hel~jes+guaranis, data=co_occ)
summary(ord_sp)

##
## Call:
## rda(formula = sp_hel ~ jes + guaranis, data = co_occ)
##
## Partitioning of variance:
## Inertia Proportion
## Total 0.80510 1.00000
## Constrained 0.05584 0.06936
## Unconstrained 0.74926 0.93064
##
## Eigenvalues, and their contribution to the variance
##
## Importance of components:
## RDA1 RDA2 PC1 PC2 PC3 PC4
## Eigenvalue 0.04174 0.01410 0.08045 0.05704 0.03464 0.03175
## Proportion Explained 0.05185 0.01752 0.09993 0.07085 0.04303 0.03943
## Cumulative Proportion 0.05185 0.06936 0.16929 0.24014 0.28317 0.32260
## PC5 PC6 PC7 PC8 PC9 PC10
## Eigenvalue 0.02542 0.01931 0.01887 0.01541 0.01511 0.01299
## Proportion Explained 0.03158 0.02399 0.02344 0.01914 0.01877 0.01614
## Cumulative Proportion 0.35418 0.37817 0.40161 0.42075 0.43952 0.45566
## PC11 PC12 PC13 PC14 PC15 PC16
## Eigenvalue 0.01158 0.01032 0.009857 0.009119 0.008691 0.008465
## Proportion Explained 0.01438 0.01282 0.012243 0.011326 0.010795 0.010515
## Cumulative Proportion 0.47004 0.48286 0.495104 0.506430 0.517224 0.527739
## PC17 PC18 PC19 PC20 PC21
## Eigenvalue 0.008223 0.007980 0.007828 0.007389 0.007110
## Proportion Explained 0.010214 0.009911 0.009723 0.009178 0.008831
## Cumulative Proportion 0.537953 0.547864 0.557587 0.566765 0.575596
## PC22 PC23 PC24 PC25 PC26
## Eigenvalue 0.007026 0.006706 0.006531 0.006251 0.005930
## Proportion Explained 0.008727 0.008329 0.008112 0.007764 0.007365
## Cumulative Proportion 0.584323 0.592652 0.600764 0.608528 0.615893
## PC27 PC28 PC29 PC30 PC31
## Eigenvalue 0.005644 0.005467 0.005359 0.005256 0.005120
## Proportion Explained 0.007010 0.006790 0.006656 0.006529 0.006359
## Cumulative Proportion 0.622903 0.629693 0.636349 0.642878 0.649237
## PC32 PC33 PC34 PC35 PC36
## Eigenvalue 0.004988 0.004775 0.004614 0.004490 0.004390
## Proportion Explained 0.006196 0.005931 0.005731 0.005576 0.005453
## Cumulative Proportion 0.655433 0.661364 0.667095 0.672672 0.678124
## PC37 PC38 PC39 PC40 PC41 PC42
## Eigenvalue 0.004350 0.004313 0.00417 0.003993 0.003966 0.003934
## Proportion Explained 0.005404 0.005357 0.00518 0.004960 0.004926 0.004887
## Cumulative Proportion 0.683528 0.688885 0.69407 0.699025 0.703951 0.708838
## PC43 PC44 PC45 PC46 PC47
## Eigenvalue 0.003781 0.003722 0.003642 0.003574 0.003531
## Proportion Explained 0.004697 0.004623 0.004524 0.004439 0.004386
## Cumulative Proportion 0.713535 0.718158 0.722682 0.727120 0.731507
## PC48 PC49 PC50 PC51 PC52
## Eigenvalue 0.003444 0.003375 0.003242 0.003191 0.003156
## Proportion Explained 0.004278 0.004193 0.004026 0.003963 0.003920
## Cumulative Proportion 0.735784 0.739977 0.744003 0.747966 0.751886
## PC53 PC54 PC55 PC56 PC57
## Eigenvalue 0.003109 0.003066 0.003009 0.002983 0.002883
## Proportion Explained 0.003862 0.003809 0.003737 0.003706 0.003581
## Cumulative Proportion 0.755748 0.759557 0.763294 0.766999 0.770581
## PC58 PC59 PC60 PC61 PC62
## Eigenvalue 0.002807 0.002789 0.002742 0.002711 0.002627
## Proportion Explained 0.003486 0.003464 0.003406 0.003368 0.003263
## Cumulative Proportion 0.774067 0.777532 0.780938 0.784306 0.787568
## PC63 PC64 PC65 PC66 PC67
## Eigenvalue 0.002555 0.002534 0.002526 0.002453 0.002405
## Proportion Explained 0.003173 0.003147 0.003137 0.003046 0.002988
## Cumulative Proportion 0.790741 0.793888 0.797025 0.800071 0.803059
## PC68 PC69 PC70 PC71 PC72
## Eigenvalue 0.002367 0.002351 0.002324 0.002274 0.002216
## Proportion Explained 0.002940 0.002920 0.002887 0.002824 0.002752
## Cumulative Proportion 0.806000 0.808920 0.811807 0.814631 0.817383
## PC73 PC74 PC75 PC76 PC77
## Eigenvalue 0.002208 0.002167 0.002135 0.002120 0.002074
## Proportion Explained 0.002743 0.002692 0.002652 0.002634 0.002576
## Cumulative Proportion 0.820126 0.822818 0.825470 0.828103 0.830680
## PC78 PC79 PC80 PC81 PC82
## Eigenvalue 0.002050 0.002018 0.001969 0.001958 0.001945
## Proportion Explained 0.002546 0.002507 0.002445 0.002432 0.002416
## Cumulative Proportion 0.833226 0.835732 0.838178 0.840609 0.843025
## PC83 PC84 PC85 PC86 PC87
## Eigenvalue 0.001926 0.001858 0.001826 0.001786 0.001759
## Proportion Explained 0.002392 0.002307 0.002268 0.002218 0.002185
## Cumulative Proportion 0.845417 0.847724 0.849992 0.852210 0.854395
## PC88 PC89 PC90 PC91 PC92
## Eigenvalue 0.001755 0.001722 0.001707 0.001676 0.001643
## Proportion Explained 0.002180 0.002139 0.002120 0.002082 0.002041
## Cumulative Proportion 0.856575 0.858714 0.860834 0.862917 0.864958
## PC93 PC94 PC95 PC96 PC97
## Eigenvalue 0.001595 0.001572 0.001560 0.001544 0.001522
## Proportion Explained 0.001981 0.001952 0.001938 0.001917 0.001891
## Cumulative Proportion 0.866939 0.868891 0.870829 0.872746 0.874637
## PC98 PC99 PC100 PC101 PC102
## Eigenvalue 0.001486 0.001465 0.001455 0.001425 0.001412
## Proportion Explained 0.001846 0.001820 0.001807 0.001770 0.001754
## Cumulative Proportion 0.876482 0.878302 0.880109 0.881880 0.883633
## PC103 PC104 PC105 PC106 PC107
## Eigenvalue 0.001379 0.001376 0.001360 0.001355 0.001318
## Proportion Explained 0.001713 0.001709 0.001689 0.001682 0.001637
## Cumulative Proportion 0.885346 0.887055 0.888744 0.890426 0.892063
## PC108 PC109 PC110 PC111 PC112
## Eigenvalue 0.001312 0.001281 0.001267 0.001254 0.001237
## Proportion Explained 0.001630 0.001591 0.001574 0.001557 0.001537
## Cumulative Proportion 0.893693 0.895284 0.896858 0.898415 0.899952
## PC113 PC114 PC115 PC116 PC117
## Eigenvalue 0.001221 0.001202 0.001186 0.001162 0.001146
## Proportion Explained 0.001516 0.001493 0.001473 0.001443 0.001423
## Cumulative Proportion 0.901468 0.902960 0.904433 0.905877 0.907300
## PC118 PC119 PC120 PC121 PC122 PC123
## Eigenvalue 0.001137 0.001113 0.001104 0.001085 0.00107 0.001065
## Proportion Explained 0.001412 0.001382 0.001371 0.001348 0.00133 0.001323
## Cumulative Proportion 0.908712 0.910094 0.911465 0.912813 0.91414 0.915465
## PC124 PC125 PC126 PC127 PC128
## Eigenvalue 0.001037 0.001031 0.001025 0.0009974 0.000993
## Proportion Explained 0.001288 0.001281 0.001273 0.0012388 0.001233
## Cumulative Proportion 0.916753 0.918034 0.919307 0.9205458 0.921779
## PC129 PC130 PC131 PC132 PC133
## Eigenvalue 0.0009803 0.000969 0.0009588 0.0009412 0.0009251
## Proportion Explained 0.0012177 0.001204 0.0011910 0.0011691 0.0011490
## Cumulative Proportion 0.9229969 0.924200 0.9253914 0.9265604 0.9277095
## PC134 PC135 PC136 PC137 PC138
## Eigenvalue 0.0008992 0.0008943 0.0008856 0.0008797 0.0008701
## Proportion Explained 0.0011168 0.0011107 0.0011000 0.0010927 0.0010807
## Cumulative Proportion 0.9288263 0.9299370 0.9310371 0.9321298 0.9332105
## PC139 PC140 PC141 PC142 PC143
## Eigenvalue 0.0008577 0.000848 0.0008377 0.000822 0.0008118
## Proportion Explained 0.0010653 0.001053 0.0010404 0.001021 0.0010083
## Cumulative Proportion 0.9342758 0.935329 0.9363696 0.937391 0.9383989
## PC144 PC145 PC146 PC147 PC148
## Eigenvalue 0.0007980 0.0007793 0.0007711 0.0007652 0.0007547
## Proportion Explained 0.0009912 0.0009679 0.0009577 0.0009504 0.0009374
## Cumulative Proportion 0.9393901 0.9403581 0.9413158 0.9422662 0.9432036
## PC149 PC150 PC151 PC152 PC153
## Eigenvalue 0.0007475 0.000744 0.0007360 0.0007226 0.0007172
## Proportion Explained 0.0009284 0.000924 0.0009142 0.0008975 0.0008908
## Cumulative Proportion 0.9441320 0.945056 0.9459702 0.9468677 0.9477585
## PC154 PC155 PC156 PC157 PC158
## Eigenvalue 0.0006997 0.0006864 0.0006828 0.0006752 0.0006626
## Proportion Explained 0.0008691 0.0008526 0.0008481 0.0008387 0.0008230
## Cumulative Proportion 0.9486276 0.9494802 0.9503283 0.9511670 0.9519900
## PC159 PC160 PC161 PC162 PC163
## Eigenvalue 0.0006577 0.0006376 0.0006310 0.0006286 0.0006201
## Proportion Explained 0.0008169 0.0007920 0.0007838 0.0007808 0.0007702
## Cumulative Proportion 0.9528068 0.9535989 0.9543826 0.9551634 0.9559336
## PC164 PC165 PC166 PC167 PC168
## Eigenvalue 0.0006174 0.0006047 0.0006014 0.0005941 0.0005870
## Proportion Explained 0.0007669 0.0007511 0.0007470 0.0007379 0.0007291
## Cumulative Proportion 0.9567005 0.9574516 0.9581985 0.9589364 0.9596655
## PC169 PC170 PC171 PC172 PC173
## Eigenvalue 0.0005809 0.0005705 0.0005616 0.0005543 0.0005428
## Proportion Explained 0.0007215 0.0007087 0.0006975 0.0006885 0.0006742
## Cumulative Proportion 0.9603870 0.9610957 0.9617932 0.9624817 0.9631559
## PC174 PC175 PC176 PC177 PC178
## Eigenvalue 0.0005343 0.0005269 0.0005209 0.0005126 0.0005111
## Proportion Explained 0.0006637 0.0006545 0.0006470 0.0006367 0.0006348
## Cumulative Proportion 0.9638196 0.9644741 0.9651211 0.9657578 0.9663926
## PC179 PC180 PC181 PC182 PC183
## Eigenvalue 0.0005009 0.0004997 0.0004898 0.0004883 0.0004775
## Proportion Explained 0.0006222 0.0006207 0.0006083 0.0006065 0.0005931
## Cumulative Proportion 0.9670148 0.9676355 0.9682438 0.9688503 0.9694434
## PC184 PC185 PC186 PC187 PC188
## Eigenvalue 0.0004638 0.0004586 0.0004546 0.0004469 0.0004413
## Proportion Explained 0.0005761 0.0005696 0.0005646 0.0005550 0.0005482
## Cumulative Proportion 0.9700195 0.9705890 0.9711536 0.9717087 0.9722569
## PC189 PC190 PC191 PC192 PC193
## Eigenvalue 0.0004310 0.0004274 0.0004244 0.0004210 0.0004116
## Proportion Explained 0.0005354 0.0005308 0.0005272 0.0005229 0.0005112
## Cumulative Proportion 0.9727922 0.9733231 0.9738502 0.9743731 0.9748843
## PC194 PC195 PC196 PC197 PC198
## Eigenvalue 0.0004027 0.0003981 0.0003917 0.0003901 0.0003806
## Proportion Explained 0.0005002 0.0004945 0.0004865 0.0004845 0.0004727
## Cumulative Proportion 0.9753844 0.9758789 0.9763654 0.9768499 0.9773226
## PC199 PC200 PC201 PC202 PC203
## Eigenvalue 0.0003762 0.0003712 0.0003667 0.0003630 0.0003552
## Proportion Explained 0.0004673 0.0004610 0.0004555 0.0004509 0.0004412
## Cumulative Proportion 0.9777899 0.9782509 0.9787064 0.9791573 0.9795984
## PC204 PC205 PC206 PC207 PC208
## Eigenvalue 0.0003499 0.0003484 0.0003452 0.0003424 0.0003340
## Proportion Explained 0.0004346 0.0004328 0.0004287 0.0004253 0.0004149
## Cumulative Proportion 0.9800330 0.9804658 0.9808946 0.9813198 0.9817348
## PC209 PC210 PC211 PC212 PC213
## Eigenvalue 0.0003260 0.0003155 0.0003138 0.0003104 0.0003058
## Proportion Explained 0.0004049 0.0003919 0.0003897 0.0003855 0.0003798
## Cumulative Proportion 0.9821396 0.9825315 0.9829213 0.9833068 0.9836866
## PC214 PC215 PC216 PC217 PC218
## Eigenvalue 0.0003029 0.0002985 0.0002918 0.0002876 0.0002838
## Proportion Explained 0.0003762 0.0003708 0.0003624 0.0003572 0.0003525
## Cumulative Proportion 0.9840629 0.9844336 0.9847961 0.9851533 0.9855057
## PC219 PC220 PC221 PC222 PC223
## Eigenvalue 0.0002813 0.0002700 0.0002676 0.0002633 0.0002584
## Proportion Explained 0.0003494 0.0003354 0.0003324 0.0003271 0.0003209
## Cumulative Proportion 0.9858551 0.9861905 0.9865229 0.9868499 0.9871709
## PC224 PC225 PC226 PC227 PC228
## Eigenvalue 0.0002522 0.0002450 0.0002435 0.0002366 0.0002318
## Proportion Explained 0.0003132 0.0003043 0.0003024 0.0002939 0.0002879
## Cumulative Proportion 0.9874841 0.9877884 0.9880908 0.9883847 0.9886726
## PC229 PC230 PC231 PC232 PC233
## Eigenvalue 0.0002278 0.0002235 0.0002225 0.0002183 0.0002102
## Proportion Explained 0.0002830 0.0002776 0.0002764 0.0002712 0.0002611
## Cumulative Proportion 0.9889556 0.9892332 0.9895096 0.9897807 0.9900418
## PC234 PC235 PC236 PC237 PC238
## Eigenvalue 0.0002062 0.0002031 0.0002006 0.0001982 0.0001952
## Proportion Explained 0.0002561 0.0002523 0.0002492 0.0002461 0.0002425
## Cumulative Proportion 0.9902979 0.9905502 0.9907994 0.9910455 0.9912880
## PC239 PC240 PC241 PC242 PC243
## Eigenvalue 0.0001892 0.0001881 0.0001813 0.0001792 0.0001749
## Proportion Explained 0.0002350 0.0002336 0.0002252 0.0002225 0.0002172
## Cumulative Proportion 0.9915230 0.9917566 0.9919817 0.9922043 0.9924215
## PC244 PC245 PC246 PC247 PC248
## Eigenvalue 0.0001740 0.0001669 0.0001633 0.0001624 0.0001614
## Proportion Explained 0.0002162 0.0002073 0.0002029 0.0002017 0.0002005
## Cumulative Proportion 0.9926377 0.9928450 0.9930479 0.9932496 0.9934501
## PC249 PC250 PC251 PC252 PC253
## Eigenvalue 0.0001566 0.000153 0.0001506 0.0001460 0.0001438
## Proportion Explained 0.0001945 0.000190 0.0001871 0.0001814 0.0001786
## Cumulative Proportion 0.9936446 0.993835 0.9940217 0.9942030 0.9943816
## PC254 PC255 PC256 PC257 PC258
## Eigenvalue 0.0001427 0.0001393 0.0001373 0.0001331 0.0001279
## Proportion Explained 0.0001773 0.0001731 0.0001706 0.0001653 0.0001589
## Cumulative Proportion 0.9945589 0.9947319 0.9949025 0.9950679 0.9952267
## PC259 PC260 PC261 PC262 PC263
## Eigenvalue 0.0001253 0.0001230 0.0001209 0.0001201 0.0001158
## Proportion Explained 0.0001556 0.0001527 0.0001502 0.0001492 0.0001439
## Cumulative Proportion 0.9953823 0.9955350 0.9956852 0.9958344 0.9959783
## PC264 PC265 PC266 PC267 PC268
## Eigenvalue 0.0001123 0.0001102 0.0001071 0.0001057 0.000103
## Proportion Explained 0.0001395 0.0001369 0.0001330 0.0001313 0.000128
## Cumulative Proportion 0.9961179 0.9962547 0.9963877 0.9965190 0.996647
## PC269 PC270 PC271 PC272 PC273
## Eigenvalue 9.899e-05 9.768e-05 9.631e-05 9.263e-05 0.0000918
## Proportion Explained 1.229e-04 1.213e-04 1.196e-04 1.151e-04 0.0001140
## Cumulative Proportion 9.968e-01 9.969e-01 9.970e-01 9.971e-01 0.9972400
## PC274 PC275 PC276 PC277 PC278
## Eigenvalue 8.838e-05 8.641e-05 8.342e-05 8.040e-05 7.646e-05
## Proportion Explained 1.098e-04 1.073e-04 1.036e-04 9.986e-05 9.497e-05
## Cumulative Proportion 9.973e-01 9.975e-01 9.976e-01 9.977e-01 9.978e-01
## PC279 PC280 PC281 PC282 PC283
## Eigenvalue 7.588e-05 7.362e-05 6.945e-05 6.901e-05 6.701e-05
## Proportion Explained 9.424e-05 9.145e-05 8.626e-05 8.572e-05 8.323e-05
## Cumulative Proportion 9.978e-01 9.979e-01 9.980e-01 9.981e-01 9.982e-01
## PC284 PC285 PC286 PC287 PC288
## Eigenvalue 6.609e-05 6.493e-05 6.293e-05 6.098e-05 5.939e-05
## Proportion Explained 8.209e-05 8.065e-05 7.816e-05 7.574e-05 7.376e-05
## Cumulative Proportion 9.983e-01 9.984e-01 9.984e-01 9.985e-01 9.986e-01
## PC289 PC290 PC291 PC292 PC293
## Eigenvalue 5.737e-05 5.505e-05 5.451e-05 5.083e-05 4.847e-05
## Proportion Explained 7.125e-05 6.838e-05 6.770e-05 6.314e-05 6.021e-05
## Cumulative Proportion 9.987e-01 9.987e-01 9.988e-01 9.989e-01 9.989e-01
## PC294 PC295 PC296 PC297 PC298
## Eigenvalue 4.613e-05 4.590e-05 4.529e-05 4.179e-05 4.060e-05
## Proportion Explained 5.729e-05 5.701e-05 5.625e-05 5.190e-05 5.042e-05
## Cumulative Proportion 9.990e-01 9.990e-01 9.991e-01 9.991e-01 9.992e-01
## PC299 PC300 PC301 PC302 PC303
## Eigenvalue 3.944e-05 3.758e-05 3.563e-05 3.545e-05 3.413e-05
## Proportion Explained 4.899e-05 4.667e-05 4.425e-05 4.404e-05 4.239e-05
## Cumulative Proportion 9.992e-01 9.993e-01 9.993e-01 9.994e-01 9.994e-01
## PC304 PC305 PC306 PC307 PC308
## Eigenvalue 3.250e-05 3.059e-05 2.984e-05 2.831e-05 2.722e-05
## Proportion Explained 4.036e-05 3.800e-05 3.706e-05 3.516e-05 3.382e-05
## Cumulative Proportion 9.995e-01 9.995e-01 9.995e-01 9.996e-01 9.996e-01
## PC309 PC310 PC311 PC312 PC313
## Eigenvalue 2.661e-05 2.596e-05 2.468e-05 2.289e-05 2.179e-05
## Proportion Explained 3.305e-05 3.225e-05 3.065e-05 2.843e-05 2.706e-05
## Cumulative Proportion 9.996e-01 9.997e-01 9.997e-01 9.997e-01 9.998e-01
## PC314 PC315 PC316 PC317 PC318
## Eigenvalue 2.042e-05 2.023e-05 1.833e-05 1.707e-05 1.538e-05
## Proportion Explained 2.537e-05 2.513e-05 2.277e-05 2.120e-05 1.910e-05
## Cumulative Proportion 9.998e-01 9.998e-01 9.998e-01 9.998e-01 9.999e-01
## PC319 PC320 PC321 PC322 PC323
## Eigenvalue 1.373e-05 1.319e-05 1.236e-05 1.151e-05 9.331e-06
## Proportion Explained 1.706e-05 1.638e-05 1.535e-05 1.429e-05 1.159e-05
## Cumulative Proportion 9.999e-01 9.999e-01 9.999e-01 9.999e-01 9.999e-01
## PC324 PC325 PC326 PC327 PC328
## Eigenvalue 9.155e-06 8.565e-06 7.485e-06 6.718e-06 6.164e-06
## Proportion Explained 1.137e-05 1.064e-05 9.296e-06 8.345e-06 7.656e-06
## Cumulative Proportion 1.000e+00 1.000e+00 1.000e+00 1.000e+00 1.000e+00
## PC329 PC330
## Eigenvalue 5.084e-06 4.488e-06
## Proportion Explained 6.315e-06 5.574e-06
## Cumulative Proportion 1.000e+00 1.000e+00
##
## Accumulated constrained eigenvalues
## Importance of components:
## RDA1 RDA2
## Eigenvalue 0.04174 0.0141
## Proportion Explained 0.74745 0.2526
## Cumulative Proportion 0.74745 1.0000
##
## Scaling 2 for species and site scores
## * Species are scaled proportional to eigenvalues
## * Sites are unscaled: weighted dispersion equal on all dimensions
## * General scaling constant of scores: 4.277954
##
##
## Species scores
##
## RDA1 RDA2 PC1 PC2 PC3 PC4
## sp1 -3.025e-02 -1.786e-02 0.0318724 0.0133461 -0.0117160 5.231e-03
## sp2 4.051e-02 -1.074e-02 0.0116996 -0.0097070 -0.0633444 6.575e-03
## sp4 -2.528e-02 1.730e-02 -0.0244339 0.0305999 -0.0033929 -2.538e-02
## sp7 -8.604e-03 -3.420e-03 0.0185763 0.0094973 0.0075971 4.523e-03
## sp11 -4.508e-02 1.038e-03 0.0269599 0.0171769 -0.0301715 -2.502e-02
## sp13 7.627e-03 3.742e-02 -0.0670165 0.0435151 0.0169792 9.583e-03
## sp15 -2.528e-02 -8.340e-04 0.0247689 0.0311814 -0.0305853 -2.902e-02
## sp16 -1.129e-02 -2.263e-03 0.0400256 0.0479771 0.0239607 9.795e-03
## sp17 -1.453e-01 -3.985e-02 0.2412503 0.1901762 0.0196478 1.474e-02
## sp19 5.967e-02 3.442e-02 -0.1512706 0.0385280 0.0335380 -3.371e-02
## sp20 1.821e-02 -2.779e-02 -0.0073556 -0.0253069 0.0073175 -1.790e-02
## sp22 1.851e-03 1.958e-02 -0.0290667 0.0007131 -0.0014881 8.085e-03
## sp23 -3.980e-02 5.543e-02 -0.0544779 -0.0040329 0.0127867 -1.164e-02
## sp24 -1.189e-02 1.552e-02 0.0249836 0.0189037 0.0091398 1.070e-03
## sp25 -5.780e-02 -1.116e-02 0.3629940 0.2976265 0.1942585 1.265e-01
## sp26 -5.737e-02 -1.678e-02 0.0311285 0.0009201 -0.0229910 -3.336e-03
## sp28 -4.412e-02 -2.366e-02 0.0146115 -0.0042701 -0.0152538 -2.353e-03
## sp29 -7.808e-02 -1.779e-02 0.0787695 0.0361570 -0.0322121 -6.566e-03
## sp31 -3.207e-02 5.328e-03 0.0248717 0.0185552 -0.0158212 -1.457e-02
## sp33 1.320e-02 1.359e-02 -0.0187736 0.0328763 0.0236978 -2.768e-02
## sp35 1.276e-02 -5.810e-03 -0.0170668 0.0113177 -0.0001176 9.253e-04
## sp36 -9.384e-02 -2.962e-02 0.0783674 0.0769085 -0.0794413 -7.734e-02
## sp37 1.091e-02 4.889e-02 -0.1114898 0.0540353 0.0141182 5.807e-03
## sp38 -3.901e-02 -1.719e-02 0.0217904 0.0056147 -0.0335332 -1.988e-02
## sp39 -1.561e-02 3.288e-02 -0.0612757 0.0210812 0.0153257 -1.637e-02
## sp41 2.669e-01 -1.375e-01 -0.0250046 -0.2916993 -0.2750219 8.149e-02
## sp43 -5.788e-02 1.955e-02 0.0405300 0.1197945 0.0006800 2.761e-02
## sp45 4.182e-04 1.596e-03 0.0158896 0.0135266 0.0017207 5.779e-03
## sp46 6.932e-04 1.308e-03 0.0028659 0.0153982 -0.0045318 -7.704e-03
## sp47 -8.450e-03 1.723e-02 0.0217930 0.0203840 -0.0023596 1.810e-03
## sp49 3.617e-03 1.374e-02 -0.0265128 -0.0028514 0.0066874 2.136e-03
## sp57 7.730e-03 -1.198e-03 0.0081118 -0.0116638 0.0061271 1.059e-02
## sp60 -1.523e-03 3.989e-02 -0.1164099 0.0569840 0.0187499 -1.448e-02
## sp62 2.635e-02 9.631e-03 -0.0593510 0.0043131 -0.0055058 1.192e-03
## sp63 3.363e-03 -3.012e-03 -0.0140806 0.0090981 0.0027698 -2.884e-03
## sp64 -8.225e-02 -5.059e-02 0.1426126 0.1602118 -0.0764251 -7.845e-02
## sp65 -1.495e-02 9.269e-03 -0.0481994 0.0325261 -0.0071227 -2.062e-02
## sp66 4.728e-02 -2.884e-02 -0.0049747 -0.0380433 -0.0574828 2.011e-02
## sp69 -2.729e-02 -5.686e-03 0.0172671 0.0095026 -0.0277506 -2.452e-02
## sp70 -4.609e-02 -8.460e-03 0.0293968 0.0225702 -0.0430999 -4.083e-02
## sp72 -1.038e-02 2.826e-03 0.0266287 0.0208168 -0.0158767 -9.490e-03
## sp76 -4.791e-02 -8.108e-03 0.1264702 0.0400893 0.0272424 8.617e-02
## sp77 -1.028e-01 5.260e-02 0.0553714 0.1798984 0.0521137 -6.565e-02
## sp82 2.721e-02 -3.106e-02 -0.0057693 -0.0488570 -0.0225347 9.662e-03
## sp83 -1.609e-02 1.450e-03 0.0413694 0.0455127 0.0064190 -4.567e-03
## sp85 -4.227e-02 -1.385e-02 0.0313702 0.0195240 -0.0320483 -2.642e-02
## sp89 -2.965e-02 -1.166e-02 0.0171872 0.0077389 -0.0202890 -1.528e-02
## sp90 -2.932e-03 9.978e-03 -0.0138726 0.0070024 0.0037843 -5.447e-03
## sp91 -1.977e-02 -3.000e-03 0.0088771 0.0047195 -0.0051569 -4.716e-03
## sp92 1.248e-03 6.685e-03 -0.0408678 0.0131092 0.0076911 -1.487e-03
## sp94 5.236e-02 -2.031e-04 -0.1112302 -0.0218747 0.0019195 -1.204e-02
## sp99 7.975e-02 -1.310e-02 -0.0452705 -0.0730167 0.0497297 5.067e-03
## sp101 4.191e-02 4.817e-03 0.0089492 -0.0093338 0.0449460 -7.896e-03
## sp102 -1.348e-01 8.702e-02 -0.0363772 0.1717310 -0.0039252 -6.849e-02
## sp104 -1.393e-01 -3.117e-02 0.0692347 0.0573388 -0.0994526 -9.611e-02
## sp105 9.551e-03 4.394e-02 -0.0966281 0.1268474 0.1157904 -1.588e-02
## sp107 6.039e-03 1.581e-02 -0.0501987 0.0155727 0.0067248 -2.347e-03
## sp113 7.036e-03 -7.450e-03 -0.0198669 0.0132858 -0.0013440 -1.528e-02
## sp115 -4.214e-03 -1.828e-03 0.0156947 0.0080517 0.0051949 7.268e-03
## sp120 -1.738e-02 6.605e-02 -0.0995187 0.0743455 0.0295624 -3.311e-02
## sp121 -1.378e-02 7.879e-03 0.0127186 0.0190530 -0.0096014 -1.512e-02
## sp122 3.050e-03 4.004e-02 -0.1054441 0.0677863 0.0259340 -1.938e-02
## sp123 -2.452e-02 -9.040e-03 0.0248230 0.0295989 -0.0059514 -1.109e-02
## sp124 6.957e-02 -6.107e-02 -0.0420502 -0.1302887 -0.1167005 5.321e-02
## sp125 -3.655e-03 -3.892e-03 0.0165059 0.0095291 0.0014111 1.036e-02
## sp126 7.643e-02 -6.247e-02 -0.0047061 -0.1466355 -0.0427996 1.057e-02
## sp127 -2.668e-04 -4.728e-03 0.0485549 0.0402553 0.0348878 -1.102e-02
## sp130 -2.332e-04 1.624e-02 0.0240602 -0.0225387 0.0163459 4.275e-02
## sp132 -7.787e-03 2.485e-03 0.0125005 0.0063589 -0.0058270 -4.662e-03
## sp135 -2.377e-02 -2.490e-03 0.0055580 0.0101066 -0.0160974 -1.557e-02
## sp138 2.824e-02 -1.107e-02 -0.0006887 -0.0077027 -0.0374071 8.090e-03
## sp139 1.427e-02 1.756e-02 -0.0144559 0.0136940 0.0231884 -2.119e-02
## sp143 8.425e-02 -7.417e-02 0.0943978 -0.2690425 0.0547812 1.303e-01
## sp144 1.241e-02 7.627e-03 0.0112758 -0.0155303 0.0038582 -1.967e-02
## sp145 -8.336e-02 -4.013e-02 0.0232400 -0.0264962 -0.0015838 3.204e-02
## sp149 -1.179e-02 -1.170e-03 0.0435566 0.0221866 0.0152972 2.796e-02
## sp152 -2.362e-02 -9.027e-03 0.0527197 0.0430844 -0.0120155 -3.417e-04
## sp154 -4.924e-03 3.554e-02 -0.0926772 0.0234283 0.0122194 -9.664e-03
## sp155 -1.330e-02 3.043e-02 -0.0240991 0.0168994 0.0097919 -8.451e-03
## sp157 -1.611e-02 -8.328e-03 0.0037769 0.0152821 -0.0113556 -1.396e-02
## sp158 -2.512e-02 4.077e-02 -0.0697893 0.0352337 0.0071524 -2.636e-02
## sp159 -4.098e-03 -6.621e-03 0.0243325 0.0045090 0.0239888 -3.155e-05
## sp160 9.823e-03 6.465e-03 -0.0340060 0.0034619 0.0095613 9.560e-03
## sp161 -3.777e-02 -4.906e-03 0.0180337 0.0112134 -0.0196061 -1.292e-02
## sp162 -1.260e-02 -4.497e-03 0.0590830 0.0133914 0.0828559 1.668e-02
## sp163 1.641e-03 4.720e-04 -0.0172189 0.0135983 0.0068106 -4.617e-04
## sp167 -8.147e-04 1.937e-03 0.0114451 0.0116773 -0.0049388 -4.558e-03
## sp170 3.349e-02 2.503e-02 0.0565526 0.0398424 0.0757502 4.217e-02
## sp171 -4.557e-02 -1.658e-02 0.1148618 0.0647487 0.0086321 2.845e-02
## sp172 -1.734e-02 -5.698e-03 0.0044496 -0.0006063 -0.0125009 -9.527e-03
## sp173 2.662e-02 1.200e-01 -0.2604359 0.1148390 0.0845442 7.234e-02
## sp175 -5.110e-03 -8.065e-04 -0.0038201 0.0041224 0.0027517 -6.112e-03
## sp177 -3.387e-02 9.061e-03 0.0454185 0.0061442 -0.0003440 2.020e-02
## sp178 -5.248e-02 -3.018e-02 0.0725007 0.0556894 -0.0276696 -1.721e-02
## sp181 -1.043e-01 -4.165e-02 0.2533468 0.1214876 0.1071242 1.173e-01
## sp190 -3.138e-03 2.128e-02 0.0427738 -0.0143858 0.0250350 4.294e-02
## sp191 2.309e-02 -1.549e-03 -0.0395263 0.0201505 0.0046241 3.435e-03
## sp197 1.913e-02 4.995e-03 -0.0079389 -0.0110264 -0.0059066 -2.916e-03
## sp198 3.127e-02 2.104e-03 -0.0141098 -0.0038609 -0.0080426 7.098e-03
## sp199 8.057e-03 9.778e-03 -0.0890363 0.0411820 0.0272429 -2.002e-02
## sp200 3.968e-01 -8.837e-02 0.1761923 -0.4772360 0.3785518 -4.939e-01
## sp203 5.616e-02 1.127e-02 0.0321722 -0.0459800 -0.0326290 -5.757e-02
## sp204 5.926e-02 -4.257e-02 0.0256982 -0.1061739 -0.0473790 5.791e-02
## sp205 -3.497e-02 1.288e-03 0.0499476 0.0250543 -0.0117370 1.924e-03
## sp207 -6.774e-03 5.156e-03 0.0083610 0.0064935 0.0016104 -6.100e-03
## sp211 1.022e-02 2.353e-02 -0.0290848 0.0316872 0.0136976 -1.032e-02
## sp215 9.968e-03 4.832e-03 -0.0114049 -0.0213684 -0.0073901 2.621e-02
## sp219 -7.071e-03 3.058e-03 0.0248603 0.0151305 0.0044547 1.219e-02
## sp225 -1.954e-02 -8.031e-03 0.0273453 0.0249013 -0.0095831 -7.461e-03
## sp227 -1.335e-02 -7.300e-03 0.0084736 0.0118843 -0.0049961 -1.054e-02
## sp231 -4.163e-03 -4.920e-03 0.0146535 0.0196706 -0.0026268 -3.442e-03
## sp236 -2.927e-03 -5.139e-03 0.0191949 0.0089851 0.0104735 9.411e-03
## sp237 1.586e-02 -1.738e-05 0.0093931 -0.0187445 0.0239445 -3.625e-02
## sp239 -1.611e-02 -8.873e-04 0.0260091 -0.0023126 0.0207705 3.938e-02
## sp244 -8.388e-03 2.448e-03 0.0089235 0.0163684 -0.0131271 -1.570e-02
## sp254 1.080e-02 -1.496e-03 0.0007279 -0.0300283 -0.0159786 1.634e-02
## sp257 2.356e-02 -1.019e-02 -0.0136654 -0.0112130 -0.0121773 7.551e-03
## sp259 4.047e-03 1.203e-03 -0.0210097 0.0252724 0.0020735 -1.114e-02
## sp267 2.329e-02 1.187e-03 -0.0472558 0.0115111 -0.0125549 2.986e-03
## sp269 2.363e-03 8.982e-03 0.0100729 0.0064966 0.0128138 -1.685e-02
## sp270 -1.917e-01 -6.959e-02 0.1345488 0.1713901 -0.1762633 -1.879e-01
## sp275 -5.698e-02 -1.998e-02 0.0033344 0.0248993 -0.0290711 -3.769e-02
## sp282 -2.062e-02 1.321e-02 -0.0179313 0.0423063 0.0073471 -6.238e-03
## sp284 -5.880e-02 -8.153e-03 0.0316648 0.0105475 -0.0433014 -3.213e-02
## sp286 4.216e-03 4.974e-03 0.0065479 -0.0226034 -0.0053364 2.203e-02
## sp290 -1.394e-01 -5.141e-02 0.1158637 0.0711050 -0.0365692 -1.506e-02
## sp291 -1.902e-02 2.134e-03 0.0077427 0.0084876 -0.0082827 -9.454e-03
## sp292 -6.736e-02 -5.398e-03 0.1756013 0.0711999 0.0521655 1.126e-01
## sp300 -8.379e-02 -3.976e-02 0.0479917 0.0323440 -0.0443209 -3.240e-02
## sp301 1.915e-02 2.284e-02 -0.0739373 0.0198076 -0.0168024 1.549e-02
## sp303 1.032e-02 4.225e-03 -0.0124977 0.0149959 0.0171795 -1.851e-02
## sp304 -1.893e-01 -9.467e-02 0.1307335 0.1409032 -0.1843802 -1.745e-01
## sp305 -9.192e-02 -2.370e-02 0.0670465 0.0390914 -0.0692509 -5.115e-02
## sp306 -4.146e-03 7.520e-03 -0.0467488 0.0232742 0.0115289 -1.484e-02
## sp307 -3.836e-02 9.548e-02 -0.1084397 0.0294632 0.0333436 6.747e-03
## sp311 2.612e-03 -3.107e-02 -0.0135566 -0.0510482 -0.0104330 3.269e-02
## sp312 1.466e-03 -2.307e-02 0.0382268 -0.0922435 -0.0091245 8.180e-02
## sp313 5.983e-03 -1.575e-02 0.0139103 -0.0412509 0.0056544 3.300e-02
## sp314 1.017e-01 -9.010e-02 0.0262254 -0.1669363 0.1170158 2.212e-02
## sp317 -4.949e-03 -3.245e-02 0.0997235 -0.1019180 0.0106878 1.218e-01
## sp319 3.424e-03 3.834e-03 -0.0010371 0.0083374 -0.0040252 -8.607e-03
## sp321 1.119e-02 -1.553e-02 0.0039708 -0.0373359 0.0026619 -1.487e-02
## sp322 -3.762e-02 3.057e-02 -0.0166560 0.0530376 -0.0017790 -4.716e-02
## sp324 -2.498e-02 -1.295e-02 0.0810174 0.0641784 -0.0004948 1.049e-02
## sp327 6.521e-03 2.780e-02 -0.0537233 0.0171247 0.0078771 5.856e-03
## sp328 1.533e-02 -1.096e-02 -0.0214344 -0.0010567 0.0211069 -2.771e-02
## sp329 1.178e-02 7.805e-03 -0.0211210 0.0015762 0.0058724 2.473e-03
## sp331 1.236e-02 1.019e-02 0.0179220 -0.0145217 0.0142717 1.524e-02
## sp332 -3.788e-03 -1.939e-02 -0.0016457 -0.0487492 0.0231274 2.166e-02
## sp336 3.716e-03 6.386e-03 0.0104944 -0.0015348 0.0045438 1.154e-02
## sp337 5.040e-02 -2.500e-02 0.0493168 -0.1050630 0.1194281 1.902e-02
## sp338 -9.489e-03 -1.423e-02 0.0418124 -0.0071158 0.0443827 4.589e-02
## sp339 -6.655e-03 -9.889e-03 0.0095929 -0.0020520 0.0067747 6.323e-03
## sp347 1.391e-01 -2.319e-02 -0.0559603 -0.1349995 -0.2493490 1.353e-01
## sp349 -1.073e-02 1.581e-02 -0.0125790 0.0317180 -0.0083136 -1.623e-02
## sp351 3.206e-02 1.420e-01 -0.3440935 0.0984005 0.0217686 6.985e-02
## sp354 5.295e-03 6.024e-03 -0.0012590 -0.0086481 0.0082248 8.584e-03
## sp357 -1.735e-02 5.683e-04 0.0057695 0.0024906 -0.0005725 -1.845e-03
## sp359 1.633e-02 3.125e-02 -0.1345692 0.0663376 0.0131934 9.342e-03
## sp360 -2.940e-02 8.627e-02 -0.2267066 0.1317473 0.0412271 -4.416e-02
## sp361 -2.584e-02 1.153e-02 -0.0096620 0.0098482 -0.0066306 -2.053e-02
## sp363 -4.211e-02 -2.532e-03 0.0391872 0.0408476 -0.0386852 -4.102e-02
## sp371 -1.463e-02 8.021e-04 0.0108631 0.0054233 -0.0178911 -1.549e-02
## sp374 -2.846e-02 -1.704e-02 0.0198846 0.0299378 -0.0278468 -3.223e-02
## sp377 1.536e-01 -5.373e-05 -0.2001607 -0.1383674 0.0254316 6.946e-02
## sp378 -1.360e-01 -3.001e-02 0.0861828 0.0243511 -0.0207616 1.943e-02
## sp384 -3.710e-02 -2.028e-02 0.0666670 0.0369641 0.0057056 2.175e-02
## sp388 -3.258e-02 -1.302e-02 0.0589970 0.0485591 -0.0101263 -3.975e-02
## sp390 -3.062e-02 -1.845e-02 0.0204270 0.0139658 -0.0224875 -1.535e-02
## sp391 -8.583e-02 -4.571e-02 0.0626203 0.0313281 -0.0447703 -2.119e-02
## sp395 -1.538e-01 -5.065e-02 0.0718567 0.0160243 -0.0859612 -5.307e-02
## sp400 -7.871e-03 2.810e-03 0.0059807 -0.0033019 -0.0011560 5.520e-03
## sp408 8.079e-02 5.046e-03 0.0102615 -0.1274910 -0.0326935 -9.795e-03
## sp421 8.497e-03 3.375e-02 -0.1674434 0.0550342 0.0178839 -1.133e-02
## sp424 8.862e-03 -7.811e-03 0.0036620 -0.0230116 -0.0100879 1.226e-02
## sp428 1.677e-02 -1.358e-04 0.0061150 -0.0213793 -0.0228843 -1.353e-03
## sp429 2.140e-02 -2.029e-02 -0.0012362 -0.0266216 -0.0044519 -5.972e-03
## sp433 1.743e-02 -1.362e-02 0.0057577 -0.0173611 0.0214730 -6.764e-03
## sp434 1.535e-02 4.633e-03 0.0351585 -0.0044423 0.0177473 -1.674e-02
## sp435 -5.608e-03 -5.295e-03 0.0171144 -0.0108234 0.0160581 1.904e-02
## sp439 -4.964e-03 -5.546e-03 0.0034209 -0.0065703 0.0051569 1.076e-02
## sp442 -2.172e-02 -3.850e-03 0.0139454 0.0055980 -0.0063164 -8.848e-03
## sp445 -6.167e-02 -2.029e-02 0.0458563 -0.0063618 -0.0020619 3.428e-02
## sp447 -1.987e-02 -1.977e-03 0.0079898 -0.0056407 -0.0030798 2.795e-03
## sp449 -1.323e-02 -7.116e-03 0.0156654 0.0087001 -0.0044450 2.362e-03
## sp450 3.807e-02 -5.605e-02 0.0219335 -0.1043530 0.0024984 2.153e-02
## sp451 1.285e-02 1.177e-02 0.0006300 -0.0105977 0.0100332 -1.158e-02
## sp452 -9.093e-03 3.505e-03 0.0491640 -0.0069324 0.0560793 5.615e-02
## sp457 2.182e-02 6.531e-03 -0.0133003 -0.0395658 -0.0323316 1.535e-02
## sp459 1.788e-02 1.584e-02 0.0003771 -0.0509087 -0.0381392 3.682e-02
## sp461 -9.489e-02 -2.887e-02 0.0727811 0.0400430 -0.0426231 -2.336e-02
## sp462 -1.920e-02 1.133e-02 0.0481196 -0.0240767 0.0242828 6.292e-02
## sp464 4.808e-03 4.652e-04 0.0207137 -0.0201656 0.0179882 2.105e-02
## sp468 -4.974e-02 -4.247e-04 0.0949100 -0.0201458 0.0554912 7.299e-02
## sp470 -3.119e-02 -4.286e-03 0.0350138 0.0285412 -0.0282074 -2.566e-02
## sp471 1.563e-02 -4.515e-03 0.0292665 0.0114901 0.0388909 -8.547e-03
## sp473 3.673e-02 -2.495e-02 -0.0183600 -0.0308220 -0.0594649 2.575e-02
## sp474 1.611e-02 -6.003e-03 -0.0416100 0.0118828 0.0015542 -4.461e-03
## sp476 -5.013e-03 6.736e-03 0.0124960 0.0013145 0.0054119 -8.164e-03
## sp479 3.186e-03 4.412e-02 -0.1222582 0.0868513 0.0143900 -1.932e-02
## sp481 -1.924e-02 -6.348e-03 0.0271852 -0.0071829 0.0262771 3.072e-02
## sp482 3.591e-02 -1.883e-02 0.0357469 -0.1233495 -0.0190863 4.694e-02
## sp483 1.611e-02 2.333e-02 0.0340088 -0.0601663 0.0252189 2.188e-02
## sp487 -1.051e-02 -2.346e-03 0.0196271 -0.0011775 0.0075586 2.614e-02
## sp491 -9.643e-03 4.658e-03 0.0229057 0.0048805 0.0107315 1.229e-02
## sp492 -2.444e-02 4.144e-02 0.0425153 0.0338960 0.0638029 6.416e-02
## sp494 -4.109e-03 -9.030e-03 0.0037367 -0.0011260 0.0009321 -4.520e-03
## sp496 1.988e-02 -9.892e-03 -0.0004535 -0.0198522 0.0262693 4.644e-03
## sp497 4.028e-02 1.184e-01 -0.2117258 0.0759246 0.0920845 7.000e-02
## sp498 -1.770e-02 -7.331e-03 0.0062294 0.0171883 -0.0192585 -2.358e-02
## sp499 7.311e-02 1.484e-01 -0.4236318 0.1838782 0.1122623 -4.681e-02
## sp500 -4.058e-02 -1.785e-02 0.0145231 0.0467115 -0.0454493 -5.774e-02
## sp501 -1.064e-01 -2.779e-02 0.1168973 0.0736217 -0.0407355 -1.686e-02
## sp502 -1.461e-02 6.532e-03 0.0124055 0.0064141 0.0076566 2.780e-03
## sp505 -4.580e-02 1.612e-02 -0.0117640 0.0475020 0.0261176 1.198e-02
## sp506 -7.129e-03 -7.046e-03 0.0131444 -0.0081485 0.0194638 2.045e-02
## sp507 -7.248e-02 -2.204e-02 0.0414120 -0.0013934 -0.0247832 2.854e-03
## sp508 -7.194e-03 -2.018e-03 0.0299555 -0.0109482 0.0204890 3.516e-02
## sp509 -4.730e-02 -1.915e-02 0.1226022 0.0892366 0.0127264 3.383e-02
## sp510 -5.395e-03 -1.128e-02 0.0723315 0.0002082 0.0478719 6.956e-02
## sp512 9.306e-03 1.615e-02 -0.0683512 0.0129067 0.0296730 -1.120e-03
## sp514 -4.173e-02 -1.153e-02 0.1192875 0.0641251 0.0312337 4.864e-02
## sp516 -1.441e-02 -2.113e-04 0.0392849 0.0123292 0.0203104 2.226e-02
## sp517 3.055e-03 7.958e-04 0.0136092 -0.0043692 0.0117399 2.102e-03
## sp522 -1.549e-02 -5.687e-03 0.0212370 0.0239061 -0.0202678 -2.055e-02
## sp524 -1.851e-02 -3.794e-03 0.0649385 0.0426548 0.0188739 2.012e-02
## sp526 -2.099e-02 -8.714e-04 0.0965473 0.0337138 0.0702361 5.158e-02
## sp527 5.966e-02 -1.054e-01 0.0017695 -0.1250618 0.0217958 5.135e-02
## sp528 1.157e-01 1.260e-01 -0.3332122 0.0788545 0.0621904 1.118e-01
## sp529 1.018e-01 -2.380e-02 -0.0384002 -0.1508507 -0.0801200 3.949e-02
## sp530 -5.062e-02 -3.037e-02 0.0326687 -0.0056852 0.0132326 2.671e-02
## sp531 -1.994e-02 -8.401e-03 0.0447884 0.0374715 0.0092673 7.222e-03
## sp533 -1.657e-02 -8.974e-03 0.0192117 -0.0048421 0.0184896 2.818e-02
## sp536 -2.586e-02 -4.121e-03 0.0499868 0.0178326 0.0093800 3.082e-02
## sp538 -1.098e-02 -3.341e-03 0.0102928 -0.0023507 0.0062365 1.426e-02
## sp539 1.202e-03 2.242e-03 0.0068623 0.0025080 0.0024156 6.933e-03
## sp540 -2.209e-02 1.710e-03 0.0331732 -0.0061819 0.0189767 5.014e-02
## sp541 -1.603e-02 3.608e-03 0.0026880 0.0040677 -0.0104433 -1.509e-02
## sp542 8.297e-03 4.572e-02 -0.1345839 0.0307636 0.0058982 8.224e-03
## sp546 -1.130e-01 -2.796e-02 0.0740234 -0.0022421 -0.0321804 1.513e-02
## sp547 6.605e-04 3.002e-03 0.0102934 -0.0118132 0.0069632 -2.796e-03
## sp548 -1.821e-03 1.594e-03 0.0202373 -0.0029186 0.0059714 2.242e-02
## sp552 7.913e-03 4.914e-03 0.0451527 0.0078443 0.0297762 2.647e-02
## sp553 5.704e-04 3.221e-02 -0.0768786 0.0687492 0.0068561 -1.909e-02
## sp558 9.819e-03 1.239e-02 -0.0604999 0.0396292 0.0182300 -7.127e-03
## sp559 2.459e-03 2.163e-02 -0.0949329 0.0542991 0.0195456 -2.010e-02
## sp560 -1.572e-02 1.107e-03 0.0289169 -0.0177824 0.0215833 4.116e-02
## sp568 -3.781e-02 1.355e-02 0.0016913 0.0123829 -0.0273171 -3.195e-02
## sp569 -7.580e-03 -3.809e-03 -0.0028869 0.0228153 -0.0082180 -1.612e-02
## sp570 3.500e-03 5.293e-03 0.0178337 0.0034387 0.0075102 1.446e-02
## sp571 7.812e-02 -4.952e-03 0.0697404 -0.0276681 0.0648190 5.344e-02
## sp572 -5.492e-02 8.358e-03 0.1381047 0.0307439 0.0408151 4.570e-02
## sp573 -9.703e-03 -4.250e-03 0.0095432 -0.0064165 0.0150824 1.756e-02
## sp574 7.671e-03 8.830e-03 0.0510478 -0.0225276 0.0352818 6.090e-02
## sp575 -2.272e-02 8.560e-03 -0.0256572 0.0433403 -0.0069356 -3.331e-02
## sp576 -2.763e-02 -1.616e-02 0.0133091 0.0188948 -0.0265801 -2.695e-02
## sp580 6.569e-04 3.137e-03 0.0226353 0.0036504 0.0180041 1.993e-02
## sp583 8.594e-02 -3.637e-02 0.0154677 -0.0370850 -0.1281982 4.912e-02
## sp584 -2.599e-02 -1.082e-02 0.0310429 -0.0072316 0.0160547 3.769e-02
## sp585 -5.581e-02 -1.216e-02 0.0744969 0.0319016 0.0164804 3.308e-02
## sp586 -2.391e-02 -9.100e-03 0.0067079 -0.0005227 -0.0136699 -1.055e-02
## sp590 -2.130e-02 -8.165e-04 0.0080767 0.0035210 -0.0092982 -9.753e-03
## sp592 -9.753e-02 -1.324e-02 0.0594322 0.0172604 -0.0633452 -4.067e-02
## sp593 1.165e-01 -4.580e-02 -0.1110821 -0.1022598 0.0647459 2.923e-02
## sp594 -3.015e-02 -5.452e-03 0.0254405 0.0314367 -0.0191479 -1.987e-02
## sp596 -1.744e-02 -2.400e-03 0.0090418 -0.0067415 -0.0057310 6.337e-03
## sp600 -3.657e-02 -1.192e-02 0.0095782 -0.0048377 -0.0199527 -1.455e-02
## sp604 -7.372e-02 1.123e-02 0.2588139 0.0998033 0.1051835 1.757e-01
## sp605 -2.425e-02 -1.349e-02 0.0098762 0.0093588 -0.0178037 -1.656e-02
## sp608 1.915e-02 -1.169e-02 -0.0060660 -0.0133524 -0.0346470 1.476e-02
## sp609 2.277e-03 1.715e-02 -0.0216785 0.0070423 -0.0011650 4.163e-03
## sp612 -1.222e-02 2.743e-02 -0.0252774 0.0053254 0.0071969 -4.608e-03
## sp617 -4.919e-04 1.144e-02 0.0189376 -0.0116350 0.0087702 4.785e-02
## sp622 3.534e-03 2.247e-02 -0.0408836 0.0123050 0.0071531 -8.027e-03
## sp626 8.295e-02 1.780e-02 -0.0436795 -0.0215458 0.0317852 -3.758e-02
## sp630 -3.112e-02 -1.140e-02 0.0507816 0.0251569 0.0103138 2.744e-02
## sp631 -2.535e-02 9.317e-03 0.0301573 0.0127369 0.0016182 1.107e-02
## sp632 1.765e-02 -1.507e-02 -0.0015959 -0.0078275 -0.0391819 1.579e-02
## sp634 2.667e-02 1.379e-04 -0.0233747 -0.0333707 -0.0428102 2.515e-02
## sp635 -2.919e-02 -2.926e-03 0.0109408 0.0051784 -0.0241260 -2.430e-02
## sp636 3.193e-02 -1.747e-02 -0.0052446 -0.0213706 -0.0398983 1.089e-02
## sp637 2.219e-02 1.238e-02 -0.0840454 0.0272892 0.0083489 -5.052e-03
## sp638 7.644e-02 -1.024e-02 -0.1475279 -0.0821192 -0.0679308 8.214e-02
## sp641 -6.238e-03 -4.222e-03 0.0157197 0.0282342 -0.0005590 -9.369e-03
## sp645 -3.879e-03 1.575e-02 0.0021588 0.0386154 0.0079904 -3.614e-03
## sp648 1.437e-03 -7.561e-03 -0.0023890 -0.0173164 0.0087725 -3.998e-03
## sp650 -1.223e-01 -5.171e-02 0.0718966 0.0320998 -0.0873432 -7.002e-02
## sp651 2.281e-02 -8.244e-03 0.0349708 -0.0207200 0.0633076 9.603e-03
## sp653 -7.257e-03 3.527e-03 0.0058662 0.0082799 -0.0036882 -6.514e-03
## sp654 9.071e-03 1.860e-02 0.0035146 0.0260046 0.0065306 3.957e-03
## sp658 5.507e-03 2.806e-02 -0.0524495 0.0062980 0.0337045 -2.160e-02
## sp660 -6.629e-03 1.337e-02 0.0243968 0.0133246 0.0062573 -8.800e-03
## sp662 -1.957e-04 1.039e-02 0.0166793 0.0089694 0.0008180 2.506e-03
## sp664 2.703e-02 1.959e-02 -0.0416173 0.0717099 0.0249947 -2.115e-02
## sp667 -3.172e-02 2.335e-02 -0.0024533 0.0599964 0.0102320 -3.898e-02
## sp668 5.301e-03 1.617e-02 -0.0483746 0.0107228 0.0025534 -7.368e-03
## sp670 5.432e-03 2.277e-03 0.0123985 -0.0007440 0.0056244 2.237e-03
## sp672 9.180e-02 -2.770e-02 -0.1010607 -0.0586421 -0.0124757 1.373e-02
## sp673 -1.019e-01 1.174e-01 -0.1681256 0.0246112 0.0807389 -2.180e-03
## sp681 -6.306e-03 -5.309e-03 0.0156907 -0.0068691 0.0202413 2.417e-02
## sp684 -7.070e-04 5.310e-04 0.0316359 -0.0268986 0.0374186 3.812e-02
## sp685 2.260e-02 -6.254e-03 0.0040393 -0.0162297 0.0039022 -3.901e-03
## sp687 3.290e-02 -1.965e-02 -0.0071184 -0.0311318 -0.0599763 2.338e-02
## sp689 -2.171e-02 2.549e-02 0.0046369 0.0084299 -0.0086955 -1.217e-02
## sp691 -1.316e-01 -4.174e-02 0.0441031 -0.0061914 -0.0808993 -5.427e-02
## sp695 -3.959e-02 -4.114e-03 0.0095302 0.0378982 -0.0139613 -3.182e-02
## sp699 -1.757e-02 -1.850e-02 0.0067290 -0.0011929 0.0006554 5.492e-03
## sp701 -2.647e-02 -1.267e-02 0.0140340 -0.0032749 0.0035193 5.266e-04
## sp702 -4.062e-04 4.196e-03 0.0178612 -0.0034634 0.0224323 1.562e-02
## sp705 -2.127e-02 2.216e-02 -0.0131339 0.0550652 0.0106261 -1.861e-02
## sp709 -2.421e-02 6.656e-02 -0.1267175 0.0596873 0.0337025 -3.836e-02
## sp711 -3.838e-02 -1.240e-02 0.0126627 0.0111937 -0.0214705 -2.652e-02
## sp714 -1.587e-02 3.768e-02 -0.0588925 0.0251158 0.0176396 -1.640e-02
## sp715 -3.953e-02 -1.107e-02 0.0257038 0.0040505 -0.0222759 -7.187e-03
## sp717 1.844e-03 1.382e-02 -0.0314130 0.0146962 0.0018249 -1.034e-03
## sp718 -6.183e-03 1.966e-03 0.0023997 0.0066404 -0.0005537 -2.811e-03
## sp720 1.062e-01 -2.003e-02 0.1205714 -0.1214363 0.1236290 4.167e-02
## sp721 -1.878e-05 2.305e-03 0.0223480 0.0131141 0.0010157 3.871e-03
## sp724 -3.879e-03 -1.295e-03 0.0352217 0.0093903 0.0216630 -2.782e-02
## sp725 -8.783e-02 -2.877e-02 0.0500131 0.0510435 -0.0920072 -9.214e-02
## sp726 1.318e-02 3.043e-02 -0.0272693 -0.0088374 0.0142886 3.200e-02
## sp728 3.214e-02 4.318e-03 0.0268470 -0.0410778 0.0312560 -5.851e-02
## sp729 -1.149e-03 -3.596e-02 0.0255198 -0.0353695 0.0437973 2.560e-02
## sp730 -6.369e-02 -2.795e-02 0.0368378 0.0135575 -0.0417545 -2.430e-02
## sp733 3.123e-02 2.269e-03 -0.0252242 -0.0219600 -0.0166660 2.782e-03
## sp735 5.718e-03 1.863e-02 -0.0341321 0.0021627 -0.0070889 6.175e-03
## sp736 1.563e-02 -7.998e-03 -0.0098863 -0.0255637 -0.0293462 1.840e-02
## sp737 -8.051e-04 1.544e-02 -0.0176864 0.0061971 0.0023038 -7.611e-03
## sp738 4.449e-02 3.382e-02 -0.0617951 -0.0416594 -0.0574692 5.029e-02
## sp739 -2.065e-02 4.141e-03 0.0217148 0.0170060 -0.0077021 -1.027e-02
##
##
## Site scores (weighted sums of species scores)
##
## RDA1 RDA2 PC1 PC2 PC3 PC4
## row1 -0.087888 0.552914 -1.994e-01 0.2459549 0.0724544 -0.0650095
## row2 -0.205832 0.311115 -3.274e-01 0.0605065 0.0643049 -0.1837365
## row3 -0.428422 -0.272965 9.119e-02 0.1592684 -0.2725134 -0.3253089
## row4 -0.188997 0.364438 -2.017e-01 0.0756182 -0.0447998 -0.2268124
## row5 -0.289373 -0.003411 7.642e-02 0.0983490 0.0548153 -0.0724957
## row6 -0.339221 -0.075242 -5.307e-02 0.0720461 -0.2339226 -0.3847960
## row7 -0.473740 -0.349380 2.634e-01 0.2855188 -0.2113854 -0.1478747
## row8 -0.498620 -0.433369 1.701e-01 0.1211944 -0.3390356 -0.3426189
## row9 -0.344992 -0.056083 8.843e-02 0.2859688 -0.0545421 -0.2469267
## row10 -0.376811 -0.100626 7.168e-02 0.0074241 -0.1674740 -0.1868189
## row11 -0.456260 -0.269315 1.003e-01 -0.0028427 -0.2423244 -0.1767280
## row12 -0.095137 0.061564 -5.082e-02 -0.2576152 0.0887715 0.2384410
## row13 -0.189542 0.451514 -1.735e-01 0.1408304 0.0235338 -0.1154163
## row14 -0.319898 -0.033892 1.132e-01 0.0985540 -0.0046735 0.0130364
## row15 -0.208927 0.100407 1.297e-02 -0.0625584 -0.0327642 0.0380976
## row16 -0.452689 -0.355251 1.411e-01 0.0757099 -0.3436267 -0.3299325
## row17 -0.300695 -0.178972 1.490e-01 -0.0938493 -0.0894486 0.0626617
## row18 -0.200736 -0.221699 2.291e-01 -0.1150121 0.0853169 -0.0164590
## row19 0.294088 -0.408683 8.836e-02 0.0027313 -0.6993080 0.1726124
## row20 0.307746 -0.471212 1.169e-01 -0.0002992 -0.7310116 0.1905094
## row21 -0.304352 -0.112426 1.542e-01 0.1208635 -0.1071370 -0.0801363
## row22 -0.109665 0.522015 -1.649e-01 0.0494310 0.0185084 -0.0261440
## row23 -0.384123 -0.180387 5.313e-02 -0.3639258 -0.1389354 -0.1266164
## row24 0.517517 -0.524138 5.544e-03 -0.3032055 0.1598692 -0.3810124
## row25 0.336602 -0.475575 -7.177e-02 -0.1433286 -0.5661227 0.2805755
## row26 0.304708 -0.539727 4.257e-03 -0.1964196 -0.3863852 0.2214824
## row27 -0.126545 0.177555 -1.243e-02 0.0129411 -0.0088663 0.0647849
## row28 -0.195365 0.309998 3.973e-02 0.0361072 0.0463888 0.0209462
## row29 -0.400835 -0.027776 1.147e-01 0.2072205 -0.2180879 -0.3096797
## row30 0.298471 0.268857 -2.875e-01 0.0806116 -0.2007819 0.2615908
## row31 0.250674 -0.381831 6.439e-02 0.0329507 -0.5103130 0.0661320
## row32 0.480707 -0.605575 1.934e-01 -0.1197812 -0.3614195 -0.2932898
## row33 -0.346557 -0.239444 2.794e-01 0.1583847 -0.0823948 0.0481413
## row34 -0.161356 -0.254205 3.695e-01 0.0957656 0.2607766 0.2893102
## row35 -0.087153 0.147692 6.389e-02 -0.3148034 0.1034214 0.2156664
## row36 -0.184523 0.425438 -1.581e-01 -0.0952531 -0.0806731 -0.1792159
## row37 -0.274958 -0.079369 1.643e-01 -0.0311765 -0.0092977 0.0346441
## row38 0.290063 -0.535902 -5.978e-02 -0.1463492 -0.5275183 0.2593341
## row39 0.410311 -0.549496 7.248e-02 -0.1229301 -0.3966216 -0.0870104
## row40 0.275348 -0.497751 1.751e-01 0.0767793 -0.6747238 0.0576707
## row41 0.269161 -0.379895 5.191e-02 -0.1571947 -0.5228886 0.0524482
## row42 -0.157884 -0.082727 1.081e-01 0.1275855 0.2623933 0.1758715
## row43 -0.351991 -0.292322 2.414e-01 0.2553624 -0.1141328 -0.0754330
## row44 -0.395161 -0.386192 3.053e-01 0.2497383 -0.1018768 -0.1662097
## row45 -0.245999 0.316374 -6.371e-05 0.0548319 -0.0290353 -0.1627630
## row46 -0.264015 0.326149 4.333e-02 0.0131810 -0.0749684 -0.0186424
## row47 -0.339725 -0.363309 3.000e-01 -0.0478048 -0.0081376 0.2532494
## row48 0.460406 -0.510651 2.853e-01 -0.2574740 -0.0040828 -0.4643795
## row49 0.462701 -0.465446 2.910e-01 -0.2664020 -0.0594818 -0.5114366
## row50 0.451930 -0.381883 9.798e-02 -0.4402519 0.2398949 -0.4375590
## row51 -0.403473 -0.138627 2.850e-01 0.2238746 -0.1962521 -0.1321546
## row52 -0.388061 -0.105661 1.777e-01 0.0386092 -0.1722640 -0.0092999
## row53 -0.103308 -0.276470 2.496e-01 -0.0898869 0.3192269 0.2856908
## row54 -0.368589 -0.286923 2.213e-01 -0.1296680 -0.1588238 -0.1852478
## row55 0.263216 -0.422956 -9.295e-02 -0.1227870 -0.4731731 0.2496078
## row56 0.458313 -0.452132 2.984e-01 -0.2475628 0.0823021 -0.5681046
## row57 0.507927 -0.434156 2.446e-01 -0.2805515 0.1459925 -0.4827923
## row58 0.426098 -0.352732 2.662e-01 -0.1889800 0.0820810 -0.6603390
## row59 0.344388 -0.297748 1.097e-01 -0.4188706 0.0002829 -0.4207097
## row60 -0.389965 -0.380890 3.661e-01 0.2368215 -0.1411109 -0.0761540
## row61 -0.196842 -0.325975 2.639e-01 0.0211679 0.1535612 0.3306185
## row62 -0.452548 -0.362994 2.896e-01 0.0782281 -0.1797895 0.0354592
## row63 -0.479994 -0.399784 2.682e-01 0.2400309 0.0018544 0.0457271
## row64 -0.488443 -0.360938 2.757e-01 -0.0645583 -0.2157796 -0.1065061
## row65 0.427337 -0.494563 -6.490e-02 -0.2550007 -0.4350815 0.1328753
## row66 0.299271 -0.429519 -5.293e-02 -0.1918574 -0.5071834 0.2850913
## row67 0.370427 -0.355371 8.709e-02 -0.0699437 -0.5741294 0.1520383
## row68 0.439468 -0.389399 2.992e-01 -0.2400188 0.1537244 -0.6677396
## row69 0.439395 -0.442540 2.762e-01 -0.2758654 0.2069380 -0.4464346
## row70 0.317662 0.138477 7.864e-02 0.1551699 0.2216037 -0.2439770
## row71 -0.084337 -0.072029 3.052e-01 0.0422674 0.2431409 0.3031854
## row72 0.160568 -0.441605 -7.683e-02 -0.3757153 0.0695379 0.2933237
## row73 0.287271 -0.161189 1.358e-01 -0.0784098 -0.0296264 0.0373616
## row74 0.418003 -0.092164 9.122e-02 -0.0628574 0.3101850 -0.2685636
## row75 0.154739 0.234595 1.177e-02 0.2171241 -0.0102360 -0.2622801
## row76 0.121759 0.228763 -4.174e-02 -0.0393228 0.3311589 0.0656610
## row77 -0.424098 -0.220033 1.685e-01 0.3238089 -0.1114694 -0.0868964
## row78 -0.335208 -0.250506 1.873e-01 0.0636596 0.0398395 0.2652030
## row79 -0.165829 -0.289056 6.296e-02 -0.3366152 0.2528081 0.4003081
## row80 0.362266 -0.169825 -1.236e-02 -0.3215840 -0.1376463 0.1566219
## row81 0.190251 -0.001144 -6.283e-02 -0.1721758 -0.3308382 0.1615099
## row82 0.272757 0.012687 6.920e-02 -0.2300912 0.0114792 0.1950768
## row83 -0.017171 0.166011 1.884e-01 0.1006717 0.2637695 0.1735667
## row84 -0.380427 -0.058361 8.493e-02 0.2954548 0.0348875 -0.0380200
## row85 -0.379243 -0.479967 1.230e-01 0.0095748 -0.1516805 -0.0154332
## row86 -0.218900 -0.287299 1.084e-01 -0.2154676 0.0491990 0.2777874
## row87 -0.391189 -0.407972 1.293e-01 -0.1942573 -0.0951527 0.1510672
## row88 0.383724 -0.259127 2.019e-02 -0.2893902 -0.1736247 0.2172046
## row89 0.385130 -0.369046 3.993e-02 -0.3834043 -0.1242485 -0.1164078
## row90 0.300321 -0.263189 2.184e-02 -0.3642813 -0.2208707 0.1660391
## row91 0.196219 0.021074 1.383e-01 -0.1443087 0.2055776 0.0691493
## row92 -0.057156 -0.007903 2.852e-01 -0.1731822 0.0661943 0.4171313
## row93 0.022277 -0.210910 2.529e-01 -0.3979543 -0.0789296 0.3983251
## row94 0.199856 -0.114210 1.589e-01 -0.4446637 0.1364447 0.2019583
## row95 0.264473 0.199238 -6.599e-02 -0.1034910 0.2308431 -0.2150748
## row96 -0.021851 -0.225141 1.937e-01 -0.1658896 -0.0815346 0.2881529
## row97 0.044819 -0.181915 1.897e-01 -0.0581855 0.4693016 0.2891481
## row98 -0.441830 -0.479584 1.510e-01 0.0910504 -0.1213577 -0.0229867
## row99 -0.310737 -0.350058 3.196e-02 0.0494065 -0.0723954 -0.1146818
## row100 -0.313421 -0.114543 1.546e-03 -0.0114063 0.0325578 0.0970700
## row101 0.347056 0.156355 -1.871e-01 -0.2427486 -0.1938506 0.0867869
## row102 0.290711 0.143813 -1.287e-01 -0.2464242 -0.1154259 0.2058150
## row103 0.409967 -0.475803 5.796e-02 -0.5133568 -0.3947230 0.1287492
## row104 0.329673 -0.003625 -8.144e-02 -0.2255582 -0.0364061 0.0609931
## row105 0.104601 -0.191703 2.664e-01 -0.0642278 0.1678152 0.1007850
## row106 -0.017425 0.553012 -7.734e-02 0.2795930 0.1008711 0.1682890
## row107 -0.004583 0.235283 1.494e-01 0.1391938 0.1585812 0.2763267
## row108 -0.045733 0.054636 1.992e-01 0.0619746 0.1353856 0.2956827
## row109 -0.155161 0.090745 1.975e-01 0.1308851 0.0889723 0.3076270
## row110 -0.022909 0.176063 5.826e-02 -0.0749900 0.2160744 0.3759550
## row111 -0.105434 -0.139576 2.018e-01 0.1060811 0.5115118 0.1889270
## row112 -0.286214 -0.273908 7.656e-02 0.0723294 0.3329659 0.3018640
## row113 -0.233232 -0.509177 4.483e-02 -0.0736938 -0.0264496 0.0329822
## row114 -0.512038 -0.228842 2.118e-02 0.1221380 -0.2471083 -0.3054223
## row115 0.467914 -0.232160 -6.135e-03 -0.2685611 -0.1127829 -0.0246794
## row116 0.350595 0.071782 -1.011e-01 -0.3393897 -0.1266022 0.2793465
## row117 0.370961 -0.202899 7.692e-04 -0.2955510 -0.4013961 0.0802537
## row118 -0.106676 0.584918 -6.188e-02 0.3192560 0.0071071 -0.0411419
## row119 -0.142803 0.192797 1.661e-01 0.2074493 0.0479742 0.1420492
## row120 -0.070287 -0.059961 2.606e-01 0.0793364 0.3079435 0.3965692
## row121 -0.016679 -0.074543 2.882e-01 0.0878590 0.3415024 0.3371633
## row122 -0.006272 -0.262611 1.896e-01 -0.0886232 0.3211023 0.3077577
## row123 -0.285876 -0.313202 2.206e-01 0.2145449 0.2819618 0.1618158
## row124 -0.373779 -0.590069 6.828e-02 -0.0639759 -0.0062442 0.0965411
## row125 -0.277344 -0.377353 1.173e-01 0.0488738 0.4053325 0.2848126
## row126 -0.316018 -0.265986 3.280e-02 -0.2960714 0.0425320 0.1823376
## row127 0.214531 0.448937 -2.109e-01 0.0387296 0.0797806 0.1142340
## row128 0.148585 0.768291 -3.890e-01 0.1852815 0.0308002 0.0652364
## row129 0.462407 -0.325125 -4.620e-02 -0.2597042 -0.2238748 0.0266291
## row130 0.324633 0.054996 3.991e-02 0.0376867 0.1658841 -0.3788983
## row131 0.226165 -0.147519 1.528e-01 -0.2362169 0.3766136 -0.0673063
## row132 0.215777 -0.052879 2.890e-02 -0.2304744 -0.1066203 0.2440576
## row133 -0.190632 -0.014113 2.836e-01 0.3278893 0.1076721 0.1366064
## row134 -0.052324 -0.063934 2.517e-01 0.0725933 0.2549865 0.2539337
## row135 -0.207240 0.235273 -1.994e-03 0.3422338 0.0946410 0.0045908
## row136 -0.268323 -0.108223 1.768e-01 0.2508836 0.0247417 0.0336037
## row137 -0.503737 -0.552431 2.119e-01 0.1161068 -0.1201550 0.0166581
## row138 -0.315000 -0.484774 1.363e-01 -0.0782269 -0.0401237 0.0823503
## row139 -0.500580 -0.407530 1.889e-01 0.2447668 -0.0897872 -0.0859453
## row140 -0.349836 0.097154 -1.650e-01 0.1799253 -0.0428844 -0.1675118
## row141 -0.534460 -0.506129 1.626e-01 0.0476115 -0.0680801 -0.0468448
## row142 -0.003507 0.937139 -2.968e-01 0.2009103 0.0672407 0.0190022
## row143 0.090143 0.850384 -3.498e-01 0.3080192 0.0744872 -0.0101566
## row144 0.230513 0.590580 -3.469e-01 0.2503879 -0.0434273 0.1084810
## row145 0.310962 -0.041155 -1.952e-01 -0.0783739 -0.2290886 0.0785619
## row146 0.424688 -0.503808 -4.163e-02 -0.2787833 -0.4813830 0.2087492
## row147 0.487042 -0.460513 1.830e-01 -0.4708423 0.2386021 -0.4358164
## row148 0.441662 -0.415022 2.027e-01 -0.3230993 0.3508648 -0.5459519
## row149 0.051581 0.611677 -1.931e-01 0.0152889 0.0675947 -0.0402478
## row150 -0.291253 -0.190914 3.967e-01 0.3149206 0.0571322 0.0946976
## row151 -0.448975 -0.356029 3.509e-01 0.3835602 -0.1926320 -0.1231675
## row152 -0.420440 -0.285746 3.107e-01 0.3632518 -0.0655772 -0.0540524
## row153 -0.409404 -0.335187 1.961e-01 0.1237080 0.0196804 0.1401002
## row154 -0.381349 -0.422046 1.842e-01 0.0290298 0.0437505 0.2472907
## row155 -0.212031 -0.280007 1.757e-01 -0.0040751 0.2686190 0.3477797
## row156 -0.541202 -0.413003 1.069e-01 0.1326844 -0.0835722 -0.0635822
## row157 0.400148 -0.008532 -1.789e-01 -0.0838715 -0.0564316 0.0612187
## row158 -0.006858 0.544657 -1.989e-01 0.2450727 -0.0295420 -0.1409518
## row159 0.158884 0.871024 -3.402e-01 0.3313243 0.0501595 0.1136501
## row160 0.179987 0.691502 -3.747e-01 0.2532785 0.0782537 0.0631155
## row161 -0.230240 -0.117699 2.510e-01 0.0924118 0.0031924 0.2404947
## row162 -0.272150 -0.151543 2.734e-01 0.3364537 0.0297155 -0.0247413
## row163 -0.344361 -0.164120 3.556e-01 0.2737691 0.0421197 0.0997056
## row164 -0.283351 -0.215635 3.526e-01 0.1525799 0.1164599 0.3565282
## row165 -0.415351 -0.399318 3.818e-01 0.3059217 -0.0229037 0.1646256
## row166 -0.411029 -0.429875 2.193e-01 0.2118367 0.0463563 0.0386532
## row167 -0.505567 -0.273966 5.394e-02 0.1652846 -0.2118961 -0.2519551
## row168 -0.385613 -0.426103 1.897e-01 0.0024086 -0.1710276 0.0505808
## row169 -0.476492 -0.508321 9.096e-02 0.0836281 -0.2906982 -0.2843692
## row170 -0.535244 -0.276582 8.482e-03 -0.0552587 -0.1592211 -0.2524128
## row171 0.322269 0.062057 -1.941e-01 -0.0155194 -0.3841555 0.2686261
## row172 0.111189 0.655824 -3.573e-01 0.0723228 0.0499608 -0.0541217
## row173 0.127697 0.614658 -2.837e-01 0.3387271 0.0378603 0.0100351
## row174 0.213268 0.299363 -1.815e-01 0.1094090 0.0453654 0.1499412
## row175 0.385400 -0.378083 -1.339e-01 -0.2144204 -0.4421179 0.2840937
## row176 0.535679 -0.449752 1.018e-03 -0.3094155 -0.2124636 -0.0477564
## row177 0.340580 -0.211685 1.537e-01 -0.1561876 0.2322781 -0.5214112
## row178 -0.041174 0.114951 1.456e-01 0.0833258 0.2219230 0.1827649
## row179 -0.121205 0.143978 2.321e-01 0.0937375 0.1520730 0.2197969
## row180 -0.164486 0.187646 1.566e-01 0.2082967 0.1775396 0.1343728
## row181 -0.454015 -0.203524 2.927e-01 0.3528895 -0.1137267 -0.1088691
## row182 -0.089263 -0.270668 3.050e-01 -0.0954796 0.3203395 -0.0360201
## row183 -0.367953 -0.107112 1.948e-01 0.2070932 -0.0537075 -0.0723519
## row184 -0.403513 -0.238042 1.827e-01 0.2869072 -0.1713809 -0.2075530
## row185 -0.415432 -0.314537 2.827e-01 0.2476965 0.0802542 0.1491010
## row186 -0.281202 -0.365935 2.026e-01 0.0388977 0.1252628 0.2899372
## row187 -0.568657 -0.549659 8.991e-02 0.1376177 -0.3414578 -0.3250236
## row188 -0.416101 -0.412078 -3.102e-03 -0.2629571 -0.1126484 -0.0933296
## row189 0.065356 0.691432 -3.081e-01 0.1942889 -0.0173165 -0.0275054
## row190 0.211486 0.175726 4.443e-02 0.1346167 0.3043911 -0.0287904
## row191 0.300912 -0.114823 -1.044e-01 -0.0863590 -0.0517742 0.1763494
## row192 0.326875 0.138068 -2.609e-01 0.0114876 0.1671763 -0.0565297
## row193 0.481501 -0.483632 3.686e-02 -0.3285947 -0.1499981 0.0006761
## row194 0.461302 -0.432121 1.960e-01 -0.3508711 0.3142638 -0.5580728
## row195 -0.229408 -0.198972 4.047e-01 0.2120545 0.1999857 0.2932807
## row196 -0.245706 0.111732 2.954e-01 0.3627929 0.1549010 0.1464276
## row197 -0.417625 -0.295236 2.225e-01 0.2774189 -0.1471488 -0.2085390
## row198 -0.572179 -0.548605 3.017e-01 0.2289939 -0.2714664 -0.1931488
## row199 -0.500983 -0.426687 2.193e-01 0.1392795 -0.2487295 -0.1069861
## row200 -0.576891 -0.458124 2.000e-01 0.2370083 -0.2746196 -0.1997471
## row201 -0.447921 -0.435285 1.173e-01 0.0753300 -0.1375309 -0.0345269
## row202 -0.489490 -0.483277 1.837e-01 0.0973551 -0.1390133 -0.0224541
## row203 -0.392554 -0.370533 -9.958e-03 -0.0623121 -0.0583791 0.0400030
## row204 -0.267883 -0.257588 -3.221e-02 -0.1924770 0.0483007 0.2012441
## row205 -0.417166 -0.301867 4.034e-02 -0.1677094 0.0301824 0.0246950
## row206 0.087821 0.768316 -3.255e-01 0.3357847 0.1527557 -0.0798133
## row207 0.111077 0.487758 -2.872e-01 0.3065937 0.0085620 0.0401336
## row208 0.119536 0.579013 -3.055e-01 0.2528690 -0.0117014 0.1234741
## row209 0.266584 0.347557 -3.235e-01 0.0317167 0.0073626 0.1750597
## row210 0.395466 -0.274586 -1.864e-01 -0.1587695 -0.0873024 0.1139289
## row211 0.439670 0.194024 -1.890e-01 -0.0966456 0.1501080 -0.0865409
## row212 -0.262947 -0.216837 4.159e-01 0.2807754 0.0176540 0.2523696
## row213 -0.212013 0.167387 1.816e-01 0.3064016 0.0493375 -0.0491855
## row214 -0.072849 -0.001103 3.135e-01 0.1671255 0.2693863 0.1373813
## row215 0.030366 0.429534 4.042e-02 0.0656518 0.2973356 0.0933976
## row216 -0.204237 -0.085121 3.007e-01 0.2078541 0.2097290 0.1118215
## row217 -0.256872 -0.084341 2.287e-01 0.3057795 0.2614404 0.0889148
## row218 -0.242351 -0.279908 2.993e-01 0.1329058 0.1908263 0.3793889
## row219 -0.314564 -0.215876 2.298e-01 0.2846758 -0.0733827 -0.1214833
## row220 0.138874 0.585180 -3.513e-01 0.0821753 0.1058997 0.1741406
## row221 0.464493 -0.295341 -1.391e-01 -0.2826699 0.0593396 -0.1225193
## row222 0.452788 -0.537966 5.690e-02 -0.3791809 0.2964554 -0.3135358
## row223 0.493479 -0.516629 6.623e-02 -0.3555447 0.1984113 -0.2862006
## row224 0.377334 -0.611371 -3.193e-02 -0.3027663 -0.0184460 0.1153361
## row225 0.484305 -0.456231 -7.646e-02 -0.3220520 0.2276743 -0.2416541
## row226 0.343983 -0.016126 -9.923e-02 -0.0484564 0.0222574 0.0133875
## row227 0.402360 -0.472843 -7.878e-02 -0.3409779 -0.0980300 0.0471017
## row228 0.411699 -0.718301 -1.983e-02 -0.3161562 -0.0966533 0.0342521
## row229 0.391075 -0.513438 2.202e-01 -0.3211589 0.1110717 -0.3615223
## row230 0.022838 -0.283917 2.321e-01 -0.0107140 -0.0564160 0.2589023
## row231 0.312647 -0.030329 -4.133e-02 -0.2351202 0.0172983 0.2930790
## row232 -0.367803 -0.219932 3.602e-01 0.2961631 -0.0170242 0.0406659
## row233 -0.054548 0.516660 -1.606e-01 0.2306901 0.0034929 -0.1179410
## row234 -0.184858 0.065396 5.257e-02 0.2378998 -0.2134254 -0.2454670
## row235 -0.398952 -0.112022 8.180e-02 0.2404392 -0.3734401 -0.4509179
## row236 -0.351838 -0.262967 6.946e-02 0.1837105 -0.2947638 -0.3728647
## row237 -0.499188 -0.491317 1.987e-01 0.2363586 -0.3784944 -0.3154842
## row238 -0.522165 -0.403640 1.364e-01 0.1992052 -0.2554170 -0.2954722
## row239 -0.494156 -0.371451 2.322e-02 -0.2005968 -0.2350914 -0.2122650
## row240 0.465313 -0.498737 -5.195e-02 -0.3472341 0.3649009 -0.3805603
## row241 0.304377 -0.629836 3.775e-04 -0.1869604 -0.3764637 0.2343314
## row242 0.228493 -0.300561 1.105e-01 -0.2542070 -0.0806626 0.3353486
## row243 0.260708 0.168536 6.661e-02 -0.1135934 0.3354718 -0.2697680
## row244 -0.064397 0.115249 1.562e-01 0.0789870 0.0609880 -0.0722148
## row245 -0.035751 0.367380 -7.030e-02 0.2508112 0.0067282 -0.0834440
## row246 0.241613 -0.069591 9.955e-02 -0.2917922 0.2782775 -0.1150581
## row247 -0.198373 0.247750 8.229e-02 0.2172121 -0.0032390 0.0268655
## row248 -0.192189 -0.034393 4.016e-01 0.3711050 0.0346109 0.0195254
## row249 -0.234663 -0.201207 3.458e-01 0.1772564 -0.0281205 0.1915107
## row250 -0.109037 -0.262245 3.710e-01 0.0371362 0.2279452 0.2566310
## row251 -0.277355 -0.081846 1.270e-01 0.2878169 -0.2537308 -0.2364299
## row252 -0.365481 -0.272859 -6.416e-03 0.1476099 -0.3028266 -0.4040685
## row253 -0.403651 -0.375122 4.827e-03 -0.2837189 -0.1040994 -0.0173185
## row254 0.250879 0.685299 -3.394e-01 0.1910489 -0.1022432 0.1690614
## row255 0.380717 -0.559011 -9.614e-02 -0.2928243 -0.1833784 0.1457162
## row256 0.477518 -0.671578 1.268e-02 -0.5048929 0.0721435 -0.0284919
## row257 0.431970 -0.480859 5.586e-02 -0.3765949 0.3638965 -0.2787072
## row258 0.472448 -0.475021 9.577e-02 -0.3108348 0.4000790 -0.5856795
## row259 0.345234 -0.542229 -5.545e-02 -0.2399073 0.0232505 0.2792371
## row260 0.019635 -0.109313 2.325e-01 0.1885837 0.4461095 0.0689050
## row261 0.286145 0.001190 2.661e-02 -0.2357878 0.1370599 0.1083196
## row262 0.157264 -0.050148 9.619e-02 -0.2421764 0.1657991 0.1523235
## row263 -0.167694 0.179966 3.366e-01 0.4968690 0.0414879 0.1278403
## row264 0.018604 -0.262941 2.897e-01 -0.0588295 0.0441730 0.2906172
## row265 -0.172726 -0.179832 4.230e-01 0.3108215 0.2043793 0.2222606
## row266 -0.146684 -0.220732 4.064e-01 0.1657424 0.2118734 0.3995667
## row267 -0.184130 0.051461 -1.269e-02 0.0755240 -0.2131303 -0.1542831
## row268 -0.464544 -0.430105 3.864e-02 0.0635652 -0.2059852 -0.1778452
## row269 0.334456 0.122479 -2.363e-01 -0.0793891 0.3372462 -0.0823526
## row270 0.300010 0.288190 -2.092e-01 0.0371457 -0.2306531 0.2021167
## row271 0.463216 -0.693212 -2.213e-02 -0.4210582 0.1915996 -0.2974301
## row272 0.289834 0.047257 -6.579e-02 -0.1197772 0.3920739 -0.1473295
## row273 0.236971 0.185735 -2.056e-01 -0.0145286 0.3231762 0.0085640
## row274 -0.122724 0.035319 3.634e-01 0.1894490 0.1571291 0.3858705
## row275 0.312868 -0.355681 9.534e-02 -0.0961660 0.0599228 -0.0050746
## row276 0.436896 -0.289347 6.754e-02 -0.1891631 0.2127665 -0.0706267
## row277 -0.197257 0.042564 2.148e-01 0.2656532 0.1499227 0.2708906
## row278 -0.024520 -0.356235 2.214e-01 -0.1629222 0.1740621 0.2665968
## row279 -0.374963 -0.131414 3.668e-02 0.1260044 -0.3412572 -0.3397830
## row280 -0.433962 -0.399143 2.405e-01 0.2421297 -0.0348974 -0.0646901
## row281 -0.414937 -0.315307 9.500e-04 -0.2483441 -0.0908877 -0.0545655
## row282 0.073620 0.566401 -2.609e-01 0.1413117 -0.0276394 0.0746032
## row283 0.326036 -0.063406 -1.939e-01 -0.1399987 0.3227035 0.0407426
## row284 0.450400 -0.386187 -3.232e-02 -0.3014480 0.2968907 -0.1339014
## row285 0.409059 -0.315027 3.102e-02 -0.1994188 0.4728240 -0.4189173
## row286 0.102003 -0.116135 7.118e-02 0.0428418 0.4161379 -0.1568185
## row287 0.163381 0.330386 -1.359e-01 0.0614933 -0.3183160 0.1436790
## row288 0.162067 0.364922 -1.421e-01 0.1005785 -0.0212307 0.1779792
## row289 0.311698 -0.562463 4.012e-02 -0.2004878 -0.1862329 0.2333113
## row290 0.545370 -0.388260 -7.040e-03 -0.2487479 0.1289742 -0.1970800
## row291 -0.220826 -0.223355 3.144e-01 0.1149012 0.1106649 0.3176376
## row292 -0.576362 -0.406101 2.603e-01 0.2451711 -0.4773575 -0.3618973
## row293 -0.345155 -0.112074 3.050e-02 0.2059198 -0.2308911 -0.2726689
## row294 0.216412 -0.039749 -5.235e-02 -0.0831323 0.3953253 -0.2399519
## row295 0.224760 0.246796 -2.329e-01 -0.0494894 0.2812827 0.1088391
## row296 0.376350 -0.330365 3.358e-02 -0.2116214 0.4508355 -0.4478111
## row297 0.360515 -0.514658 -1.064e-01 -0.3061212 -0.1356819 0.2961795
## row298 0.183154 -0.127063 -4.731e-02 -0.0060133 0.1225089 0.0474685
## row299 0.309684 -0.602251 -2.041e-02 -0.2102351 -0.1873295 0.1878689
## row300 0.002221 -0.257359 2.904e-01 -0.0254133 0.3097264 0.0306645
## row301 -0.229821 0.250276 -2.079e-01 0.1661707 0.0561373 -0.1483181
## row302 -0.346294 -0.129798 -4.730e-02 0.1858056 -0.1573401 -0.3069170
## row303 -0.429572 -0.466923 6.571e-02 0.0855608 -0.3772195 -0.4079961
## row304 -0.432025 -0.412790 1.186e-01 0.1837403 -0.3623480 -0.4054053
## row305 0.243142 0.487258 -2.634e-01 0.0983013 -0.1391350 0.1500190
## row306 0.277550 0.303181 -4.075e-01 -0.0986699 -0.0740658 0.1359118
## row307 0.205874 -0.033683 -5.830e-02 -0.1591823 0.0740195 0.1929199
## row308 0.282665 -0.487950 -1.299e-01 -0.2025632 -0.0310174 0.1598026
## row309 0.358063 -0.457717 -5.384e-02 -0.2049044 -0.0328953 0.0780836
## row310 0.448402 -0.669117 -7.358e-02 -0.3101236 -0.0482173 0.1089654
## row311 0.191807 0.109186 -1.608e-01 0.1024394 0.0421049 0.0792457
## row312 0.163694 -0.216818 1.561e-01 -0.1646128 0.1172527 0.1310563
## row313 -0.364887 -0.282271 1.774e-01 0.2625826 -0.0940482 -0.1190991
## row314 -0.112487 -0.196970 9.787e-02 -0.0036324 0.4498647 0.0873229
## row315 -0.452081 -0.430923 2.297e-02 0.0214539 -0.2146492 -0.2048816
## row316 -0.422043 -0.399812 2.147e-02 -0.1903028 -0.1094555 -0.0428210
## row317 0.058210 -0.167585 -1.377e-01 -0.0431031 0.1916476 0.1300537
## row318 0.377451 -0.745553 -1.886e-02 -0.3354939 -0.1984641 0.1987510
## row319 0.340318 -0.066119 2.674e-02 -0.1794617 0.3296267 -0.2574161
## row320 -0.312205 -0.145171 -1.954e-02 0.2268814 -0.1130847 -0.1667477
## row321 -0.319856 -0.219905 6.351e-02 0.0255926 -0.2244267 -0.1969638
## row322 0.258343 -0.354226 -1.952e-01 -0.2129033 -0.1456806 0.2486883
## row323 0.156818 -0.264530 -4.782e-02 -0.1255414 0.0637406 0.2784200
## row324 0.262805 -0.257919 -1.277e-01 -0.0954488 0.0184955 0.0303453
## row325 0.285879 -0.238477 -2.381e-02 -0.2467764 -0.4918892 0.2709115
## row326 0.373694 -0.153330 -1.116e-01 -0.1217310 -0.3051237 0.2069965
## row327 0.076937 -0.352252 -1.026e-01 -0.3147719 0.3224880 0.1474171
## row328 -0.172176 -0.315725 -7.799e-02 -0.2607538 0.1563539 0.2873606
## row329 -0.402509 -0.330492 7.875e-02 -0.2063914 -0.2566489 -0.1683570
## row330 -0.444965 -0.509698 1.420e-01 0.0775184 -0.3372923 -0.2757186
## row331 -0.390309 -0.331256 1.031e-01 -0.1642943 -0.1917616 -0.0854565
## row332 0.201056 0.712783 -2.912e-01 0.0004458 -0.0480493 0.1573228
## row333 0.235364 0.444576 -2.198e-01 -0.1740281 -0.1625558 0.1973167
## row334 0.194097 0.813934 -3.089e-01 0.0162442 0.0383273 0.0523795
## row335 0.370008 0.014004 -5.883e-02 -0.2866334 -0.2186847 0.2482353
## row336 0.176289 0.790849 -2.682e-01 -0.0927415 -0.1107771 0.1048170
## row337 0.282177 -0.046581 -1.763e-02 -0.3677030 -0.1401633 0.1365972
## row338 0.104768 0.791124 -2.742e-01 0.0155985 -0.0502391 0.0885922
## row339 0.177925 0.741828 -3.257e-01 0.0098588 0.0130005 0.1010411
## row340 0.168846 0.536358 -2.619e-01 -0.0818981 -0.1344905 0.0697959
## row341 0.015373 0.599489 -1.913e-01 0.0122900 0.0222664 -0.0507951
## row342 -0.037471 0.820522 -2.295e-01 -0.0330094 0.1417705 0.0027476
## row343 0.129242 0.835863 -3.312e-01 0.0575309 0.0350474 0.0959181
## row344 0.063452 0.948944 -3.626e-01 0.1577793 0.0659636 -0.0062816
## row345 0.115930 0.740918 -2.379e-01 0.1769067 0.0682505 0.1362739
## row346 0.041618 0.966523 -3.721e-01 0.2168524 0.0663800 0.0242383
## row347 0.152858 0.557601 -2.130e-01 -0.1442756 0.0152198 0.1785453
## row348 0.091983 0.897069 -3.037e-01 0.1040434 0.0844215 0.0732537
## row349 0.185397 0.523614 -2.668e-01 0.0696335 -0.1037720 0.1661616
## row350 -0.040357 0.636292 -1.478e-01 -0.0748502 0.0443362 -0.0109303
## row351 0.051944 0.941574 -3.040e-01 -0.1279295 0.0967059 0.0188710
## row352 -0.012924 1.028466 -3.294e-01 0.1096577 0.1067464 -0.0186936
## row353 0.074833 0.663055 -3.235e-01 0.2887699 0.0590078 -0.0492649
## row354 -0.022631 0.918961 -3.178e-01 -0.0105986 0.0853915 -0.0841154
## row355 -0.089497 0.695271 -1.925e-01 -0.0010086 0.0744513 -0.1554344
## row356 -0.062492 0.745375 -2.217e-01 0.0081904 0.0710458 -0.1372061
## row357 0.030768 0.986813 -3.041e-01 0.2005010 0.1067114 -0.0105744
## row358 -0.013522 0.590460 -1.148e-01 0.1119280 -0.0554695 -0.0476007
## row359 -0.089628 0.546852 -1.013e-01 0.0771389 0.0491685 -0.0848041
## row360 0.045888 0.655904 -2.506e-01 0.1309965 0.0870747 -0.0424291
## row361 0.159386 0.778050 -3.395e-01 0.1276867 0.0870617 0.1128584
## row362 0.264753 0.374727 -2.738e-01 0.1264981 0.0100287 0.0837725
## row363 0.395486 -0.484666 -8.672e-02 -0.2722089 -0.1556110 -0.0117563
## row364 0.431563 -0.436058 -1.203e-01 -0.3281453 -0.1533518 0.0597444
## row365 -0.123196 0.751125 -1.983e-01 -0.1466902 0.0919047 -0.1482148
## row366 -0.088503 0.740558 -2.712e-01 -0.1427093 0.0313154 -0.0660755
## row367 -0.035086 0.929211 -2.739e-01 0.1628264 0.0158522 -0.0548594
## row368 0.039091 0.799125 -2.394e-01 0.0593560 0.0195063 -0.0148273
## row369 0.038819 0.947418 -3.519e-01 0.1731819 0.1189899 0.0471182
## row370 0.029936 0.738078 -3.456e-01 0.1212960 0.0835381 -0.0546197
## row371 0.147079 0.800870 -3.458e-01 0.1646618 0.0512198 0.1289918
## row372 0.086427 0.748476 -3.246e-01 0.3019158 0.0665971 0.0402653
## row373 -0.008898 0.699376 -2.979e-01 0.3054687 -0.0165957 0.0132088
## row374 0.161535 0.784593 -2.904e-01 0.2745163 0.0590707 0.0873284
## row375 0.037191 0.686452 -3.249e-01 -0.0939291 0.0298112 -0.0249595
## row376 -0.051560 0.802347 -2.477e-01 0.0537940 0.0719116 -0.1498246
## row377 -0.032301 0.610872 -1.691e-01 -0.2045047 0.0722768 0.0022554
## row378 -0.089638 0.766262 -2.245e-01 0.0075852 0.0869008 -0.1148197
## row379 0.012713 0.845047 -3.244e-01 0.2862012 0.0372968 -0.0817570
## row380 0.065182 0.787278 -2.559e-01 0.2547517 0.0676635 0.0538997
## row381 0.214354 0.617566 -3.478e-01 0.1307106 0.2069684 0.0657545
## row382 0.015121 0.771543 -3.315e-01 -0.1501093 0.0410792 0.0046638
## row383 0.014026 0.888141 -2.754e-01 -0.0838538 0.0906406 0.0583582
## row384 -0.047079 1.006402 -3.568e-01 -0.0178815 0.1195547 -0.0466450
## row385 0.011227 0.712569 -2.077e-01 0.2537831 0.1138727 -0.0754871
## row386 -0.044380 0.741958 -2.873e-01 0.1474601 0.1125082 -0.1172322
## row387 -0.022989 0.621264 -3.322e-01 0.0549107 0.0981670 -0.1029660
## row388 0.017955 0.877467 -3.229e-01 0.3613070 0.0543069 -0.0953373
## row389 -0.087933 0.811643 -2.950e-01 0.0835657 0.1127128 -0.1087355
## row390 0.123668 0.584640 -2.834e-01 0.0029947 -0.0609547 0.0634503
## row391 -0.033400 0.641726 -2.801e-01 -0.0021596 0.0761178 -0.1371291
## row392 -0.079335 0.504897 -1.916e-01 0.1337777 -0.0224931 -0.1249927
## row393 -0.015114 0.446087 -2.099e-01 0.1843101 -0.0007311 -0.1348903
## row394 0.010950 0.752914 -3.710e-01 0.1061733 0.1262350 -0.0865008
## row395 0.022337 0.746921 -3.636e-01 0.2011268 0.0783115 0.0229193
## row396 0.126560 0.746447 -4.341e-01 0.2209991 0.1803378 0.0527031
## row397 0.298818 -0.281275 -1.014e-01 -0.2410308 0.0487115 0.1717279
## row398 0.285319 -0.483768 -1.287e-01 -0.2663653 -0.2805819 0.2289558
## row399 -0.030474 0.770380 -2.902e-01 0.2278822 0.0439496 -0.1170225
## row400 -0.015638 0.720951 -3.577e-01 0.2457587 0.0841843 -0.1107608
## row401 0.042098 0.513404 -2.499e-01 0.1774877 0.0113380 0.0406807
## row402 -0.029791 0.649282 -2.920e-01 0.2759179 0.0778414 -0.1444217
## row403 0.035928 0.742812 -3.644e-01 0.2202518 0.1093809 -0.0877470
## row404 -0.016595 0.698103 -2.907e-01 0.2313020 0.0812285 -0.0566156
## row405 -0.028801 0.738869 -3.750e-01 0.2737005 0.1355194 -0.0988141
## row406 0.144160 0.651906 -4.005e-01 0.1403306 0.0873335 0.0368998
## row407 0.536817 -0.442951 -2.058e-03 -0.3594957 0.2427913 -0.4385907
## row408 0.454020 -0.397737 1.084e-01 -0.4816169 -0.1007727 -0.0171326
## row409 0.429477 -0.305773 1.551e-01 -0.2084262 0.3474323 -0.3839959
## row410 0.148809 0.656535 -2.825e-01 0.2645575 0.1083676 -0.0211197
## row411 0.168319 -0.374791 7.313e-02 -0.1083193 -0.0573447 0.1477163
## row412 -0.045215 0.115751 1.264e-01 0.2743248 0.2093527 0.1318480
## row413 -0.010723 0.602663 -3.058e-01 0.1354262 0.0461216 -0.1066651
## row414 0.430633 -0.005407 -2.278e-01 -0.1986050 0.2428654 -0.3313752
## row415 0.319463 0.084021 -1.812e-01 -0.0578948 0.2827060 -0.2230556
## row416 -0.388495 -0.383613 7.615e-02 -0.2690893 -0.0401608 0.1061439
## row417 -0.411998 -0.379206 9.710e-02 -0.2074297 -0.1943617 -0.1215599
##
##
## Site constraints (linear combinations of constraining variables)
##
## RDA1 RDA2 PC1 PC2 PC3 PC4
## row1 -0.0127920 1.224e-02 -1.994e-01 0.2459549 0.0724544 -0.0650095
## row2 -0.4068613 -3.100e-01 -3.274e-01 0.0605065 0.0643049 -0.1837365
## row3 -0.1229280 -1.457e-01 9.119e-02 0.1592684 -0.2725134 -0.3253089
## row4 -0.2602638 2.633e-02 -2.017e-01 0.0756182 -0.0447998 -0.2268124
## row5 -0.1999637 -8.675e-02 7.642e-02 0.0983490 0.0548153 -0.0724957
## row6 -0.3006499 -3.491e-02 -5.307e-02 0.0720461 -0.2339226 -0.3847960
## row7 -0.0113889 -1.316e-01 2.634e-01 0.2855188 -0.2113854 -0.1478747
## row8 -0.2229594 -3.326e-02 1.701e-01 0.1211944 -0.3390356 -0.3426189
## row9 -0.1337754 -5.022e-02 8.843e-02 0.2859688 -0.0545421 -0.2469267
## row10 -0.2241371 -4.102e-02 7.168e-02 0.0074241 -0.1674740 -0.1868189
## row11 -0.2258868 -1.617e-02 1.003e-01 -0.0028427 -0.2423244 -0.1767280
## row12 -0.2547203 3.564e-02 -5.082e-02 -0.2576152 0.0887715 0.2384410
## row13 -0.1721291 3.410e-02 -1.735e-01 0.1408304 0.0235338 -0.1154163
## row14 -0.1841137 4.187e-02 1.132e-01 0.0985540 -0.0046735 0.0130364
## row15 -0.1866709 2.969e-02 1.297e-02 -0.0625584 -0.0327642 0.0380976
## row16 -0.1866709 2.969e-02 1.411e-01 0.0757099 -0.3436267 -0.3299325
## row17 -0.1271842 1.519e-01 1.490e-01 -0.0938493 -0.0894486 0.0626617
## row18 -0.1271842 1.519e-01 2.291e-01 -0.1150121 0.0853169 -0.0164590
## row19 0.4530559 -7.523e-02 8.836e-02 0.0027313 -0.6993080 0.1726124
## row20 0.4635987 -2.324e-02 1.169e-01 -0.0002992 -0.7310116 0.1905094
## row21 -0.1271842 1.519e-01 1.542e-01 0.1208635 -0.1071370 -0.0801363
## row22 -0.1788811 1.249e-01 -1.649e-01 0.0494310 0.0185084 -0.0261440
## row23 -0.6199877 3.274e-01 5.313e-02 -0.3639258 -0.1389354 -0.1266164
## row24 0.2358012 -2.532e-01 5.544e-03 -0.3032055 0.1598692 -0.3810124
## row25 0.2063117 -2.710e-01 -7.177e-02 -0.1433286 -0.5661227 0.2805755
## row26 0.2272634 -2.915e-01 4.257e-03 -0.1964196 -0.3863852 0.2214824
## row27 -0.0062150 9.593e-02 -1.243e-02 0.0129411 -0.0088663 0.0647849
## row28 -0.1547090 1.714e-01 3.973e-02 0.0361072 0.0463888 0.0209462
## row29 -0.1547090 1.714e-01 1.147e-01 0.2072205 -0.2180879 -0.3096797
## row30 0.2388211 -2.159e-01 -2.875e-01 0.0806116 -0.2007819 0.2615908
## row31 0.3635458 -2.111e-01 6.439e-02 0.0329507 -0.5103130 0.0661320
## row32 0.5339711 -3.606e-02 1.934e-01 -0.1197812 -0.3614195 -0.2932898
## row33 -0.0098871 9.608e-02 2.794e-01 0.1583847 -0.0823948 0.0481413
## row34 -0.0098871 9.608e-02 3.695e-01 0.0957656 0.2607766 0.2893102
## row35 -0.2927464 4.092e-01 6.389e-02 -0.3148034 0.1034214 0.2156664
## row36 -0.3576918 3.458e-01 -1.581e-01 -0.0952531 -0.0806731 -0.1792159
## row37 -0.2541708 2.716e-01 1.643e-01 -0.0311765 -0.0092977 0.0346441
## row38 0.1491303 -3.431e-01 -5.978e-02 -0.1463492 -0.5275183 0.2593341
## row39 0.3571774 -1.925e-01 7.248e-02 -0.1229301 -0.3966216 -0.0870104
## row40 0.5558333 5.227e-02 1.751e-01 0.0767793 -0.6747238 0.0576707
## row41 0.1650987 -3.190e-02 5.191e-02 -0.1571947 -0.5228886 0.0524482
## row42 -0.1356726 -1.405e-01 1.081e-01 0.1275855 0.2623933 0.1758715
## row43 -0.0645243 3.326e-06 2.414e-01 0.2553624 -0.1141328 -0.0754330
## row44 -0.0645243 3.326e-06 3.053e-01 0.2497383 -0.1018768 -0.1662097
## row45 -0.1791008 3.388e-01 -6.371e-05 0.0548319 -0.0290353 -0.1627630
## row46 -0.0950763 2.504e-01 4.333e-02 0.0131810 -0.0749684 -0.0186424
## row47 -0.1694459 1.984e-01 3.000e-01 -0.0478048 -0.0081376 0.2532494
## row48 0.4848239 1.501e-01 2.853e-01 -0.2574740 -0.0040828 -0.4643795
## row49 0.4557960 2.574e-01 2.910e-01 -0.2664020 -0.0594818 -0.5114366
## row50 0.0580200 2.090e-02 9.798e-02 -0.4402519 0.2398949 -0.4375590
## row51 0.0341750 1.941e-01 2.850e-01 0.2238746 -0.1962521 -0.1321546
## row52 -0.0275299 1.525e-01 1.777e-01 0.0386092 -0.1722640 -0.0092999
## row53 -0.1128926 5.861e-02 2.496e-01 -0.0898869 0.3192269 0.2856908
## row54 -0.3146722 3.851e-01 2.213e-01 -0.1296680 -0.1588238 -0.1852478
## row55 0.1370138 -3.177e-01 -9.295e-02 -0.1227870 -0.4731731 0.2496078
## row56 0.4841320 2.267e-01 2.984e-01 -0.2475628 0.0823021 -0.5681046
## row57 0.4557960 2.574e-01 2.446e-01 -0.2805515 0.1459925 -0.4827923
## row58 0.4502186 1.379e-01 2.662e-01 -0.1889800 0.0820810 -0.6603390
## row59 -0.0526229 1.730e-01 1.097e-01 -0.4188706 0.0002829 -0.4207097
## row60 -0.0526229 1.730e-01 3.661e-01 0.2368215 -0.1411109 -0.0761540
## row61 -0.0139430 -1.060e-02 2.639e-01 0.0211679 0.1535612 0.3306185
## row62 -0.0332967 1.389e-01 2.896e-01 0.0782281 -0.1797895 0.0354592
## row63 -0.2278770 -1.705e-01 2.682e-01 0.2400309 0.0018544 0.0457271
## row64 -0.3339032 3.024e-01 2.757e-01 -0.0645583 -0.2157796 -0.1065061
## row65 0.2018551 -2.035e-01 -6.490e-02 -0.2550007 -0.4350815 0.1328753
## row66 0.1482151 -1.868e-01 -5.293e-02 -0.1918574 -0.5071834 0.2850913
## row67 0.4431394 -7.395e-03 8.709e-02 -0.0699437 -0.5741294 0.1520383
## row68 0.4597668 2.272e-01 2.992e-01 -0.2400188 0.1537244 -0.6677396
## row69 0.4363207 1.093e-01 2.762e-01 -0.2758654 0.2069380 -0.4464346
## row70 0.4574207 9.925e-02 7.864e-02 0.1551699 0.2216037 -0.2439770
## row71 0.0389812 1.533e-01 3.052e-01 0.0422674 0.2431409 0.3031854
## row72 -0.2187624 -3.273e-01 -7.683e-02 -0.3757153 0.0695379 0.2933237
## row73 0.3078756 1.540e-01 1.358e-01 -0.0784098 -0.0296264 0.0373616
## row74 0.4072004 4.297e-02 9.122e-02 -0.0628574 0.3101850 -0.2685636
## row75 0.3719384 1.891e-01 1.177e-02 0.2171241 -0.0102360 -0.2622801
## row76 -0.1389153 -3.534e-02 -4.174e-02 -0.0393228 0.3311589 0.0656610
## row77 -0.0683222 -1.680e-01 1.685e-01 0.3238089 -0.1114694 -0.0868964
## row78 -0.1004149 -1.245e-01 1.873e-01 0.0636596 0.0398395 0.2652030
## row79 -0.5489052 -4.801e-02 6.296e-02 -0.3366152 0.2528081 0.4003081
## row80 0.0932675 2.481e-01 -1.236e-02 -0.3215840 -0.1376463 0.1566219
## row81 0.0178595 2.302e-01 -6.283e-02 -0.1721758 -0.3308382 0.1615099
## row82 0.1204927 2.397e-01 6.920e-02 -0.2300912 0.0114792 0.1950768
## row83 0.0169655 1.992e-01 1.884e-01 0.1006717 0.2637695 0.1735667
## row84 -0.0920350 -2.202e-01 8.493e-02 0.2954548 0.0348875 -0.0380200
## row85 -0.0920350 -2.202e-01 1.230e-01 0.0095748 -0.1516805 -0.0154332
## row86 -0.2938791 3.046e-02 1.084e-01 -0.2154676 0.0491990 0.2777874
## row87 -0.2938791 3.046e-02 1.293e-01 -0.1942573 -0.0951527 0.1510672
## row88 0.2100184 1.667e-01 2.019e-02 -0.2893902 -0.1736247 0.2172046
## row89 0.0393806 1.528e-01 3.993e-02 -0.3834043 -0.1242485 -0.1164078
## row90 0.0337239 2.087e-01 2.184e-02 -0.3642813 -0.2208707 0.1660391
## row91 0.1643925 1.636e-01 1.383e-01 -0.1443087 0.2055776 0.0691493
## row92 -0.0645885 4.383e-01 2.852e-01 -0.1731822 0.0661943 0.4171313
## row93 -0.1216132 5.155e-01 2.529e-01 -0.3979543 -0.0789296 0.3983251
## row94 -0.1216132 5.155e-01 1.589e-01 -0.4446637 0.1364447 0.2019583
## row95 0.0615777 2.025e-01 -6.599e-02 -0.1034910 0.2308431 -0.2150748
## row96 0.0088667 1.620e-01 1.937e-01 -0.1658896 -0.0815346 0.2881529
## row97 -0.0803081 -1.328e-01 1.897e-01 -0.0581855 0.4693016 0.2891481
## row98 -0.1666377 -2.902e-01 1.510e-01 0.0910504 -0.1213577 -0.0229867
## row99 -0.1666377 -2.902e-01 3.196e-02 0.0494065 -0.0723954 -0.1146818
## row100 -0.2395524 -1.943e-01 1.546e-03 -0.0114063 0.0325578 0.0970700
## row101 0.0193690 2.665e-01 -1.871e-01 -0.2427486 -0.1938506 0.0867869
## row102 0.0174988 2.835e-01 -1.287e-01 -0.2464242 -0.1154259 0.2058150
## row103 -0.0053870 1.851e-01 5.796e-02 -0.5133568 -0.3947230 0.1287492
## row104 0.0845538 3.926e-02 -8.144e-02 -0.2255582 -0.0364061 0.0609931
## row105 0.1794889 1.346e-01 2.664e-01 -0.0642278 0.1678152 0.1007850
## row106 0.1109025 5.944e-02 -7.734e-02 0.2795930 0.1008711 0.1682890
## row107 0.0903979 1.504e-01 1.494e-01 0.1391938 0.1585812 0.2763267
## row108 0.1068130 8.855e-02 1.992e-01 0.0619746 0.1353856 0.2956827
## row109 0.1036542 9.955e-02 1.975e-01 0.1308851 0.0889723 0.3076270
## row110 -0.0128492 -8.807e-03 5.826e-02 -0.0749900 0.2160744 0.3759550
## row111 -0.1596901 -2.335e-01 2.018e-01 0.1060811 0.5115118 0.1889270
## row112 -0.3314460 -4.581e-01 7.656e-02 0.0723294 0.3329659 0.3018640
## row113 -0.2040536 -3.347e-01 4.483e-02 -0.0736938 -0.0264496 0.0329822
## row114 -0.3083257 -1.707e-01 2.118e-02 0.1221380 -0.2471083 -0.3054223
## row115 0.1898732 8.980e-02 -6.135e-03 -0.2685611 -0.1127829 -0.0246794
## row116 -0.0001058 2.754e-01 -1.011e-01 -0.3393897 -0.1266022 0.2793465
## row117 0.1427707 2.338e-01 7.692e-04 -0.2955510 -0.4013961 0.0802537
## row118 0.1421268 8.189e-02 -6.188e-02 0.3192560 0.0071071 -0.0411419
## row119 0.1398326 1.118e-01 1.661e-01 0.2074493 0.0479742 0.1420492
## row120 0.0446450 -1.133e-02 2.606e-01 0.0793364 0.3079435 0.3965692
## row121 0.0703757 -2.110e-02 2.882e-01 0.0878590 0.3415024 0.3371633
## row122 -0.0364203 -1.450e-01 1.896e-01 -0.0886232 0.3211023 0.3077577
## row123 -0.1842640 -2.973e-01 2.206e-01 0.2145449 0.2819618 0.1618158
## row124 -0.2985289 -4.354e-01 6.828e-02 -0.0639759 -0.0062442 0.0965411
## row125 -0.3832938 -5.122e-01 1.173e-01 0.0488738 0.4053325 0.2848126
## row126 -0.4766281 -5.737e-02 3.280e-02 -0.2960714 0.0425320 0.1823376
## row127 0.1216303 1.119e-01 -2.109e-01 0.0387296 0.0797806 0.1142340
## row128 0.0876036 4.060e-02 -3.890e-01 0.1852815 0.0308002 0.0652364
## row129 0.2456054 1.236e-02 -4.620e-02 -0.2597042 -0.2238748 0.0266291
## row130 0.3269933 6.947e-02 3.991e-02 0.0376867 0.1658841 -0.3788983
## row131 0.0354844 1.245e-01 1.528e-01 -0.2362169 0.3766136 -0.0673063
## row132 0.0515734 1.825e-01 2.890e-02 -0.2304744 -0.1066203 0.2440576
## row133 0.0961475 6.739e-02 2.836e-01 0.3278893 0.1076721 0.1366064
## row134 0.1074446 4.362e-02 2.517e-01 0.0725933 0.2549865 0.2539337
## row135 -0.0430603 -1.600e-01 -1.994e-03 0.3422338 0.0946410 0.0045908
## row136 -0.0690178 -1.319e-01 1.768e-01 0.2508836 0.0247417 0.0336037
## row137 -0.1964843 -2.475e-01 2.119e-01 0.1161068 -0.1201550 0.0166581
## row138 -0.1911398 -2.587e-01 1.363e-01 -0.0782269 -0.0401237 0.0823503
## row139 -0.1994406 -2.957e-01 1.889e-01 0.2447668 -0.0897872 -0.0859453
## row140 -0.2694781 -3.906e-01 -1.650e-01 0.1799253 -0.0428844 -0.1675118
## row141 -0.4240070 -1.588e-01 1.626e-01 0.0476115 -0.0680801 -0.0468448
## row142 0.0603974 2.351e-01 -2.968e-01 0.2009103 0.0672407 0.0190022
## row143 0.1735871 -1.790e-02 -3.498e-01 0.3080192 0.0744872 -0.0101566
## row144 0.2708833 -1.340e-01 -3.469e-01 0.2503879 -0.0434273 0.1084810
## row145 0.1534961 -2.328e-02 -1.952e-01 -0.0783739 -0.2290886 0.0785619
## row146 0.1892882 -1.099e-01 -4.163e-02 -0.2787833 -0.4813830 0.2087492
## row147 0.1333295 2.299e-01 1.830e-01 -0.4708423 0.2386021 -0.4358164
## row148 0.2260897 7.337e-02 2.027e-01 -0.3230993 0.3508648 -0.5459519
## row149 -0.0270408 1.919e-01 -1.931e-01 0.0152889 0.0675947 -0.0402478
## row150 0.0050176 1.902e-01 3.967e-01 0.3149206 0.0571322 0.0946976
## row151 0.0578401 1.069e-02 3.509e-01 0.3835602 -0.1926320 -0.1231675
## row152 -0.0329802 -5.223e-02 3.107e-01 0.3632518 -0.0655772 -0.0540524
## row153 -0.1710344 -1.558e-01 1.961e-01 0.1237080 0.0196804 0.1401002
## row154 -0.2150844 -2.248e-01 1.842e-01 0.0290298 0.0437505 0.2472907
## row155 -0.2150844 -2.248e-01 1.757e-01 -0.0040751 0.2686190 0.3477797
## row156 -0.2861410 -3.787e-01 1.069e-01 0.1326844 -0.0835722 -0.0635822
## row157 0.2255863 -1.686e-01 -1.789e-01 -0.0838715 -0.0564316 0.0612187
## row158 0.1251539 1.002e-01 -1.989e-01 0.2450727 -0.0295420 -0.1409518
## row159 0.2929960 -4.260e-02 -3.402e-01 0.3313243 0.0501595 0.1136501
## row160 0.2321150 -9.819e-02 -3.747e-01 0.2532785 0.0782537 0.0631155
## row161 0.0684145 6.702e-02 2.510e-01 0.0924118 0.0031924 0.2404947
## row162 0.0084382 5.201e-02 2.734e-01 0.3364537 0.0297155 -0.0247413
## row163 -0.0376139 6.572e-02 3.556e-01 0.2737691 0.0421197 0.0997056
## row164 0.0077485 8.787e-02 3.526e-01 0.1525799 0.1164599 0.3565282
## row165 0.0439417 -4.382e-02 3.818e-01 0.3059217 -0.0229037 0.1646256
## row166 -0.2362726 -3.028e-01 2.193e-01 0.2118367 0.0463563 0.0386532
## row167 -0.2325257 -2.392e-01 5.394e-02 0.1652846 -0.2118961 -0.2519551
## row168 -0.0905585 -9.440e-02 1.897e-01 0.0024086 -0.1710276 0.0505808
## row169 -0.2627517 -2.041e-01 9.096e-02 0.0836281 -0.2906982 -0.2843692
## row170 -0.5564589 -9.380e-02 8.482e-03 -0.0552587 -0.1592211 -0.2524128
## row171 0.2553241 -1.410e-01 -1.941e-01 -0.0155194 -0.3841555 0.2686261
## row172 -0.0245035 1.533e-01 -3.573e-01 0.0723228 0.0499608 -0.0541217
## row173 0.2811262 -5.629e-02 -2.837e-01 0.3387271 0.0378603 0.0100351
## row174 0.2058251 -1.195e-01 -1.815e-01 0.1094090 0.0453654 0.1499412
## row175 0.1659934 -1.812e-01 -1.339e-01 -0.2144204 -0.4421179 0.2840937
## row176 0.2809977 -3.167e-02 1.018e-03 -0.3094155 -0.2124636 -0.0477564
## row177 0.2841331 1.592e-01 1.537e-01 -0.1561876 0.2322781 -0.5214112
## row178 0.0304306 -2.731e-02 1.456e-01 0.0833258 0.2219230 0.1827649
## row179 0.0062296 1.858e-01 2.321e-01 0.0937375 0.1520730 0.2197969
## row180 -0.0511259 6.505e-02 1.566e-01 0.2082967 0.1775396 0.1343728
## row181 -0.0449794 1.645e-02 2.927e-01 0.3528895 -0.1137267 -0.1088691
## row182 -0.1331172 1.569e-01 3.050e-01 -0.0954796 0.3203395 -0.0360201
## row183 -0.0796051 5.918e-02 1.948e-01 0.2070932 -0.0537075 -0.0723519
## row184 -0.0257224 -8.540e-02 1.827e-01 0.2869072 -0.1713809 -0.2075530
## row185 -0.0848816 -2.250e-01 2.827e-01 0.2476965 0.0802542 0.1491010
## row186 -0.1183389 -1.584e-01 2.026e-01 0.0388977 0.1252628 0.2899372
## row187 -0.2801599 -2.929e-01 8.991e-02 0.1376177 -0.3414578 -0.3250236
## row188 -0.5694009 -7.464e-02 -3.102e-03 -0.2629571 -0.1126484 -0.0933296
## row189 0.0662274 -3.481e-03 -3.081e-01 0.1942889 -0.0173165 -0.0275054
## row190 0.2602532 3.432e-02 4.443e-02 0.1346167 0.3043911 -0.0287904
## row191 0.2400239 -4.402e-02 -1.044e-01 -0.0863590 -0.0517742 0.1763494
## row192 0.1493935 -2.456e-01 -2.609e-01 0.0114876 0.1671763 -0.0565297
## row193 0.2824003 -1.513e-01 3.686e-02 -0.3285947 -0.1499981 0.0006761
## row194 0.2442489 6.456e-02 1.960e-01 -0.3508711 0.3142638 -0.5580728
## row195 0.0619920 6.073e-02 4.047e-01 0.2120545 0.1999857 0.2932807
## row196 0.0891841 1.166e-01 2.954e-01 0.3627929 0.1549010 0.1464276
## row197 -0.1254374 -7.041e-03 2.225e-01 0.2774189 -0.1471488 -0.2085390
## row198 -0.1119708 -4.100e-02 3.017e-01 0.2289939 -0.2714664 -0.1931488
## row199 -0.1000223 -8.482e-02 2.193e-01 0.1392795 -0.2487295 -0.1069861
## row200 -0.1075602 -2.138e-01 2.000e-01 0.2370083 -0.2746196 -0.1997471
## row201 -0.2243733 -2.345e-01 1.173e-01 0.0753300 -0.1375309 -0.0345269
## row202 -0.2243733 -2.345e-01 1.837e-01 0.0973551 -0.1390133 -0.0224541
## row203 -0.3047971 -3.888e-01 -9.958e-03 -0.0623121 -0.0583791 0.0400030
## row204 -0.3573369 -3.253e-01 -3.221e-02 -0.1924770 0.0483007 0.2012441
## row205 -0.5588096 -8.523e-02 4.034e-02 -0.1677094 0.0301824 0.0246950
## row206 0.1706309 -1.171e-01 -3.255e-01 0.3357847 0.1527557 -0.0798133
## row207 0.2489711 -1.976e-01 -2.872e-01 0.3065937 0.0085620 0.0401336
## row208 0.2280392 -2.052e-01 -3.055e-01 0.2528690 -0.0117014 0.1234741
## row209 0.1308463 -1.661e-01 -3.235e-01 0.0317167 0.0073626 0.1750597
## row210 0.1692430 -2.523e-01 -1.864e-01 -0.1587695 -0.0873024 0.1139289
## row211 0.2235659 -1.802e-01 -1.890e-01 -0.0966456 0.1501080 -0.0865409
## row212 0.1182898 1.805e-01 4.159e-01 0.2807754 0.0176540 0.2523696
## row213 0.0101324 1.921e-01 1.816e-01 0.3064016 0.0493375 -0.0491855
## row214 0.0483743 2.076e-01 3.135e-01 0.1671255 0.2693863 0.1373813
## row215 -0.0893044 2.880e-01 4.042e-02 0.0656518 0.2973356 0.0933976
## row216 -0.0773508 7.841e-02 3.007e-01 0.2078541 0.2097290 0.1118215
## row217 -0.0990042 -1.560e-01 2.287e-01 0.3057795 0.2614404 0.0889148
## row218 0.0046054 -3.374e-02 2.993e-01 0.1329058 0.1908263 0.3793889
## row219 0.0046054 -3.374e-02 2.298e-01 0.2846758 -0.0733827 -0.1214833
## row220 0.0350841 -1.533e-01 -3.513e-01 0.0821753 0.1058997 0.1741406
## row221 0.1033782 -2.061e-01 -1.391e-01 -0.2826699 0.0593396 -0.1225193
## row222 0.1317893 -1.562e-01 5.690e-02 -0.3791809 0.2964554 -0.3135358
## row223 0.1317893 -1.562e-01 6.623e-02 -0.3555447 0.1984113 -0.2862006
## row224 0.0893556 -2.924e-01 -3.193e-02 -0.3027663 -0.0184460 0.1153361
## row225 0.1316877 -2.482e-01 -7.646e-02 -0.3220520 0.2276743 -0.2416541
## row226 0.2784631 -9.754e-02 -9.923e-02 -0.0484564 0.0222574 0.0133875
## row227 0.0961011 -2.318e-01 -7.878e-02 -0.3409779 -0.0980300 0.0471017
## row228 0.1692163 -3.087e-01 -1.983e-02 -0.3161562 -0.0966533 0.0342521
## row229 0.3016550 1.518e-01 2.202e-01 -0.3211589 0.1110717 -0.3615223
## row230 0.3016550 1.518e-01 2.321e-01 -0.0107140 -0.0564160 0.2589023
## row231 0.1160846 8.510e-02 -4.133e-02 -0.2351202 0.0172983 0.2930790
## row232 -0.0292902 1.451e-01 3.602e-01 0.2961631 -0.0170242 0.0406659
## row233 0.0646583 1.015e-01 -1.606e-01 0.2306901 0.0034929 -0.1179410
## row234 0.0423517 1.403e-01 5.257e-02 0.2378998 -0.2134254 -0.2454670
## row235 -0.0517226 1.335e-01 8.180e-02 0.2404392 -0.3734401 -0.4509179
## row236 -0.1251055 -4.077e-02 6.946e-02 0.1837105 -0.2947638 -0.3728647
## row237 -0.0918536 -1.011e-01 1.987e-01 0.2363586 -0.3784944 -0.3154842
## row238 -0.2326216 -2.067e-01 1.364e-01 0.1992052 -0.2554170 -0.2954722
## row239 -0.5417262 -4.208e-02 2.322e-02 -0.2005968 -0.2350914 -0.2122650
## row240 0.0618410 -2.824e-01 -5.195e-02 -0.3472341 0.3649009 -0.3805603
## row241 0.2149272 -2.742e-01 3.775e-04 -0.1869604 -0.3764637 0.2343314
## row242 0.1149337 -2.724e-02 1.105e-01 -0.2542070 -0.0806626 0.3353486
## row243 0.1552593 1.755e-01 6.661e-02 -0.1135934 0.3354718 -0.2697680
## row244 -0.0121202 1.746e-01 1.562e-01 0.0789870 0.0609880 -0.0722148
## row245 0.1687487 1.511e-03 -7.030e-02 0.2508112 0.0067282 -0.0834440
## row246 0.0254753 1.087e-01 9.955e-02 -0.2917922 0.2782775 -0.1150581
## row247 0.0462778 1.269e-01 8.229e-02 0.2172121 -0.0032390 0.0268655
## row248 0.2721769 2.409e-01 4.016e-01 0.3711050 0.0346109 0.0195254
## row249 0.1397284 1.822e-01 3.458e-01 0.1772564 -0.0281205 0.1915107
## row250 0.0442032 9.306e-02 3.710e-01 0.0371362 0.2279452 0.2566310
## row251 0.0442032 9.306e-02 1.270e-01 0.2878169 -0.2537308 -0.2364299
## row252 -0.1885269 -1.909e-01 -6.416e-03 0.1476099 -0.3028266 -0.4040685
## row253 -0.5629034 -1.043e-02 4.827e-03 -0.2837189 -0.1040994 -0.0173185
## row254 0.2433578 3.851e-02 -3.394e-01 0.1910489 -0.1022432 0.1690614
## row255 0.0927618 -2.464e-01 -9.614e-02 -0.2928243 -0.1833784 0.1457162
## row256 0.0026881 -3.102e-01 1.268e-02 -0.5048929 0.0721435 -0.0284919
## row257 0.0546040 -3.065e-01 5.586e-02 -0.3765949 0.3638965 -0.2787072
## row258 0.2149272 -2.742e-01 9.577e-02 -0.3108348 0.4000790 -0.5856795
## row259 0.1571774 -3.217e-01 -5.545e-02 -0.2399073 0.0232505 0.2792371
## row260 0.1516497 -2.236e-01 2.325e-01 0.1885837 0.4461095 0.0689050
## row261 0.0181559 1.738e-01 2.661e-02 -0.2357878 0.1370599 0.1083196
## row262 0.0532692 1.742e-01 9.619e-02 -0.2421764 0.1657991 0.1523235
## row263 0.4045109 1.896e-01 3.366e-01 0.4968690 0.0414879 0.1278403
## row264 0.2562481 2.104e-01 2.897e-01 -0.0588295 0.0441730 0.2906172
## row265 0.2137431 9.940e-02 4.230e-01 0.3108215 0.2043793 0.2222606
## row266 0.2137431 9.940e-02 4.064e-01 0.1657424 0.2118734 0.3995667
## row267 -0.0964343 1.015e-01 -1.269e-02 0.0755240 -0.2131303 -0.1542831
## row268 -0.2670943 -3.154e-01 3.864e-02 0.0635652 -0.2059852 -0.1778452
## row269 0.0166782 -3.218e-01 -2.363e-01 -0.0793891 0.3372462 -0.0823526
## row270 0.2433578 3.851e-02 -2.092e-01 0.0371457 -0.2306531 0.2021167
## row271 0.0280291 -3.996e-01 -2.213e-02 -0.4210582 0.1915996 -0.2974301
## row272 0.1230311 -2.688e-01 -6.579e-02 -0.1197772 0.3920739 -0.1473295
## row273 0.1230311 -2.688e-01 -2.056e-01 -0.0145286 0.3231762 0.0085640
## row274 0.1718645 1.888e-01 3.634e-01 0.1894490 0.1571291 0.3858705
## row275 0.3655623 1.962e-02 9.534e-02 -0.0961660 0.0599228 -0.0050746
## row276 0.2952661 -7.717e-02 6.754e-02 -0.1891631 0.2127665 -0.0706267
## row277 -0.0006716 1.355e-03 2.148e-01 0.2656532 0.1499227 0.2708906
## row278 -0.0178170 9.188e-02 2.214e-01 -0.1629222 0.1740621 0.2665968
## row279 -0.1242669 5.909e-02 3.668e-02 0.1260044 -0.3412572 -0.3397830
## row280 -0.1814727 -1.242e-01 2.405e-01 0.2421297 -0.0348974 -0.0646901
## row281 -0.5435987 -2.881e-02 9.500e-04 -0.2483441 -0.0908877 -0.0545655
## row282 0.0994641 -3.559e-02 -2.609e-01 0.1413117 -0.0276394 0.0746032
## row283 -0.0186165 -3.164e-01 -1.939e-01 -0.1399987 0.3227035 0.0407426
## row284 0.1597582 -2.973e-01 -3.232e-02 -0.3014480 0.2968907 -0.1339014
## row285 0.1597582 -2.973e-01 3.102e-02 -0.1994188 0.4728240 -0.4189173
## row286 0.0715496 -3.479e-01 7.118e-02 0.0428418 0.4161379 -0.1568185
## row287 0.2092914 1.135e-01 -1.359e-01 0.0614933 -0.3183160 0.1436790
## row288 0.2019198 -2.866e-02 -1.421e-01 0.1005785 -0.0212307 0.1779792
## row289 0.2535424 -1.401e-01 4.012e-02 -0.2004878 -0.1862329 0.2333113
## row290 0.2847452 -1.317e-01 -7.040e-03 -0.2487479 0.1289742 -0.1970800
## row291 0.0550738 1.027e-01 3.144e-01 0.1149012 0.1106649 0.3176376
## row292 0.0197155 1.552e-01 2.603e-01 0.2451711 -0.4773575 -0.3618973
## row293 -0.0932453 -2.770e-02 3.050e-02 0.2059198 -0.2308911 -0.2726689
## row294 -0.0052129 -2.855e-01 -5.235e-02 -0.0831323 0.3953253 -0.2399519
## row295 0.0420275 -2.954e-01 -2.329e-01 -0.0494894 0.2812827 0.1088391
## row296 0.1090460 -3.317e-01 3.358e-02 -0.2116214 0.4508355 -0.4478111
## row297 0.0555939 -3.538e-01 -1.064e-01 -0.3061212 -0.1356819 0.2961795
## row298 0.1213878 -1.687e-01 -4.731e-02 -0.0060133 0.1225089 0.0474685
## row299 0.1627547 -2.034e-01 -2.041e-02 -0.2102351 -0.1873295 0.1878689
## row300 0.0728104 -3.450e-02 2.904e-01 -0.0254133 0.3097264 0.0306645
## row301 -0.3050552 -2.690e-01 -2.079e-01 0.1661707 0.0561373 -0.1483181
## row302 -0.2350637 -2.636e-01 -4.730e-02 0.1858056 -0.1573401 -0.3069170
## row303 -0.2394658 -1.634e-01 6.571e-02 0.0855608 -0.3772195 -0.4079961
## row304 -0.1487677 -5.768e-02 1.186e-01 0.1837403 -0.3623480 -0.4054053
## row305 0.2092914 1.135e-01 -2.634e-01 0.0983013 -0.1391350 0.1500190
## row306 -0.0954783 -1.185e-01 -4.075e-01 -0.0986699 -0.0740658 0.1359118
## row307 0.0763165 -6.665e-02 -5.830e-02 -0.1591823 0.0740195 0.1929199
## row308 0.0041495 -3.633e-01 -1.299e-01 -0.2025632 -0.0310174 0.1598026
## row309 0.0836929 -2.994e-01 -5.384e-02 -0.2049044 -0.0328953 0.0780836
## row310 0.1371155 -2.958e-01 -7.358e-02 -0.3101236 -0.0482173 0.1089654
## row311 0.1868383 -2.232e-01 -1.608e-01 0.1024394 0.0421049 0.0792457
## row312 0.1663168 1.467e-02 1.561e-01 -0.1646128 0.1172527 0.1310563
## row313 -0.0696305 -1.503e-01 1.774e-01 0.2625826 -0.0940482 -0.1190991
## row314 -0.2907607 -2.720e-01 9.787e-02 -0.0036324 0.4498647 0.0873229
## row315 -0.2907607 -2.720e-01 2.297e-02 0.0214539 -0.2146492 -0.2048816
## row316 -0.4811928 -1.402e-01 2.147e-02 -0.1903028 -0.1094555 -0.0428210
## row317 -0.0257537 -3.372e-01 -1.377e-01 -0.0431031 0.1916476 0.1300537
## row318 0.1497847 -2.300e-01 -1.886e-02 -0.3354939 -0.1984641 0.1987510
## row319 0.1615986 2.966e-02 2.674e-02 -0.1794617 0.3296267 -0.2574161
## row320 -0.1298226 -2.960e-01 -1.954e-02 0.2268814 -0.1130847 -0.1667477
## row321 -0.2061893 3.551e-02 6.351e-02 0.0255926 -0.2244267 -0.1969638
## row322 -0.0493579 -3.051e-01 -1.952e-01 -0.2129033 -0.1456806 0.2486883
## row323 0.0397069 -2.592e-01 -4.782e-02 -0.1255414 0.0637406 0.2784200
## row324 0.0690591 -2.648e-01 -1.277e-01 -0.0954488 0.0184955 0.0303453
## row325 0.1247549 8.756e-02 -2.381e-02 -0.2467764 -0.4918892 0.2709115
## row326 0.1957106 -1.230e-02 -1.116e-01 -0.1217310 -0.3051237 0.2069965
## row327 -0.3778478 -3.936e-01 -1.026e-01 -0.3147719 0.3224880 0.1474171
## row328 -0.4725637 -3.193e-01 -7.799e-02 -0.2607538 0.1563539 0.2873606
## row329 -0.4041890 1.756e-01 7.875e-02 -0.2063914 -0.2566489 -0.1683570
## row330 -0.1837819 -6.318e-02 1.420e-01 0.0775184 -0.3372923 -0.2757186
## row331 -0.3601083 1.217e-01 1.031e-01 -0.1642943 -0.1917616 -0.0854565
## row332 0.0122027 3.063e-01 -2.912e-01 0.0004458 -0.0480493 0.1573228
## row333 -0.0361510 4.022e-01 -2.198e-01 -0.1740281 -0.1625558 0.1973167
## row334 -0.0406412 3.527e-01 -3.089e-01 0.0162442 0.0383273 0.0523795
## row335 0.1195621 3.228e-01 -5.883e-02 -0.2866334 -0.2186847 0.2482353
## row336 -0.0817127 5.428e-01 -2.682e-01 -0.0927415 -0.1107771 0.1048170
## row337 -0.0424174 2.916e-01 -1.763e-02 -0.3677030 -0.1401633 0.1365972
## row338 -0.0721192 4.062e-01 -2.742e-01 0.0155985 -0.0502391 0.0885922
## row339 0.0036732 3.179e-01 -3.257e-01 0.0098588 0.0130005 0.1010411
## row340 -0.0670300 3.706e-01 -2.619e-01 -0.0818981 -0.1344905 0.0697959
## row341 -0.0357419 2.653e-01 -1.913e-01 0.0122900 0.0222664 -0.0507951
## row342 -0.2335173 3.557e-01 -2.295e-01 -0.0330094 0.1417705 0.0027476
## row343 -0.0272044 2.830e-01 -3.312e-01 0.0575309 0.0350474 0.0959181
## row344 -0.0136254 2.630e-01 -3.626e-01 0.1577793 0.0659636 -0.0062816
## row345 0.1216303 1.119e-01 -2.379e-01 0.1769067 0.0682505 0.1362739
## row346 0.0565421 7.673e-02 -3.721e-01 0.2168524 0.0663800 0.0242383
## row347 -0.1457687 3.748e-01 -2.130e-01 -0.1442756 0.0152198 0.1785453
## row348 -0.0136254 2.630e-01 -3.037e-01 0.1040434 0.0844215 0.0732537
## row349 0.0876036 4.060e-02 -2.668e-01 0.0696335 -0.1037720 0.1661616
## row350 -0.1879606 4.506e-01 -1.478e-01 -0.0748502 0.0443362 -0.0109303
## row351 -0.3168261 4.981e-01 -3.040e-01 -0.1279295 0.0967059 0.0188710
## row352 -0.1098702 3.346e-01 -3.294e-01 0.1096577 0.1067464 -0.0186936
## row353 0.1415020 -4.063e-02 -3.235e-01 0.2887699 0.0590078 -0.0492649
## row354 -0.2413650 3.501e-01 -3.178e-01 -0.0105986 0.0853915 -0.0841154
## row355 -0.2160471 3.885e-01 -1.925e-01 -0.0010086 0.0744513 -0.1554344
## row356 -0.1875179 4.015e-01 -2.217e-01 0.0081904 0.0710458 -0.1372061
## row357 0.0036831 2.793e-01 -3.041e-01 0.2005010 0.1067114 -0.0105744
## row358 0.0252013 2.578e-01 -1.148e-01 0.1119280 -0.0554695 -0.0476007
## row359 -0.0561467 2.477e-01 -1.013e-01 0.0771389 0.0491685 -0.0848041
## row360 -0.0296910 1.310e-01 -2.506e-01 0.1309965 0.0870747 -0.0424291
## row361 0.0462332 9.642e-02 -3.395e-01 0.1276867 0.0870617 0.1128584
## row362 0.2089906 -9.082e-02 -2.738e-01 0.1264981 0.0100287 0.0837725
## row363 0.0767910 -3.066e-01 -8.672e-02 -0.2722089 -0.1556110 -0.0117563
## row364 0.0767910 -3.066e-01 -1.203e-01 -0.3281453 -0.1533518 0.0597444
## row365 -0.4292477 4.983e-01 -1.983e-01 -0.1466902 0.0919047 -0.1482148
## row366 -0.3067247 3.654e-01 -2.712e-01 -0.1427093 0.0313154 -0.0660755
## row367 -0.0125557 2.799e-01 -2.739e-01 0.1628264 0.0158522 -0.0548594
## row368 -0.0125557 2.799e-01 -2.394e-01 0.0593560 0.0195063 -0.0148273
## row369 -0.0296910 1.310e-01 -3.519e-01 0.1731819 0.1189899 0.0471182
## row370 -0.0887360 8.540e-02 -3.456e-01 0.1212960 0.0835381 -0.0546197
## row371 0.1251539 1.002e-01 -3.458e-01 0.1646618 0.0512198 0.1289918
## row372 0.1861697 -7.360e-02 -3.246e-01 0.3019158 0.0665971 0.0402653
## row373 0.1668867 -7.668e-02 -2.979e-01 0.3054687 -0.0165957 0.0132088
## row374 0.2929960 -4.260e-02 -2.904e-01 0.2745163 0.0590707 0.0873284
## row375 -0.2974021 3.496e-01 -3.249e-01 -0.0939291 0.0298112 -0.0249595
## row376 -0.1256340 3.426e-01 -2.477e-01 0.0537940 0.0719116 -0.1498246
## row377 -0.2750421 3.720e-01 -1.691e-01 -0.2045047 0.0722768 0.0022554
## row378 -0.2622270 3.651e-01 -2.245e-01 0.0075852 0.0869008 -0.1148197
## row379 0.1340190 8.635e-02 -3.244e-01 0.2862012 0.0372968 -0.0817570
## row380 0.1340190 8.635e-02 -2.559e-01 0.2547517 0.0676635 0.0538997
## row381 0.1528812 -1.541e-01 -3.478e-01 0.1307106 0.2069684 0.0657545
## row382 -0.2974021 3.496e-01 -3.315e-01 -0.1501093 0.0410792 0.0046638
## row383 -0.2750421 3.720e-01 -2.754e-01 -0.0838538 0.0906406 0.0583582
## row384 -0.2622270 3.651e-01 -3.568e-01 -0.0178815 0.1195547 -0.0466450
## row385 0.1340190 8.635e-02 -2.077e-01 0.2537831 0.1138727 -0.0754871
## row386 -0.0837327 1.565e-01 -2.873e-01 0.1474601 0.1125082 -0.1172322
## row387 -0.1690155 9.522e-02 -3.322e-01 0.0549107 0.0981670 -0.1029660
## row388 0.1954943 4.824e-02 -3.229e-01 0.3613070 0.0543069 -0.0953373
## row389 -0.1538960 1.209e-01 -2.950e-01 0.0835657 0.1127128 -0.1087355
## row390 -0.0500354 1.499e-01 -2.834e-01 0.0029947 -0.0609547 0.0634503
## row391 -0.1668734 1.958e-01 -2.801e-01 -0.0021596 0.0761178 -0.1371291
## row392 0.0026480 4.587e-02 -1.916e-01 0.1337777 -0.0224931 -0.1249927
## row393 0.0662274 -3.481e-03 -2.099e-01 0.1843101 -0.0007311 -0.1348903
## row394 -0.1189219 8.364e-03 -3.710e-01 0.1061733 0.1262350 -0.0865008
## row395 0.0185785 -4.854e-02 -3.636e-01 0.2011268 0.0783115 0.0229193
## row396 0.0350841 -1.533e-01 -4.341e-01 0.2209991 0.1803378 0.0527031
## row397 0.0767910 -3.066e-01 -1.014e-01 -0.2410308 0.0487115 0.1717279
## row398 0.0767910 -3.066e-01 -1.287e-01 -0.2663653 -0.2805819 0.2289558
## row399 0.0738839 2.843e-02 -2.902e-01 0.2278822 0.0439496 -0.1170225
## row400 0.0352179 -8.667e-02 -3.577e-01 0.2457587 0.0841843 -0.1107608
## row401 0.0577775 -7.882e-02 -2.499e-01 0.1774877 0.0113380 0.0406807
## row402 0.1055856 -6.472e-02 -2.920e-01 0.2759179 0.0778414 -0.1444217
## row403 0.0360584 -1.034e-02 -3.644e-01 0.2202518 0.1093809 -0.0877470
## row404 0.1055856 -6.472e-02 -2.907e-01 0.2313020 0.0812285 -0.0566156
## row405 -0.0007242 -2.361e-01 -3.750e-01 0.2737005 0.1355194 -0.0988141
## row406 -0.0054329 -1.016e-02 -4.005e-01 0.1403306 0.0873335 0.0368998
## row407 0.1387167 -1.917e-01 -2.058e-03 -0.3594957 0.2427913 -0.4385907
## row408 0.1195621 3.228e-01 1.084e-01 -0.4816169 -0.1007727 -0.0171326
## row409 0.2044175 3.741e-02 1.551e-01 -0.2084262 0.3474323 -0.3839959
## row410 0.2044175 3.741e-02 -2.825e-01 0.2645575 0.1083676 -0.0211197
## row411 0.0994641 -3.559e-02 7.313e-02 -0.1083193 -0.0573447 0.1477163
## row412 0.0994641 -3.559e-02 1.264e-01 0.2743248 0.2093527 0.1318480
## row413 -0.0054329 -1.016e-02 -3.058e-01 0.1354262 0.0461216 -0.1066651
## row414 0.0555528 -2.033e-01 -2.278e-01 -0.1986050 0.2428654 -0.3313752
## row415 0.0555528 -2.033e-01 -1.812e-01 -0.0578948 0.2827060 -0.2230556
## row416 -0.4766281 -5.737e-02 7.615e-02 -0.2690893 -0.0401608 0.1061439
## row417 -0.4041890 1.756e-01 9.710e-02 -0.2074297 -0.1943617 -0.1215599
##
##
## Biplot scores for constraining variables
##
## RDA1 RDA2 PC1 PC2 PC3 PC4
## jes 0.8503 0.5263 0 0 0 0
## guaranis -0.5788 0.8155 0 0 0 0

plot(ord_sp)


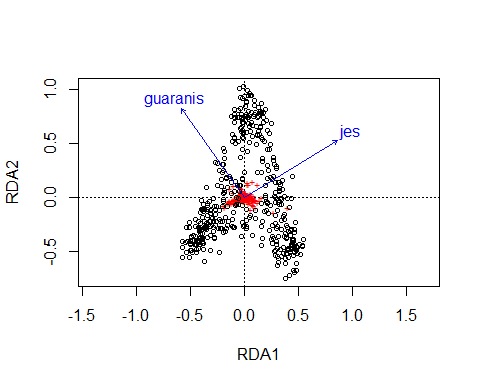


vif.cca(ord_sp)

## jes guaranis
## 1.003977 1.003977

sp_scores<-scores(ord_sp, choices=c(1,2), display="species")
write.csv(sp_scores, file = "sp_rda_scores.csv", sep=",", dec=".")

## Warning in write.csv(sp_scores, file = "sp_rda_scores.csv", sep = ",", dec
## = "."): attempt to set 'sep' ignored

## Warning in write.csv(sp_scores, file = "sp_rda_scores.csv", sep = ",", dec
## = "."): attempt to set 'dec' ignored

#abbreviates species names

spnames<-make.cepnames(colnames(sp))
ptcols <- brewer.pal(3, "Dark2")

fisio <- co_occ$fitofisionomia
rda.tab <- summary(ord_sp)$cont$importance

spe.cols <- ifelse(abs(scores(ord_sp, display="species", choice=1)) > 0.1 |
 abs(scores(ord_sp, display="species", choice=2)) > 0.1, "black", "grey")


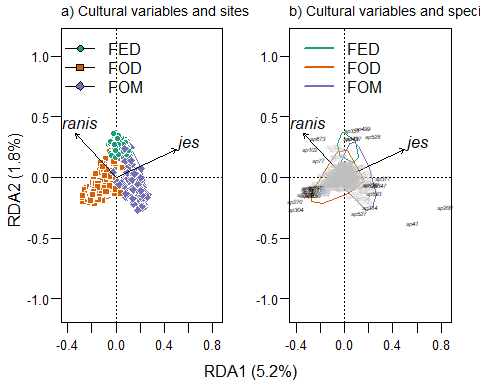
 ## S3 - Environmental Niche

library(raster)

## Loading required package: sp

library(sp)
library(rgeos)

## rgeos version: 0.3-28, (SVN revision 572)
## GEOS runtime version: 3.6.1-CAPI-1.10.1 r0
## Linking to sp version: 1.3-1
## Polygon checking: TRUE

library(rgdal)

## rgdal: version: 1.3-4, (SVN revision 766)
## Geospatial Data Abstraction Library extensions to R successfully loaded
## Loaded GDAL runtime: GDAL 2.2.3, released 2017/11/20
## Path to GDAL shared files: C:/Users/aline/Documents/R/win-library/3.5/rgdal/gdal
## GDAL binary built with GEOS: TRUE
## Loaded PROJ.4 runtime: Rel. 4.9.3, 15 August 2016, [PJ_VERSION: 493]
## Path to PROJ.4 shared files: C:/Users/aline/Documents/R/win-library/3.5/rgdal/proj
## Linking to sp version: 1.3-1

library(fields)

## Loading required package: spam

## Loading required package: dotCall64

## Loading required package: grid

## Spam version 2.2-0 (2018-06-19) is loaded.
## Type 'help( Spam)' or 'demo( spam)' for a short introduction
## and overview of this package.
## Help for individual functions is also obtained by adding the
## suffix '.spam' to the function name, e.g. 'help( chol.spam)'.

##
## Attaching package: 'spam'

## The following objects are masked from 'package:base':
##
## backsolve, forwardsolve

## Loading required package: maps

##
## Attaching package: 'maps'

## The following object is masked from 'package:plyr':
##
## ozone

## See www.image.ucar.edu/~nychka/Fields for
## a vignette and other supplements.

library(shapefiles)

## Loading required package: foreign

##
## Attaching package: 'shapefiles'

## The following objects are masked from 'package:foreign':
##
## read.dbf, write.dbf

library(maptools)

## Checking rgeos availability: TRUE

library(maps)


occup<-shapefile("S3_BOTH_OCC.shp")#high probability of Souther-Je or Garani occurrence area, create in Qgis using the ENM results rasters by "Poligonize Raster" function.
occup<-spTransform(occup, CRS('+init=EPSG:31982'))
occup2<-spTransform(occup, CRS("+proj=longlat +datum=WGS84"))
ext_occup<-extent(occup)
ext_occup2<-extent(occup2)

#Import and standardize the most importat vabiables in ecological niche models: elevation, sea distance, main rivers distance, third order (intermediate) rivers distance

elevation<-raster("h1k_dem.asc")#download file in Ambdata
projection(elevation)<-"+proj=longlat +datum=WGS84"
elevation<-crop(elevation,ext_occup2)
limit_occup2.r<-rasterize(occup2, elevation)
elevation<-elevation * limit_occup2.r
range(elevation)#min 0 max 1681

## Warning in range(new("RasterLayer", file = new(".RasterFile", name = "", :
## Nothing to summarize if you provide a single RasterLayer; see cellStats

## class : RasterLayer
## dimensions : 1550, 1210, 1875500 (nrow, ncol, ncell)
## resolution : 0.009, 0.009 (x, y)
## extent : -58.59033, -47.70033, -34.29333, -20.34333 (xmin, xmax, ymin, ymax)
## coord. ref. : +proj=longlat +datum=WGS84 +ellps=WGS84 +towgs84=0,0,0
## data source : in memory
## names : layer
## values : 0, 1681 (min, max)

sea<-raster("S3_SEA.tif")
crs(sea) <- CRS('+init=EPSG:31982')
sea<-crop(sea,ext_occup)
limit_occup.r<-rasterize(occup,sea)
sea<-sea *limit_occup.r
sea<-sea*10#correct metric
range(sea)#min 0 max 470

## Warning in range(new("RasterLayer", file = new(".RasterFile", name = "", :
## Nothing to summarize if you provide a single RasterLayer; see cellStats

## class : RasterLayer
## dimensions : 1567, 1060, 1661020 (nrow, ncol, ncell)
## resolution : 1000, 1000 (x, y)
## extent : -226589.5, 833410.5, 6183484, 7750484 (xmin, xmax, ymin, ymax)
## coord. ref. : +init=EPSG:31982 +proj=utm +zone=22 +south +ellps=GRS80 +towgs84=0,0,0,0,0,0,0 +units=m +no_defs
## data source : in memory
## names : layer
## values : 0, 470 (min, max)

main<-raster("S3_MAIN.tif")
crs(main) <- CRS('+init=EPSG:31982')
main<-crop(main,ext_occup)
main<-resample(main,sea, method="bilinear")
main<-main *limit_occup.r
main<-main*10
range(main)#0-29

## Warning in range(new("RasterLayer", file = new(".RasterFile", name = "", :
## Nothing to summarize if you provide a single RasterLayer; see cellStats

## class : RasterLayer
## dimensions : 1567, 1060, 1661020 (nrow, ncol, ncell)
## resolution : 1000, 1000 (x, y)
## extent : -226589.5, 833410.5, 6183484, 7750484 (xmin, xmax, ymin, ymax)
## coord. ref. : +init=EPSG:31982 +proj=utm +zone=22 +south +ellps=GRS80 +towgs84=0,0,0,0,0,0,0 +units=m +no_defs
## data source : in memory
## names : layer
## values : 0, 290 (min, max)

third<-raster("S3_THIRD.tif")
crs(third) <- CRS('+init=EPSG:31982')
third<-crop(third,ext_occup)
third<-resample(third,sea, method="bilinear")
third<-third * limit_occup.r
third<-third*10
range(third)# 0 - 19

## Warning in range(new("RasterLayer", file = new(".RasterFile", name = "", :
## Nothing to summarize if you provide a single RasterLayer; see cellStats

## class : RasterLayer
## dimensions : 1567, 1060, 1661020 (nrow, ncol, ncell)
## resolution : 1000, 1000 (x, y)
## extent : -226589.5, 833410.5, 6183484, 7750484 (xmin, xmax, ymin, ymax)
## coord. ref. : +init=EPSG:31982 +proj=utm +zone=22 +south +ellps=GRS80 +towgs84=0,0,0,0,0,0,0 +units=m +no_defs
## data source : in memory
## names : layer
## values : 0, 190 (min, max)

#Create 1000 random points into each cultural group area of highest probability of occupation;

#to elevation
je_occ2<-shapefile("S3_JE.shp")
je_occ2<-spTransform(je_occ2, CRS('+init=EPSG:31982'))
ptsJe2 <- spsample(je_occ2, 1000, type = 'random')

gua_occ2<-shapefile("S3_GUA.shp")
gua_occ2<-spTransform(gua_occ2, CRS('+init=EPSG:31982'))
ptsGua2 <- spsample(gua_occ2, 1000, type = 'random')

#to other variables
je_occ<-shapefile("S3_JE.shp")
projection(je_occ)<-"+proj=longlat +datum=WGS84"
ptsJe <- spsample(je_occ, 1000, type = 'random')

gua_occ<-shapefile("S3_GUA.shp")
projection(gua_occ)<-"+proj=longlat +datum=WGS84"
ptsGua <- spsample(gua_occ, 1000, type = 'random')

#Extract topographic and hydrographic values to each cultural group points
ele_je<-as.data.frame(extract(elevation,ptsJe2))

## Warning in .local(x, y, ...): Transforming SpatialPoints to the CRS of the
## Raster

ele_gua<-as.data.frame(extract(elevation,ptsGua2))

## Warning in .local(x, y, ...): Transforming SpatialPoints to the CRS of the
## Raster

# remove NA's
ele_je <- na.omit(ele_je)
ele_gua<-na.omit(ele_gua)
elevationJe<-write.csv(ele_je, file="elevation_je.csv", row.names=T)
elevationGua<-write.csv(ele_gua, file="elevation_guaranis.csv", row.names=T)

main_je<-as.data.frame(extract(main,ptsJe))

## Warning in .local(x, y, ...): Transforming SpatialPoints to the CRS of the
## Raster

main_gua<-as.data.frame(extract(main,ptsGua))

## Warning in .local(x, y, ...): Transforming SpatialPoints to the CRS of the
## Raster

main_je <- na.omit(main_je)
main_gua<-na.omit(main_gua)
main_river_je<-write.csv(main_je, file="main_riversJe.csv", row.names=T)
main_river_gua<-write.csv(main_gua, file="main_rivers_guaranis.csv", row.names=T)

third_je<-as.data.frame(extract(third,ptsJe))

## Warning in .local(x, y, ...): Transforming SpatialPoints to the CRS of the
## Raster

third_gua<-as.data.frame(extract(third,ptsGua))

## Warning in .local(x, y, ...): Transforming SpatialPoints to the CRS of the
## Raster

third_je <- na.omit(third_je)
third_gua<-na.omit(third_gua)
third_rivers_je<-write.csv(third_je, file="third_rivers_Je.csv", row.names=T)
third_rivers_gua<-write.csv(third_gua, file="third_rivers_Guarani.csv", row.names=T)

sea_je<-as.data.frame(extract(sea,ptsJe))

## Warning in .local(x, y, ...): Transforming SpatialPoints to the CRS of the
## Raster

sea_gua<-as.data.frame(extract(sea,ptsGua))

## Warning in .local(x, y, ...): Transforming SpatialPoints to the CRS of the
## Raster

sea_je <- na.omit(sea_je)
sea_gua<-na.omit(sea_gua)
sea_dist_je<-write.csv(sea_je, file="seaJe.csv", row.names=T)
sea_dist_guagua<-write.csv(sea_gua, file="seaGuarani.csv", row.names=T)

#Tables with the values of each variable for the two cultural groups were set up in an electronic spreadsheet, with one table for each variable.


#Create Graphs
library(ggplot2)
values<-read.csv("S3_values.csv", header=T, sep=",", dec=".")


#create elevation graph
H<-values[,-c(3:5)]
names(H)<-c("cult","h")
head(H)

## cult h
## 1 guarani 512
## 2 guarani 196
## 3 guarani 155
## 4 guarani 197
## 5 guarani 288
## 6 guarani 757

breaks = c(0,250,500,750,1000,1250,1500, 1750)
 labels = as.character(breaks)
 plot.h = ggplot(H, aes(x=h, y=..density..)) +
 geom_density(aes(fill=cult,color= 'h'), alpha=.5)+
 geom_vline(aes(xintercept=mean(h)),color="darkorange",linetype="solid",size=1)+
geom_vline(aes(xintercept=quantile(h,0.975)),color="darkorange",linetype="dashed",size=1)+
 geom_vline(aes(xintercept=quantile(h,0.025)),color="darkorange",linetype="dashed",size=1)+
 geom_density(aes(h, fill=cult,color='h'),alpha=.5) +
 geom_vline(aes(xintercept=mean(h)),color="black",linetype="solid",size=1)+
 geom_vline(aes(xintercept=quantile(h,0.975)),color="black",linetype="dashed",size=1) +
 geom_vline(aes(xintercept=quantile(h,0.025)),color="black",linetype="dashed",size=1)+
 geom_vline(xintercept = c(0, 1750), colour="gray70", linetype="dashed") +
 scale_x_continuous(limits = c(0, 1750), breaks = breaks, labels = labels)+
 scale_color_manual(values = c('h' = 'darkorange', 'h '= 'black'))
 plot.h = plot.h + theme(panel.grid.major = element_blank(), panel.grid.minor = element_blank(),
 legend.text = element_text(face= "italic", size=10),
 panel.background = element_blank(), axis.line = element_line(colour = "black")) +
 labs(x="Elevation (m)", y = "Density of Occurrence Probability",size=10) +
 labs(col = "") +
 theme(axis.title.x = element_text(size = rel(1))) +
 theme(axis.title.y = element_text(size = rel(1))) +
 theme(axis.text.x = element_text(size = rel(1))) +
 theme(axis.text.y = element_text(size = rel(1)))
 ggsave("elevation.pdf",plot=plot.h,width=20,height=10)
print(plot.h)


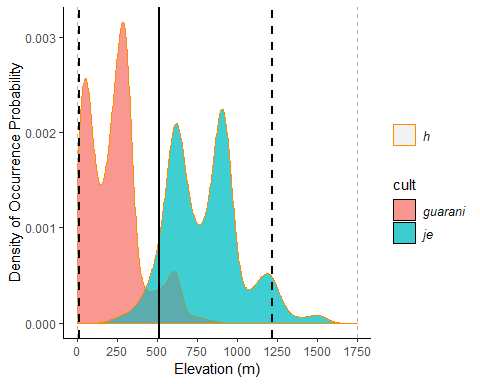


#create coastline distance graph
sea<-values[,-c(2:4)]
names(sea)<-c("cult","dist")
head(sea)

## cult dist
## 1 guarani 0
## 2 guarani 0
## 3 guarani 90
## 4 guarani 410
## 5 guarani 180
## 6 guarani 380

head(sea)

## cult dist
## 1 guarani 0
## 2 guarani 0
## 3 guarani 90
## 4 guarani 410
## 5 guarani 180
## 6 guarani 380

breaks = c(0,150,300,450, 600)
 labels = as.character(breaks)
 plot.sea = ggplot(sea, aes(x=dist, y=..density..)) +
 geom_density(aes(fill=cult,color= 'dist'), alpha=.5)+
 geom_vline(aes(xintercept=mean(dist)),color="darkorange",linetype="solid",size=1)+
 geom_vline(aes(xintercept=quantile(dist,0.975)),color="darkorange",linetype="dashed",size=1)+
 geom_vline(aes(xintercept=quantile(dist,0.025)),color="darkorange",linetype="dashed",size=1)+
 geom_density(aes(dist, fill=cult,color='dist'),alpha=.5) +
 geom_vline(aes(xintercept=mean(dist)),color="black",linetype="solid",size=1)+
 geom_vline(aes(xintercept=quantile(dist,0.975)),color="black",linetype="dashed",size=1) +
 geom_vline(aes(xintercept=quantile(dist,0.025)),color="black",linetype="dashed",size=1)+
 geom_vline(xintercept = c(0, 600), colour="gray70", linetype="dashed") +
 scale_x_continuous(limits = c(0, 600), breaks = breaks, labels = labels)+
 scale_color_manual(values = c('dist' = 'darkorange', 'h '= 'black'))
 plot.sea = plot.sea + theme(panel.grid.major = element_blank(), panel.grid.minor = element_blank(),
 legend.text = element_text(face= "italic", size=10),
 panel.background = element_blank(), axis.line = element_line(colour = "black")) +
 labs(x="Coastline Distance (Km)", y = "Density of Occurrence Probability",size=10) +
 labs(col = "") +
 theme(axis.title.x = element_text(size = rel(1))) +
 theme(axis.title.y = element_text(size = rel(1))) +
 theme(axis.text.x = element_text(size = rel(1))) +
 theme(axis.text.y = element_text(size = rel(1)))
 # ggsave("sea.pdf",plot=plot.sea,width=20,height=10)
print(plot.sea)


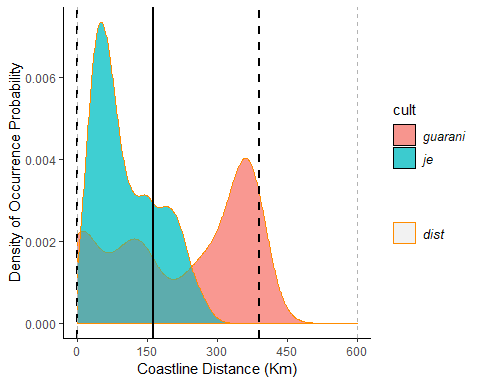


#create main rivers distance graph
main<-values[,-c(2,4,5)]
names(main)<-c("cult","distance")
head(main)

## cult distance
## 1 guarani 0
## 2 guarani 0
## 3 guarani 0
## 4 guarani 10
## 5 guarani 0
## 6 guarani 10

breaks = c(0,100,200,300)
 labels = as.character(breaks)
 plot.main = ggplot(main, aes(x=distance, y=..density..)) +
 geom_density(aes(fill=cult,color= 'distance'), alpha=.5)+
 geom_vline(aes(xintercept=mean(distance)),color="darkorange",linetype="solid",size=1)+
geom_vline(aes(xintercept=quantile(distance,0.975)),color="darkorange",linetype="dashed",size=1)+
 geom_vline(aes(xintercept=quantile(distance,0.025)),color="darkorange",linetype="dashed",size=1)+
 geom_density(aes(distance, fill=cult,color='distance'),alpha=.5) +
 geom_vline(aes(xintercept=mean(distance)),color="black",linetype="solid",size=1)+
 geom_vline(aes(xintercept=quantile(distance,0.975)),color="black",linetype="dashed",size=1) +
 geom_vline(aes(xintercept=quantile(distance,0.025)),color="black",linetype="dashed",size=1)+
 geom_vline(xintercept = c(0, 300), colour="gray70", linetype="dashed") +
 scale_x_continuous(limits = c(0, 300), breaks = breaks, labels = labels)+
 scale_color_manual(values = c('distance' = 'darkorange', 'h '= 'black'))
 plot.main = plot.main + theme(panel.grid.major = element_blank(), panel.grid.minor = element_blank(),
 legend.text = element_text(face= "italic", size=10),
 panel.background = element_blank(), axis.line = element_line(colour = "black")) +
 labs(x="Main Rivers Distance (Km)", y = "Density of Occurrence Probability",size=10) +
 labs(col = "") +
 theme(axis.title.x = element_text(size = rel(1))) +
 theme(axis.title.y = element_text(size = rel(1))) +
 theme(axis.text.x = element_text(size = rel(1))) +
 theme(axis.text.y = element_text(size = rel(1)))
ggsave("mainrivers.pdf",plot=plot.main,width=20,height=10)
 print(plot.main)


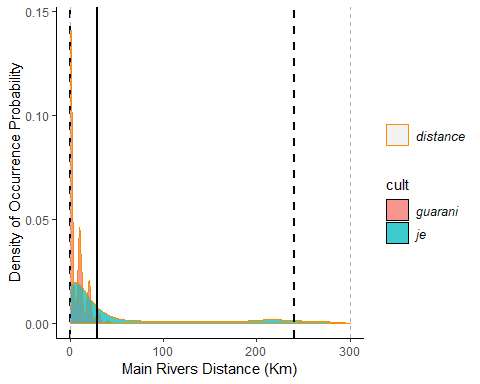


#create third rivers distance graph
third<-values[,-c(2,3,5)]
names(third)<-c("cult","distance")
head(third)

## cult distance
## 1 guarani 0.000000
## 2 guarani 30.000000
## 3 guarani 0.000000
## 4 guarani 10.000000
## 5 guarani 0.000000
## 6 guarani 7.920211

breaks = c(0,50,100,200)
 labels = as.character(breaks)
 plot.third = ggplot(third, aes(x=distance, y=..density..)) +
 geom_density(aes(fill=cult,color= 'distance'), alpha=.5)+
 geom_vline(aes(xintercept=mean(distance)),color="darkorange",linetype="solid",size=1)+
geom_vline(aes(xintercept=quantile(distance,0.975)),color="darkorange",linetype="dashed",size=1)+
 geom_vline(aes(xintercept=quantile(distance,0.025)),color="darkorange",linetype="dashed",size=1)+
 geom_density(aes(distance, fill=cult,color='distance'),alpha=.5) +
 geom_vline(aes(xintercept=mean(distance)),color="black",linetype="solid",size=1)+
 geom_vline(aes(xintercept=quantile(distance,0.975)),color="black",linetype="dashed",size=1) +
 geom_vline(aes(xintercept=quantile(distance,0.025)),color="black",linetype="dashed",size=1)+
 geom_vline(xintercept = c(0, 300), colour="gray70", linetype="dashed") +
 scale_x_continuous(limits = c(0, 300), breaks = breaks, labels = labels)+
 scale_color_manual(values = c('distance' = 'darkorange', 'h '= 'black'))
 plot.third = plot.third + theme(panel.grid.major = element_blank(), panel.grid.minor = element_blank(),
 legend.text = element_text(face= "italic", size=10),
 panel.background = element_blank(), axis.line = element_line(colour = "black")) +
 labs(x="third Rivers Distance (Km)", y = "Density of Occurrence Probability",size=10) +
 labs(col = "") +
 theme(axis.title.x = element_text(size = rel(1))) +
 theme(axis.title.y = element_text(size = rel(1))) +
 theme(axis.text.x = element_text(size = rel(1))) +
 theme(axis.text.y = element_text(size = rel(1)))
ggsave("thirdrivers.pdf",plot=plot.third,width=20,height=10)
 print(plot.third)


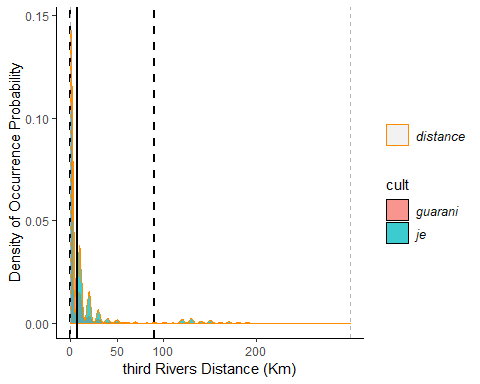
 ## S2 - Niche Overlap

library(ENMeval)

## Loading required package: dismo

library(raster)
library(sp)
library(rgeos)
library(rgdal)
library(fields)
library(shapefiles)
library(maptools)
library(maps)
#import ENM results, named "S1A_result.tif" and "S2A_result.tif"

#import Souther-Je occurrence limit shapefile and use to crop rasters in the same size

je_occ<-shapefile("S1B.shp")
je_occ<-spTransform(je_occ, CRS("+proj=longlat +datum=WGS84"))

#import ENM results, named "S1A_result.tif" and "S2A_result.tif"

S1A<-raster("S1A_result.tif")#Guarani
projection(S1A)<-"+proj=longlat +datum=WGS84"
S1A<-crop(S1A,extent(je_occ))

S1B<-raster("S1B_result.tif")#Southern-Je
projection(S1B)<-"+proj=longlat +datum=WGS84"
S1B<-crop(S1B,extent(je_occ))

#remove NAs
S1A[is.na(S1A)] <- 999
S1B[is.na(S1B)] <- 999

#stack
cult<-stack(S1A,S1B)
names(cult)<-c('Guarani','Je')
projection(cult)<-"+proj=longlat +datum=WGS84"

#calc overlap niche
overlap<-calc.niche.overlap(cult, stat = "I", maxent.args(RMvalues = seq(0.5, 4, 0.5),
fc = c("L", "LQ", "H", "LQH", "LQHP", "LQHPT"),
labels = TRUE))

##
 |
 | | 0%
 |
 |=================================================================| 100%

summary(overlap)

## Guarani Je
## Min. :0.6924 Min. : NA
## 1st Qu.:0.6924 1st Qu.: NA
## Median :0.6924 Median : NA
## Mean :0.6924 Mean :NaN
## 3rd Qu.:0.6924 3rd Qu.: NA
## Max. :0.6924 Max. : NA
## NA's :1 NA's :2

## S1 - Niche Ecological Models (ENM)

**Final Results in raster format are available for download. See “S1A_result.tif” and “S2A_result.tif”

library(raster)
library(sp)
library(rgeos)
library(rgdal)
library(fields)
library(shapefiles)
library(maptools)
library(maps)
library(rJava)

#### S1A - Guarani

# Import The Occurrence Limit#
#Download the file named **"S1A.shp"**, available in supplementary material. This shapefile corresponds to The "Area of Guarani Occurrence", and was preliminarily delimited in the Qgis Software.


gua_occ<-shapefile("S1A.shp")#Guarani Occurrence Limit
gua_occ<-spTransform(gua_occ, CRS("+proj=longlat +datum=WGS84"))#Enter the coordinate system

#Import and standardize topographic and hydrographic variables
#Download Elevation and Slope variables in Ambdata (http://www.dpi.inpe.br/Ambdata/)

#HAND variable is available in Ambdata, but was processed in the QGis Software to increase pixel size, using GRASS command "r.resamp.interp". This 10 km pixel raster is available for download in the supplementary material, with the name **"SA_HAND.tif"**.

#Coastline Distance and Water Courses distance are available in the supplementary material, with the names: **"SA_SEA.tif"**; **"SA_1_2.tif"**; **"SA_3.tif"**; **"SA_4.tif"**; **"SA_5_8.tif"**. Distance rasters was generated using Qgis.

#Height Above the Nearest Drainage (HAND)
hand<-raster("S1_HAND.tif")
projection(hand)<-"+proj=longlat +datum=WGS84"
ext.gua_occ<-extent(gua_occ)
hand<-crop(hand,extent(gua_occ))
limit_gua.r<-rasterize(gua_occ, hand)
hand.gua<-hand * limit_gua.r
ext.limit_gua<-extent(gua_occ)
hand.gua<-crop(hand.gua, ext.limit_gua)
hand.mask<- hand.gua
hand.mask[hand.gua >= minValue(hand.gua)] <- 1
#Elevation
elevation<-raster("h1k_dem.asc")#download file in Ambdata
projection(elevation)<-"+proj=longlat +datum=WGS84"
elevation<-crop(elevation,ext.limit_gua)
elevation<-resample(elevation,hand, method="bilinear")
elevation<-elevation * limit_gua.r
elevation.mask<-elevation
elevation.mask[elevation >= minValue(elevation)] <- 1
#Slope
slope<-raster("h1k_slope.asc")
projection(slope)<-"+proj=longlat +datum=WGS84"
slope<-crop(slope,ext.limit_gua)
slope<-resample(slope,hand.gua, method="bilinear")
slope<-slope * limit_gua.r
slope.mask<-slope
slope.mask[slope >= minValue(slope)] <- 1
#Coastline Distance
sea<-raster("S1_SEA.tif")
projection(sea)<-"+proj=longlat +datum=WGS84"
sea<-crop(sea,ext.limit_gua)
sea<-resample(sea,hand.gua,method="bilinear")
sea<-sea * limit_gua.r
sea.mask<-sea
sea.mask[sea >= minValue(sea)] <- 1

#Water Courses
#1st and 2nd River Classes Distance (Main Rivers)
main<-raster("S1_1ST2ND.tif")
projection(main)<-"+proj=longlat +datum=WGS84"
main<-crop(main,ext.limit_gua)
main<-resample(main,hand.gua, method="bilinear")
main<-main * limit_gua.r
main.mask<-main
main.mask[main >= minValue(main)] <- 1
#3rd River Class Distance
third<-raster("S1_3RD.tif")
projection(third)<-"+proj=longlat +datum=WGS84"
third<-crop(third,ext.limit_gua)
third<-resample(third,hand.gua, method="bilinear")
third<-third * limit_gua.r
third.mask<-third
third.mask[third>= minValue(third)] <- 1
#4th River Class Distance
fourth<-raster("S1_4TH.tif")
projection(fourth)<-"+proj=longlat +datum=WGS84"
fourth<-crop(fourth,ext.limit_gua)
fourth<-resample(fourth,hand.gua, method="bilinear")
fourth<-fourth * limit_gua.r
fourth.mask<-fourth
fourth.mask[fourth>= minValue(fourth)] <- 1
#Minor Rivers
minor<-raster("S1_5_8TH.tif")
projection(minor)<-"+proj=longlat +datum=WGS84"
minor<-crop(minor,ext.limit_gua)
minor<-resample(minor,hand.gua, method="bilinear")
minor<-minor * limit_gua.r
minor.mask<-minor
minor.mask[minor>= minValue(minor)] <- 1

#Creat a variables collection

env<-stack(hand,elevation,slope,sea,main,third,fourth,minor)
env.mask<-hand.mask*elevation.mask*slope.mask*sea.mask*main.mask*third.mask*fourth.mask*minor.mask
env<-env*env.mask
names(env)<-c('hand','elevation','slope','sea','main', 'third','fourth','minor')
projection(env)<-"+proj=longlat +datum=WGS84"


plot(hand.gua, main="HAND")


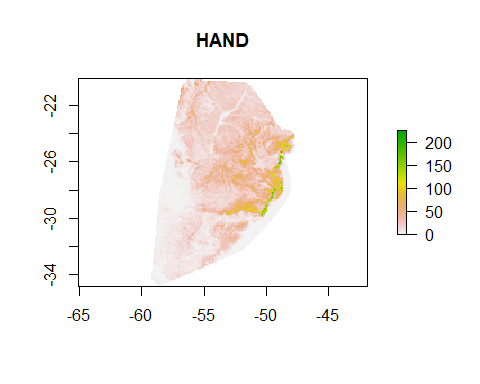


plot(elevation, main="Elevation")


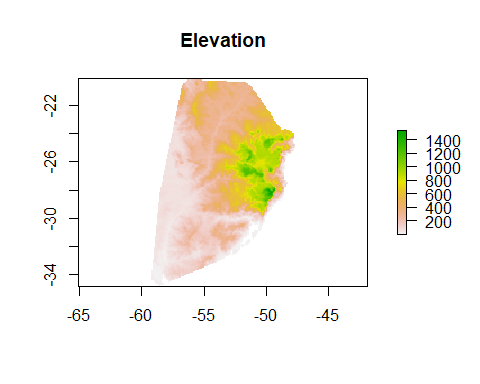


plot(slope, main="Slope")


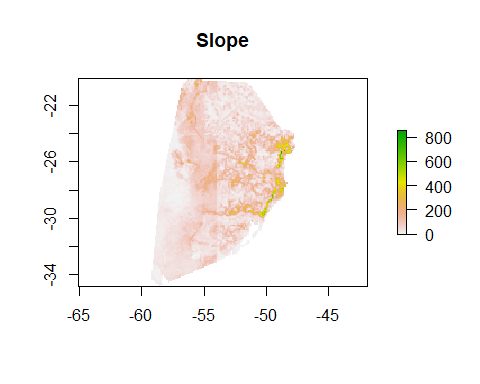


plot(sea, main="Coastline Distance")


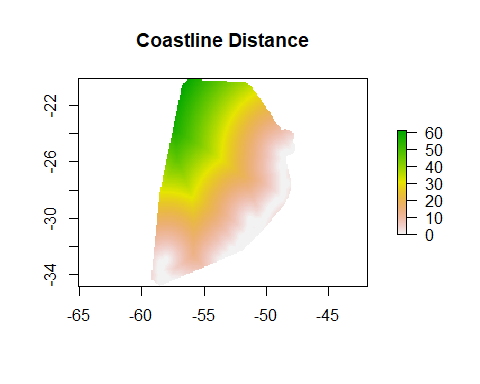


plot(main, main="Main Rivers")


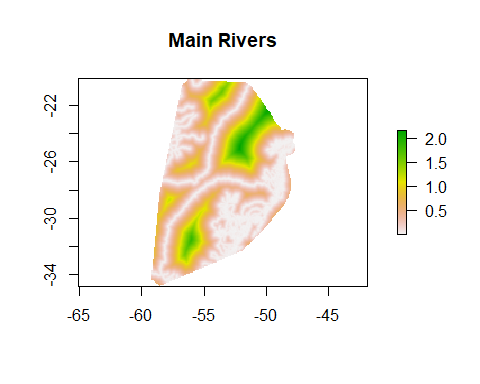


plot(third, main="Third Rivers (intermediate)")


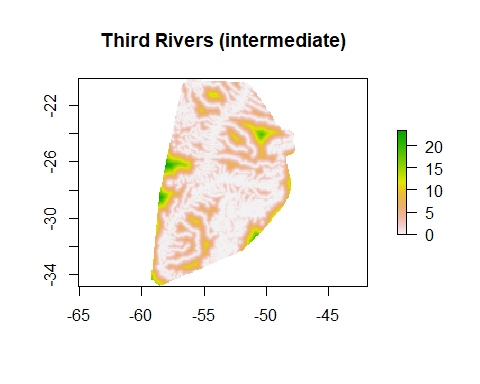


plot(fourth, main="Fourth Rivers (intermediate)")


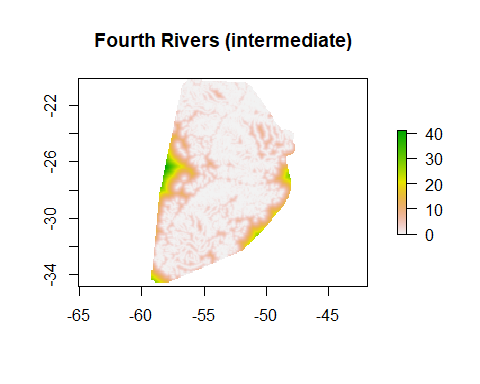


plot(minor, main = "Minor Rivers")


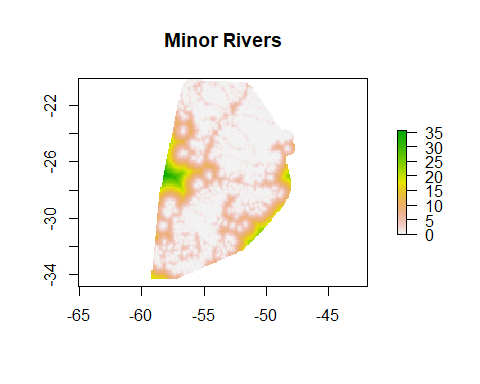


#Import the occurrence points of Guarani Archaeological Sites Guarani Arqueological Occurrence Points are available as *.kmz file, in Bonomo et al(2015) Supplementar Material (doi:0.1016/j.quaint.2014.10.050). We use a Qgis Software to save occurrence points in *.csv format: "S1A_POINTS.csv"

gua_pt<-read.csv("S1A_POINTS.csv", sep=",",dec=".", header=T, row.names=NULL)
gua_pt.spdf<-SpatialPointsDataFrame(gua_pt[,c(1,2)],gua_pt, proj4string=CRS("+init=epsg:4326"))
gua_pt.spdf<-spTransform(gua_pt.spdf, CRS("+proj=longlat +datum=WGS84"))
plot(gua_occ)
plot(gua_pt.spdf, add=T, main="Guarani Occurrence Points")


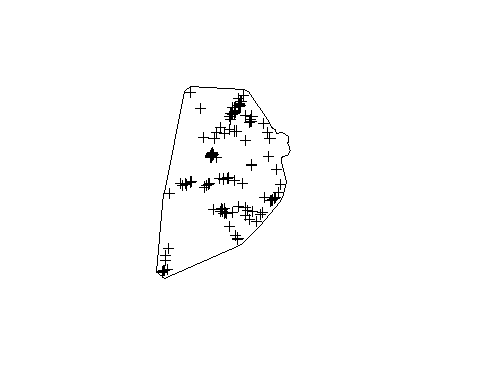


#ENM

library(ENMeval)
bck.na<-env[[1]]
bck.na[]<-NA
r.gua<-rasterize(coordinates(gua_pt.spdf),bck.na,fun='count')
gua.pa<-rasterToPoints(r.gua,fun=function(x){x>0}, spatial=T)
set.seed(1234)
bg<-randomPoints(env[[1]],5000)

#ENMevaluate: In this step, several models are generated, which are ordered to select the models with the highest AUC and the lowest overfitting. It is computationally expensive, so the commands are isolated by "#". Select model is below.
#m1gua<-ENMevaluate(env=env, occ=coordinates(gua.pa), bg.coords=bg, RMvalues=seq(0.5,4,0.5), fc=c("L","LQ","LQP","H","LQH"), method="randomkfold",kfolds=4, parallel = T)

#m1gua@results
#plot(m1gua@results)
#m1gua@predictions
#plot(m1gua@predictions)
#m1gua@models #: list of model objects

#m1gua@occ.pts #: data.frame of occurrence coordinates
#m1gua@occ.grp #: vector of bins for occurrence points
#m1gua@bg.pts #: data.frame of background coordinates
#m1gua@bg.grp

#ord<-order(m1gua@results$avg.diff.AUC, decreasing=T)
#lista<-m1gua@results[ord,c(2,3,13,16,5,7)]
#lista


#Below is the selected model, which presented the best balance between auc and overfitting. # feature = H rm = 2.0 aic = 1910.935 parameters = 20 auc = 0.8037023 diff = 0.03442758

h2<-maxent(env, p=gua.pa, a=bg,
 removeDuplicates=TRUE,
 args=c("-P", 'outputformat=raw', 'noautofeature',
 'nothreshold', 'noproduct','nolinear','noquadratic','noaddsamplestobackground',
 'betamultiplier=2'))

## Warning in .local(x, p, ...): 3 (2.56%) of the presence points have NA
## predictor values

plog.h2<- predict(h2, env,args=c('outputformat=logistic'))
plog.h2.def<-calc(plog.h2, fun=mean)
plot(plog.h2 ,main="Guarani" ,colNA="gray30")


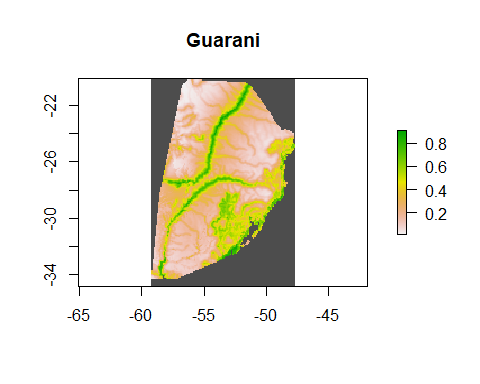


#### S1B Southern-Jê

je_occ<-shapefile("S1B.shp") #Southern-Je occurrence area
je_occ<-spTransform(je_occ, CRS("+proj=longlat +datum=WGS84"))

#Environmental variables
 hand_je<-raster("S1_HAND.tif")##download file in Ambdata
 projection(hand_je)<-"+proj=longlat +datum=WGS84"
 ext.je_occ<-extent(je_occ)
 hand_je<-crop(hand_je,extent(je_occ))
 limit_je.r<-rasterize(je_occ, hand_je)
 hand.je<-hand_je * limit_je.r
 hand.je.mask<- hand.je
 hand.je.mask[hand.je >= minValue(hand.je)] <- 1
#Elevation
elevation_je<-raster("h1k_dem.asc")#download file in Ambdata
 projection(elevation_je)<-"+proj=longlat +datum=WGS84"
 elevation_je<-crop(elevation_je,ext.je_occ)
 elevation_je<-resample(elevation_je,hand_je, method="bilinear")
 elevation_je<-elevation_je * limit_je.r
 elevation.je.mask<-elevation_je
 elevation.je.mask[elevation_je >= minValue(elevation)] <- 1
#Slope
slope_je<-raster("h1k_slope.asc")
projection(slope_je)<-"+proj=longlat +datum=WGS84"
slope_je<-crop(slope_je,ext.je_occ)
slope_je<-resample(slope_je,hand.je, method="bilinear")
slope_je<-slope_je * limit_je.r
slope.je.mask<-slope_je
slope.je.mask[slope_je >= minValue(slope_je)] <- 1
#Coastline Distance
sea_je<-raster("S1_SEA.tif")
projection(sea_je)<-"+proj=longlat +datum=WGS84"
sea_je<-crop(sea_je,ext.je_occ)
sea_je<-resample(sea_je,hand.je,method="bilinear")
sea_je<-sea_je * limit_je.r
sea.je.mask<-sea_je
sea.je.mask[sea_je >= minValue(sea_je)] <- 1
#Water Courses
#1st and 2nd River Classes Distance (Main Rivers)
main_je<-raster("S1_1ST2ND.tif")
projection(main_je)<-"+proj=longlat +datum=WGS84"
main_je<-crop(main_je,ext.je_occ)
main_je<-resample(main_je,hand.je, method="bilinear")
main_je<-main_je * limit_je.r
main.je.mask<-main_je
main.je.mask[main_je >= minValue(main_je)] <- 1
#3rd River Class Distance
third_je<-raster("S1_3RD.tif")
projection(third_je)<-"+proj=longlat +datum=WGS84"
third_je<-crop(third_je,ext.je_occ)
third_je<-resample(third_je,hand.je, method="bilinear")
third_je<-third_je * limit_je.r
third.je.mask<-third_je
third.je.mask[third_je>= minValue(third_je)] <- 1
#4th River Class Distance
fourth_je<-raster("S1_4TH.tif")
projection(fourth_je)<-"+proj=longlat +datum=WGS84"
fourth_je<-crop(fourth_je,ext.je_occ)
fourth_je<-resample(fourth_je,hand.je, method="bilinear")
fourth_je<-fourth_je * limit_je.r
fourth.je.mask<-fourth_je
fourth.je.mask[fourth_je>= minValue(fourth_je)] <- 1
#Minor Rivers
minor_je<-raster("S1_5_8TH.tif")
projection(minor_je)<-"+proj=longlat +datum=WGS84"
minor_je<-crop(minor_je,ext.je_occ)
minor_je<-resample(minor_je,hand.je, method="bilinear")
minor_je<-minor_je * limit_je.r
minor.je.mask<-minor_je
minor.je.mask[minor_je>= minValue(minor_je)] <- 1

plot(hand.je, main="HAND Je")


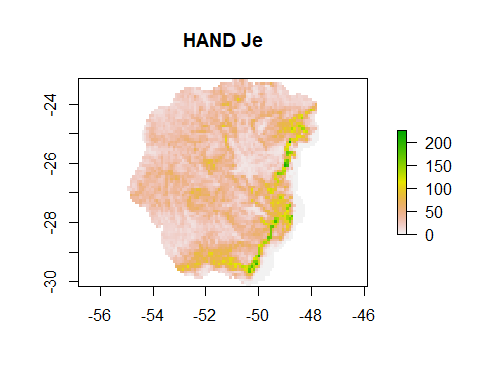


plot(elevation_je, main="Elevation Je")


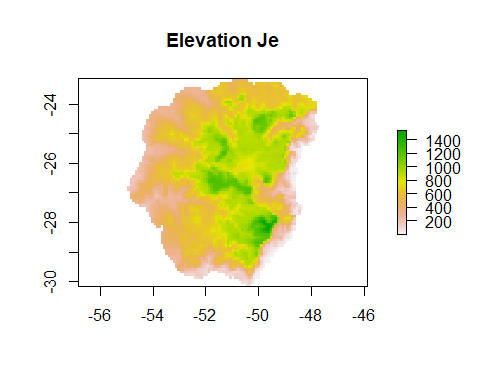


plot(slope_je, main="Slope Je")


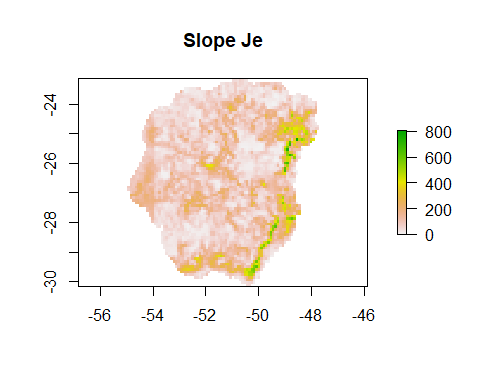


plot(sea_je, main="Coastline Distance Je")


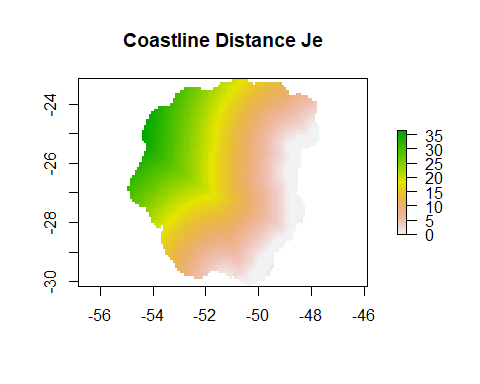


plot(main_je, main="Main Rivers Je")


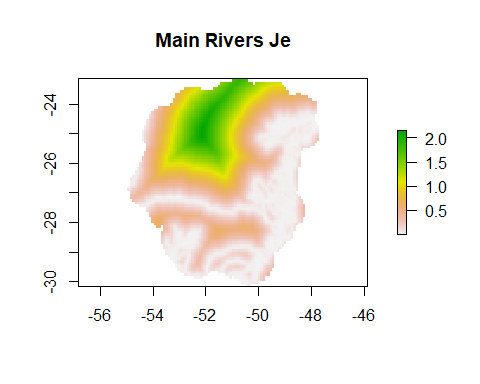


plot(third_je, main="Third Rivers (intermediate Je)")


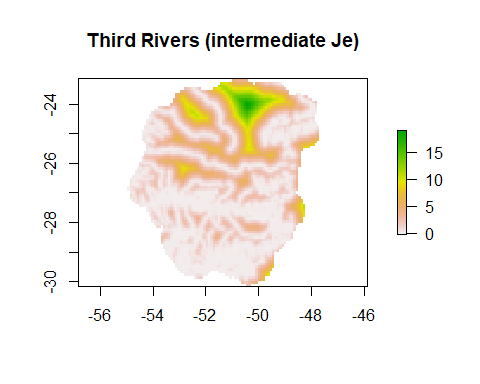


plot(fourth_je, main="Fourth Rivers (intermediate Je)")


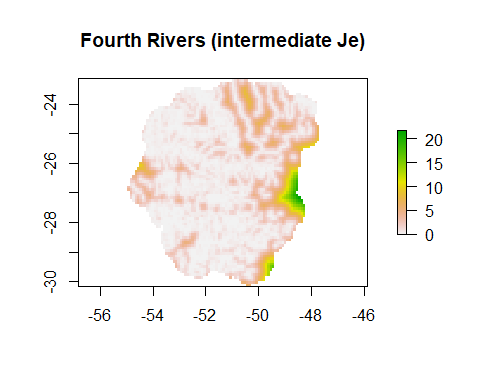


plot(minor_je, main = "Minor Rivers Je")


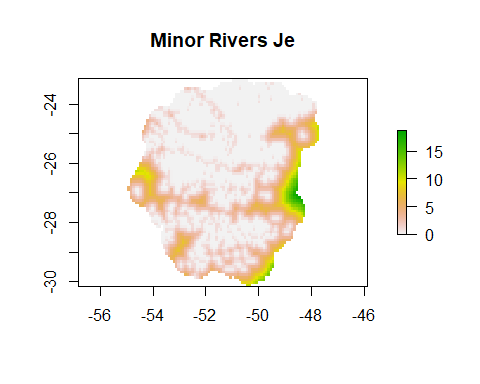


#Creat a variables collection

 env_je<-stack(hand.je,elevation_je,slope_je,sea_je,main_je,third_je,fourth_je,minor_je)
 env.je.mask<-hand.je.mask*elevation.je.mask*slope.je.mask*sea.je.mask*main.je.mask*third.je.mask*fourth.je.mask*minor.je.mask
 env_je<-env_je*env.je.mask
names(env_je)<-c('hand','elevation','slope','sea','main', 'third','fourth','minor')
projection(env_je)<-"+proj=longlat +datum=WGS84"


#Import the occurrence points of Southern-Je Archaeological Sites.We make available a part of the points that we use (those that are already published and that were not given by third parties) in the named file "S1B_POINTS.csv"


je_pt<-read.csv("S1B_POINTS.csv", sep=",",dec=".", header=T, row.names=NULL)
je_pt.spdf<-SpatialPointsDataFrame(je_pt[,c(1,2)],je_pt, proj4string=CRS("+init=epsg:4326"))
je_pt.spdf<-spTransform(je_pt.spdf, CRS("+proj=longlat +datum=WGS84"))
plot(je_occ)
plot(je_pt.spdf, add=T, main="Souther-Je Occurrence Points")


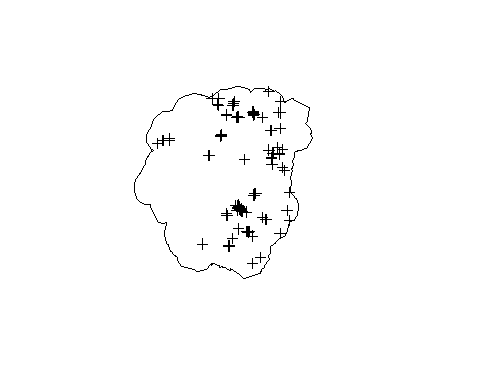


#ENM

library(ENMeval)
bck.na_je<-env_je[[1]]
bck.na_je[]<-NA
r.je<-rasterize(coordinates(je_pt.spdf),bck.na_je,fun='count')
je.pa<-rasterToPoints(r.je,fun=function(x){x>0}, spatial=T)
set.seed(1234)
bg_je<-randomPoints(env_je[[1]],5000)

## Warning in randomPoints(env_je[[1]], 5000): generated random points =
## 0.8836 times requested number

#ENMevaluate: In this step, several models are generated, which are ordered to select the models with the highest AUC and the lowest overfitting. It is computationally expensive, so the commands are isolated by "#". Select model is below.
# mje<- ENMevaluate(env=env_je, occ=coordinates(je.pa), bg.coords=bg_je, RMvalues=seq(0.5,4,0.5), fc=c("L","LQ","LQP", "H", "LQH"), method="randomkfold",kfolds=4, parallel = T)

#mje@results
#plot(mje@results)
#mje@predictions
#plot(mje@predictions)
#mje@models #: list of model objects

#mje@occ.pts #: data.frame of occurrence coordinates
#mje@occ.grp #: vector of bins for occurrence points
#mje@bg.pts #: data.frame of background coordinates
#mje@bg.grp

#ord<-order(mje@results$avg.diff.AUC, decreasing=T)


#Below is the selected model, which presented the best balance between auc and overfitting. # feature = LQP rm = 4 auc = 0.7
lqp.4<-maxent(env_je, p=je.pa, a=bg_je,
 removeDuplicates=TRUE,
 args=c("-P", 'outputformat=raw', 'noautofeature',
 'nothreshold', 'nohinge','noaddsamplestobackground',
 'betamultiplier=4'))

## Warning in .local(x, p, ...): 2 (2.86%) of the presence points have NA
## predictor values

plog.lqp4<- predict(lqp.4, env,args=c('outputformat=logistic'))
plog.lqp4.def<-calc(plog.lqp4, fun=mean)
