# Supplementary figures and images for: Pre-colonial Amerindian legacies in forest composition of southern Brazil
Source: PLoS One. 2020 Jul 23;15(7):e0235819. doi: 10.1371/journal.pone.0235819 (PMC7377383; doi:10.1371/journal.pone.0235819)

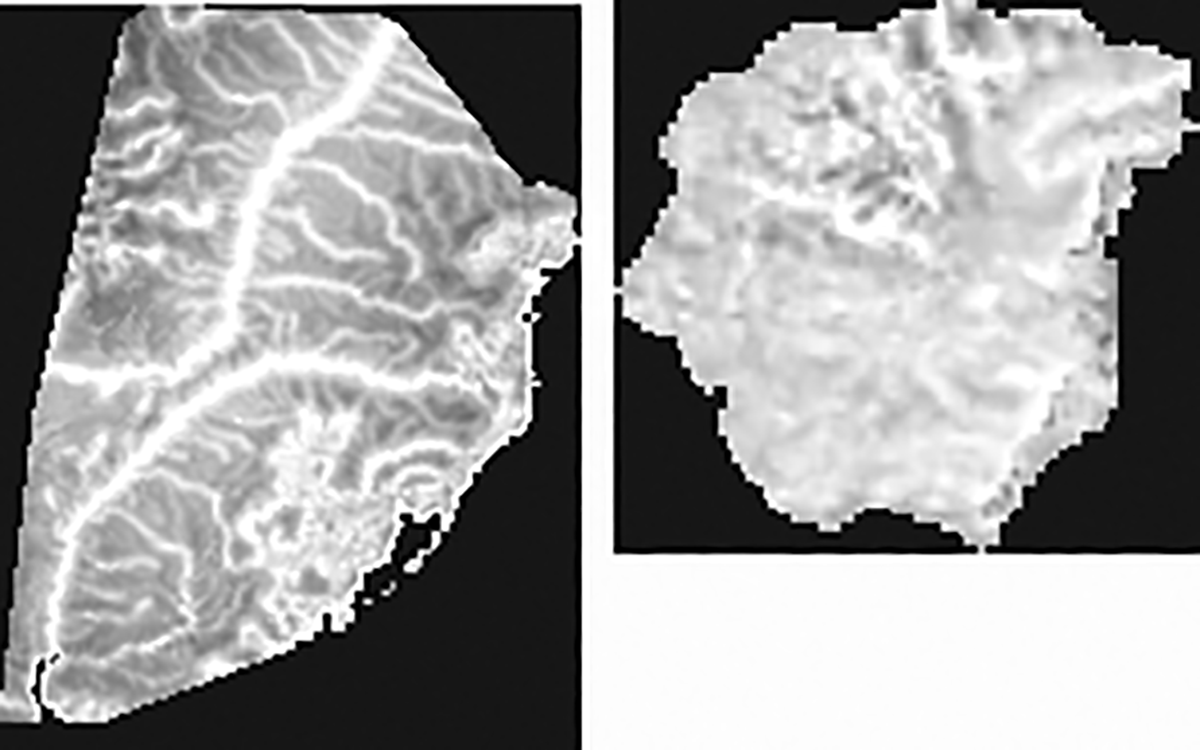

Supplement: S1 Fig — (TIF) [file pone.0235819.s009.tif]
