# Supplementary material for: Pre-colonial Amerindian legacies in forest composition of southern Brazil
Source: PLoS One. 2020 Jul 23;15(7):e0235819. doi: 10.1371/journal.pone.0235819 (PMC7377383; doi:10.1371/journal.pone.0235819)
Supplement: Attachment — Submitted filename: Response to Reviewers.docx [file pone.0235819.s010.docx]

Dear Editors,

We are grateful to you and reviewers for comments regarding our work. The comments were very positive and contributed to improving the manuscript.

In short, we are sharing several files, including all the code we have written, to improve the reproducibility of our analyzes. Changes were made in the manuscript and figures to address the issues raised in review process.

We hope that with the improvements in the manuscript it can be published in Plos One.

Sincerely,

Aline Pereira Cruz

PhD Candidate in Ecology

On behalf of all authors.

**Response to journal requirements:**

**Journal Point 1:** “Please ensure that your manuscript meets PLOS ONE's style requirements, including those for file naming.”

**Response to Journal Point 1:** We check and adjust the style requirements in the manuscript and in the filenames.

**Journal Point 2:** “We note that you have indicated that data from this study are available upon request. PLOS only allows data to be available upon request if there are legal or ethical restrictions on sharing data publicly.”

**Response to** **Journal Point 2:** We organize the data in order to preserve the third-party data, but to allow the analyzes to be reproduced. All steps of the analysis in R, containing the names of the files we are sharing and / or the download paths are in the file “Methods.docx”.

We are sharing two georeferenced images, with the results of niche modeling. The Guarani model is available in the archive “S1A_result.tif” and the Southern-Jê model in file “S1B_result.tif”.

Floristic data are third-party Data. We are sharing the abundance matrix with anonymization of species names and sample units. These data belong to the Floristic Forest Inventory of the State of Santa Catarina (IFFSC), and are published in books, available for download at:

<<https://sites.google.com/view/iffportal/publica%C3%A7%C3%B5es/livros>>.

We requested the abundance matrix (raw data) from the IFFSC coordination, through an email with a description of our research objective. The project coordinator is Dr. Alexander Christian Vibrans, from the Regional University of Blumenau - FURB. Your emails are:[acv@furb.br](mailto:acv@furb.br); [iffsc@furb.br](mailto:iffsc@furb.br); [inventarioflorestal.sc@gmail.com](mailto:inventarioflorestal.sc@gmail.com).

We are sharing part of the geographical coordinates of the Southern-Jê archaeological sites in the named file “S1B_POINTS.csv”. We do not share the portion of the data that is published in Noelli e Souza, 2017, (doi: 10.1590/1981.81222017000100004) and was provided by the authors (thirdy-part data). To request this data, the authors must be contacted by e-mails:

Jonas Jonas Gregorio de Souza <[jonas.gregorio@yahoo.com.br](mailto:jonas.gregorio@yahoo.com.br)>;

Francisco Silva Noelli <[chico.noelli@gmail.com](mailto:chico.noelli@gmail.com)>.

**Journal Point 3:** “We note that Figures 1, 3 and 4 in your submission contain map images which may be copyrighted.”

**Response Journal Point 3:** All figures are original and was created by authors. We don’t utilize proprietary data.

**Response to reviewers:**

We thank the reviewers for the assessment and comments of our manuscript. We have modified the text and figures according to the referees’ critiques. Below are our comments on each issue raised by Reviewer #1.

**Reviewer Point 1:** “This is a very interesting and original research on historical ecology carried out in Santa Catarina State in Brazil. The paper is fairly well organized, the topic is very important and deserves to be published. The Introduction is well-written and interesting. However, in other part the text is sometimes not so clear and there are paragraphs that should be read again to become easy understandable.”

**Response to Reviewer Point 1:** We hope that the text is clearer with the changes. If there is any critical point, please do not hesitate to let us know.

**Reviewer Point 2:** “As a general advice, I suggest to consider some aspects to be incorporated in the paper: a) The chronological framework and diachronical succession of events seems to me not discussed; please, consider that the international audience can be not informed about what means ‘pre-colonial’ – and when the change from the hunter-gatherer to Neolithic culture (transitions or migrations?) had occurred in Brazilian regions.”

**Response to** **Reviewer Point 2:** We agree with the reviewer on this important point about succession of events. We included a timeline (Fig 1, in line 89), with the aim of making the sequence of events in the population of the State of Santa Catarina better understandable.

About culture transitions, research conducted in recent years have pointed to the complexity of the subject due to regional variations, linked to specific cultural histories and the unsuitability of a consolidated basis for the study of European prehistory in the tropics. In general, these studies have shown that the aspects traditionally considered as key elements for this “Neolithic transition”, such as ceramics, sedentary lifestyle, agriculture, do not appear together at once time. There are several examples in tropical regions for a very old presence of ceramics, which, however, does not remain and is constant or in continuous growth, involving, in some regions, production cycles and disappearance. At the same time, there are indications of domesticated items in contexts with dates for the Early Holocene, which are not associated with the presence of ceramics or changes in the pattern of mobility. In other words, these aspects integrate local historical trajectories, with regional variations in chronological and organizational terms. In southern Brazil, what we have identified is a change in the distribution pattern of the sites and their associated archaeological remains from the 3,000 years BP, when a process of cultural interaction is intensified associated with the migration of Jê groups to the southern plateau. However, on the coast, since 7,000 years ago, with intensification between 5-3,000, significant changes involving demography and territoriality are identified and associated with the construction of large shell mounds.

**Reviewer Point 3:** “(…) I suggest to consider some aspects to be incorporated in the paper: (…) b) The demography datum is missed. One should consider that the number of people changed during the different chrono-cultural phases, and therefore when you mention 11,500 BP hunter-gatherers, were they fewer or had a similar impact than the more recent human groups?”

**Response to Reviewer Point 3:** We agree with the reviewer, demography dynamics is important to our discussion. We include demography datum in between lines 370-387. Regarding the impact differences between temporal phases, we expect the marks on the vegetation to be more visible when more recent and / or more persistent. Regarding the persistence of the effects is related to larger population, greater cultural diversity, and more advanced technology. We synthesize the expected effects in a gradient illustrated along the timeline (Fig 1, in line 89).

**Reviewer Point 4** “(…) I suggest to consider some aspects to be incorporated in the paper: (…) c) Are there known differences in the cultural choices (environmental-dependent?) of the human groups involved in the research? (probably they are evident from the Fig. 6) Or the differences are also linked to the changes of one culture along the changing millennia?”

**Response to Reviewer Point 4:** Yes, there are cultural differences related to the choice of environments. In general, both cultural groups prefer proximity to rivers. But “*rivers are especially important to Guarani culture, they are sealers, fishermen*” (added in text in lines 293-294). Southern-Jê prefers highland areas, and “*Araucaria forests are recognized as an important element in the social organization of the current Southern-Jê (Kaingang and Xokleng/Laklaño)*”, i.e, *Araucaria* forests are key in their social organization (added in lines 288-289). In this way, at the Guarani contact Southern-Jê building monument to defend highland territories (were *Araucaria* forest occur), marking a cultural transition: “*Although there are records of Southern-Jê presence in the highlands since 2000 years BP, it is only around 1000 years BP that these groups started to build their funerary earthworks [61,65]. This new architectural expression may be a way of controlling their territory, because it coincides with the arrival of Guarani groups [65]*.” (lines 419-422). In the moment of contact with European immigrants, another change in the distribution pattern occurs. Due to the collapse of populations, areas were opened for re-occupation: *“Following contact with Europeans, Amerindian populations were reduced due to territorial conflicts, diseases, political, and social disruption. There is evidence of Guarani occupation followed by subsequent Southern-Jê occupation in some locations [25], suggesting that the groups competed for the territories”* (lines 424-427). The migration patterns of each group also influence the choice of occupied areas: Guarani along rivers and forming networks, and Southern-Jê in highlands. In manuscript is as follows: “*Guarani groups expanded their occupation along the margins of major rivers and along the coastline [30]. The Jê people were distributed over the highlands in their Brazilian territory [25,26], and their pattern of migration created isolated groups, differing from the Guarani [25]*.” (lines 421-423).

**Reviewer Point 5:** “Line 36 = Araucaria can be added to the Keywords.”

**Response to Reviewer Point 5:** We agree and include *Araucaria* in the key words.

**Reviewer Point 6:** “Lines 68-69 = how long is this ‘long-term’ history? It emerges from the next paragraph that it is about 11,500 years long. Therefore, a more precise chronology can be added, such as changing “identifying many archaeological sites” to “identifying many Holocene archaeological sites”, or something similar.”

**Response to Reviewer Point 6:** We added a timeline (Fig 1) and added “Holocene” in text.

**Reviewer Point 7:** “Line 103 = “humans are cultural niche constructors” = this statement is absolutely sharable and there is a general consensus on this vision, as well as ‘different cultural groups also leave differing floristic composition legacies’ = please note, the presence of archaeological sites and archaeological/archaeobotanical findings is mandatory to infer the type of land use. Further information can be obtained from the ethnobotanical review, as in the discussion of this paper.”

**Response to Reviewer Point 7:** We agree that the description of our hypothesis highlighted aspects that are consensus. We have included an important aspect in the text, about the possibility that there are marks of human actions from the past visible on current forests despite the intense modification in the last century, as in our study area (Sevegnani et al, 2019). In manuscript is as follows: “*our hypothesis is that floristic composition differs in Southern Atlantic Forests with a high probability of past human activities, and that different cultural groups also leave differing floristic composition and abundance legacies notable yet, despite the disruption of these cultural groups and of the intense changes caused by recent populations.*” (lines 112-116).

The gradient parallel to the timeline in Fig 1 illustrates this.

**Reviewer Point 8:** “Line 244 = the first part of the discussion is well centred on geographical and geomorphological characteristics that can explain the observed results and trends. However, the timeline variable – chronology of archaeological sites/events is not reported in the following paragraphs.”

**Response to Reviewer Point 8:** In our analysis, we prioritize the spatial dimension. We ignore the dates because this information is not available for all archaeological sites. Including dates would reduce sampling, making it difficult to calibrate the models. Our results indicate the occupation space accumulated between ~ 3,000-300 years BP. We include this information in the manuscript methods and discussion as bellow: “*We do not consider the temporal information of archaeological sites, as this would considerably reduce the sample size*.” (lines 156-158) and “*Our maps explain the spatial dimension of the occupation of southern-Jê and Guarani, but the temporal dimension mixes the entire period of pre-Colonial occupation*.” (lines 412-414).

**Reviewer Point 9:** “Line 253 = ‘Alternatively, the species could’ = Alternatively, the plant species could.”

**Response to Reviewer Point 9:** We agree and modify the text.

**Reviewer Point 10:** “Line 255 = as an example on the need of chronological details, in the sentence ‘Amerindians intentionally cleared the land and used fire to produce crops,’, you should add the culture and/or Holocene period or millennia BP.”

**Response to Reviewer Point 10:** We agree and modify the text. We highlighted in text the moment when fire started to be used to open clearings: “*In the State of Santa Catarina palynological evidence indicates that the opening of clearings for cultivation started between 3760 and 2430 B.P (Fig 1) [34]*” (lines 269-271).

**Reviewer Point 11:** “Line 264 = “may have involved selective extraction of species for fuel” = is this a speculative argument or can you make an example from a case study?”

**Response to Reviewer Point 11:** We agree and include study case examples: “*Fuel for funeral use must reach very high temperatures, for domestic use it must be easily controllable [63]. The use of fire in rituals can also lead to the selection of woods by symbolic characteristics. Archaeological research has shown that Southern-Jê used preferred species in contexts that indicate the practice of rituals: Araucaria angustifolia, and Jacaranda sp. in a location where Araucária is not available* [64].”(lines 278-281).

**Reviewer Point 12:** “Lines 279-310 = This is an interesting ethnobotanical part of the text, but it is not clear in which order the plants/uses are discussed, and why they are listed.”

**Response to Reviewer Point 12:** We rearranged the paragraph. Now the text follows a sequence from left to right along the gradient illustrated in Fig 7 (it was Fig 6). That is, starting with species related to the Southern-Jê occupation and ending with species related to the Guarani occupation. The species are listed in Fig 7. We have included captions in the text and in the figure to facilitate the location of each species. For some of the listed species, we discussed ecological and utilitarian features that can help us understand this gradient. We also highlight those species that have an indigenous reference in their names.

**Reviewer Point 13:** They “Line 285 = phytolits in pottery could suggest consumption or, preferably, are they evidence of plant processing?”

**Response to Reviewer Point 13:** They could be evidence of processing. We modify the text.

**Reviewer Point 14:** “Line 385 = practical”

**Response to Reviewer Point 14:** We agree and modify the text.

**Reviewer Point 15:** “Citations 31. 32, 33, 77 = put ‘Aracauria’ in italics.”

**Response to Reviewer Point 15:** We agree and modify the text.

**Reviewer Point 16:** “Citation 37, 55 = check the format.”

**Response to Reviewer Point 16:** We agree and modify the text.

**Reviewer Point 17:** “Fig. 6 = It is an interesting figure (please, quote it in the text) but I am not sure that it is sufficiently clear the sense and aim of this ‘separation of plant species’ – Cultural? Phytogeographical? Current or past different exploitation? The full name of species should be preferred to the abbreviation of the genus, if possible.”

**Response to Reviewer Point 17:** We quote Figure 7 (it was 6) in text. The figure shows a list of species with RDA scores above |0.1| and organizes them along a cultural gradient between Southern-Jê and Guarani. Thus, we show species that has its distribution associated with the distribution of archaeological sites Southern-Jê and Guarani. Species can also be related to the environmental characteristics of the archaeological sites’ specific location. They could be yet related with an interaction of environment and occupation. There are many factors that influence the distribution of species and the causes cannot be explained by our analyzes. We highlight this in the manuscript as follow: “An important point is that our analyzes do not isolate the causes of the distribution of plant species, we are considering niche construction as a process in which factors of different natures are inseparable.” (lines 300-301).
