# Supplementary material for: Pre-colonial Amerindian legacies in forest composition of southern Brazil
Source: PLoS One. 2020 Jul 23;15(7):e0235819. doi: 10.1371/journal.pone.0235819 (PMC7377383; doi:10.1371/journal.pone.0235819)
Supplement: Attachment — Submitted filename: Response to Reviewers.docx [file pone.0235819.s011.docx]

Dear Editors,

We are grateful to you and to reviewers for comments regarding our work. Changes were made in the manuscript and figures to address the issues raised in review process.

We hope that this revised manuscript is suitable for publication in PlosOne,

Sincerely,

Aline Pereira Cruz

PhD Candidate in Ecology

On behalf of all authors.

**Response to journal requirements:**

**Journal Point:** “After carefully revising your resubmission, we are quite reaching a final decision that is now conditioned to a few details to be changed in your manuscript. Both reviewers and I agreed that your manuscript is an interesting contribution for PlosOne and, for final acceptance, we kindly ask for the following (as pointed out by Dr. Mercuri):”

**Response to Journal Point:** We are happy about the final decision is conditioned to a few details. All questions pointed By Dr. Mercuri was check and fixed as described below.

**Response to reviewer:**

We thank the Dr. Mercuri for the assessment and comments of our manuscript. We have modified the text and figures according to the referees’ critiques. Below are our comments on each issue raised by Dr. Mercuri.

**Reviewer Point 1:** “Line 80 = “Zea Mays, Manihot Esculenta, and Dioscorea sp,” = “*Zea mays*, *Manihot esculenta*, and *Dioscorea* sp.””

**Response to Reviewer Point 1:** We fix it.

**Reviewer Point 2:** “Line 123 = delete comma after ‘(SC)‘”
**Response to** **Reviewer Point 2:** We delete the comma.

**Reviewer Point 3:** “Lines 157-158 = “We do not consider the temporal information of archaeological sites, as this would considerably reduce the sample size.” This sentence was added now to reply to comments by the reviewer, however it may introduce other criticisms: the chronology is a must in archaeology. Probably, it may be better changing the sentence by reporting only the information on the range of chronology of the sites studied, that is: “the archaeological sites have been occupied during the last XX millennia””.

**Response to Reviewer Point 3:** We agree about the high importance of chronology in archaeology and modify the text as suggested. We removed the sentence that might sound while minimizing the importance of the chronology: “We do not consider the temporal information of archaeological sites, as this would considerably reduce the sample size.”. And we include the sentence “The selected archaeological sites have been occupied over the past 3,000 years” (line 159-160).

**Reviewer Point 4: “**Line 269 = “unintentionally favored certain plants and/or crops by…” = ‘certain’ is vague and, if correct, ‘synanthropic’ (or the name of the species) should be preferred”.

**Response to Reviewer Point 4:** We agree and remove the word “certain”. We include an example: “… and unintentionally favored plants by interrupting seeds dormancy, like *Mimosa scabrella* [59–61], by improvement in light availability to pioneer species, like *Solanum mauritianum* [60].”.

**Reviewer Point 5:** “Line 359 = check “and we need””.

**Response to Reviewer Point 5:** We fix it.

**Reviewer Point 6:** “Fig. 4 = check the green colours a sit seems that the colour of Brazil and it he S. America are inverted, especially in the part ‘ c ‘ of the figure.”

**Response to Reviewer Point 6:** We fix it.
